# Discovery of potential FGFR3 inhibitors via QSAR, pharmacophore modeling, virtual screening and molecular docking studies against bladder cancer

**DOI:** 10.1186/s12967-023-03955-5

**Published:** 2023-02-10

**Authors:** Mahmoud Ganji, Shohreh Bakhshi, Alireza Shoari, Reza Ahangari Cohan

**Affiliations:** 1 Department of Medical Biotechnology, Faculty of Medical Sciences, Tarbiat Modares University, Tehran, Iran; 2 Faculty of Pharmacy, Tehran University of Medical Sciences, Tehran, Iran; 3 Biotechnology Research Center, Pasteur Institute of Iran, Tehran, Iran; 4 Department of Nanobiotechnology, New Technologies Research Group, Pasteur Institute of Iran, No. 69, Pasteur Ave, Tehran, 1316543551 Iran

**Keywords:** Bladder cancer, Drug discovery, Pharmacophore, QSAR, Docking, Molecular dynamics

## Abstract

**Background:**

Fibroblast growth factor receptor 3 is known as a favorable aim in vast range of cancers, particularly in bladder cancer treatment. Pharmacophore and QSAR modeling approaches are broadly utilized for developing novel compounds for the determination of inhibitory activity versus the biological target. In this study, these methods employed to identify FGFR3 potential inhibitors.

**Methods:**

To find the potential compounds for bladder cancer targeting, ZINC and NCI databases were screened. Pharmacophore and QSAR modeling of FGFR3 inhibitors were utilized for dataset screening. Then, with regard to several factors such as Absorption, Distribution, Metabolism, Excretion and Toxicity (ADMET) properties and Lipinski’s Rule of Five, the recognized compounds were filtered. In further step, utilizing the flexible docking technique, the obtained compounds interactions with FGFR3 were analyzed.

**Results:**

The best five compounds, namely ZINC09045651, ZINC08433190, ZINC00702764, ZINC00710252 and ZINC00668789 were selected for Molecular Dynamics (MD) studies. Off-targeting of screened compounds was also investigated through CDD search and molecular docking. MD outcomes confirmed docking investigations and revealed that five selected compounds could make steady interactions with the FGFR3 and might have effective inhibitory potencies on FGFR3.

**Conclusion:**

These compounds can be considered as candidates for bladder cancer therapy with improved therapeutic properties and less adverse effects.

**Supplementary Information:**

The online version contains supplementary material available at 10.1186/s12967-023-03955-5.

## Introduction

Cancer is still an increasing severe public health risk worldwide. Consistent with the most recent global cancer data in 2020, there were 18.1 million new cases and 10.0 million deaths related to cancer [1]. Regardless of remarkable progress of different therapeutic approaches such as surgery, radiotherapy and chemotherapy, cancer takes the lives of numerous people all around the world [2].

As a kind of urothelial cancer, bladder cancer is the utmost prevalent malignancy comprising the urinary system and the ninth most common malignancy worldwide [3]. Histologically, bladder cancer can categorize into three main kinds: metastatic bladder cancer, muscle invasive and non-muscle invasive [4]. While the 5-year survival rate of non-muscle invasive bladder cancer is over 70%, it recurs recurrently or developments to muscle invasive bladder cancer [5]. Moreover, the therapy results persist inadequate for metastatic bladder cancers and muscle invasive, with a 5-year survival rate of less than 50% [6]. The basic treatments for metastatic bladder cancers and muscle invasive are systemic administration of chemotherapeutic agents and total bladder resection, while these therapies are accompanying with side effects and low efficacy [7]. Lately, immune checkpoint inhibitors, for example anti-PD-L1 and anti-PD-1 antibodies, have been approved for the metastatic urothelial carcinoma treatment [8]. Although multiple complete response cases have been reported in clinical trials, their objective response rates were just 14–24% [9]. Therefore, novel safer and effective molecularly targeted treatments for bladder cancer are necessary. There are several indications that enzymes play a vital role in the regulation of signaling pathways of the enormous majority of cancers [10].

One of the most vital cell signaling proteins for progression from early embryogenesis to the development of different organs is fibroblast growth factors (FGFs) and their receptors, fibroblast growth factor receptors (FGFRs) [11]. The FGF/FGFR family in mammalian exerts actions through 4 different isoforms (FGFR1, 2, 3 and 4). FGFR is a receptor tyrosine kinase which expressed as a single-transmembrane receptor [12]. FGFR dimerization, which induced via FGFs, subsequently leads to FGFR autophosphorylation and activation of downstream signaling pathways such as Ras/Raf/MEK/MAPK and PI3K-AKT [13]. In plenty of human tumors, abnormal activation of FGF/FGFR signaling stimulates angiogenesis, migration, invasion, and cellular proliferation. Genetic alterations including point mutation, chromosomal translocation/fusion and gene amplification, can lead to unusual ligand-dependent signaling and aberrant activation of FGFRs, which can conceivably cause tumor development [14]. For example, as one of the potential driving oncogenes, FGFR3 point mutations have been detected to be increased in bladder cancer [15]. In 2019, Erdafitinib (JNJ-42756493), as FGFR3- targeted therapy, has obtained accelerated approval from the FDA (U.S. Food and Drug Administration) for the treatment of adults with metastatic or locally advanced urothelial carcinoma [16].

According to these outcomes, FGFR3 is considered a noteworthy aim for new treatment for bladder cancer. Generally, due to the high conservation of amino acid residues in the kinase catalytic domain, attaining specific ATP-competitive kinase inhibitors is challenging [17]. Additionally, low kinase selectivity will lead to side effects and thus classical FGFR inhibitors have displayed toxicity [18].

As an efficient computational tool, ligand based pharmacophore modeling by 3-Dimensional Quantitative Structure Activity Relationship (3D-QSAR) pharmacophore modeling is broadly utilized in virtual screening along with designing inhibitors and drugs [19]. QSAR model, which measures the connection among biological activities and structures of a series of compounds, is based on the hypothesis that compounds with physiochemical properties or similar structures have similar activities [20]. The QSAR model development contains a series of successive steps, such as: (i) choice of Data set and elicitation of descriptors representing the molecule, (ii) variable selection, (iii) construction of the model, and (iv) QSAR model validation [21]. Just limited investigations on FGFRs utilizing the QSAR method have been performed by now. For instance, Zhou et al*.* performed pharmacophore modeling and 3D-QSAR analyses for different chemical classes of FGFR1 inhibitors, and developed a combinatorial pharmacophore-based 3D QSAR model [22]. In 2020, Kuriwaki and colleagues conducted a structure-based drug design and reported the synthesis and structure–activity relationships (SARs) of pyrimidine derivatives and 1,3,5-triazine as highly selective and effective FGFR3 inhibitors [23].

In the current study, ZINC and NCI databases were screened to find the potential compounds for bladder cancer targeting. Pharmacophore and QSAR modeling of FGFR3 inhibitors, as well as the pharmacokinetics and toxicity parameters were employed for dataset screening. Finally, molecular docking and MD simulations were utilized to investigate the interaction between the screened compounds and the target. Off-targeting of screened compounds was also investigated using CDD search and molecular docking.

## Materials and methods

### Target identification for bladder cancer

In order to find the potential protein targets involved in the pathogenesis of bladder cancer, OMIM (https://www.omim.org/), Disgenet (https://www.disgenet.org/), Pharos (https://pharos.nih.gov/), Drug bank (https://go.drugbank.com/), and KEGG (https://www.genome.jp/kegg/) servers were used. Then, the amino acid sequence and 3D-structure of identified targets were retrieved from Uniprot (https://www.uniprot.org/) and protein data bank (https://www.rcsb.org/) servers, respectively. The quality of 3D-structures was checked using PROCHECK (https://servicesn.mbi.ucla.edu/PROCHECK/) and ProSA (https://prosa.services.came.sbg.ac.at/prosa.php) web servers. The topology of the potential targets was examined using Uniprot server.

### Dataset screening

**Fig. 1:**
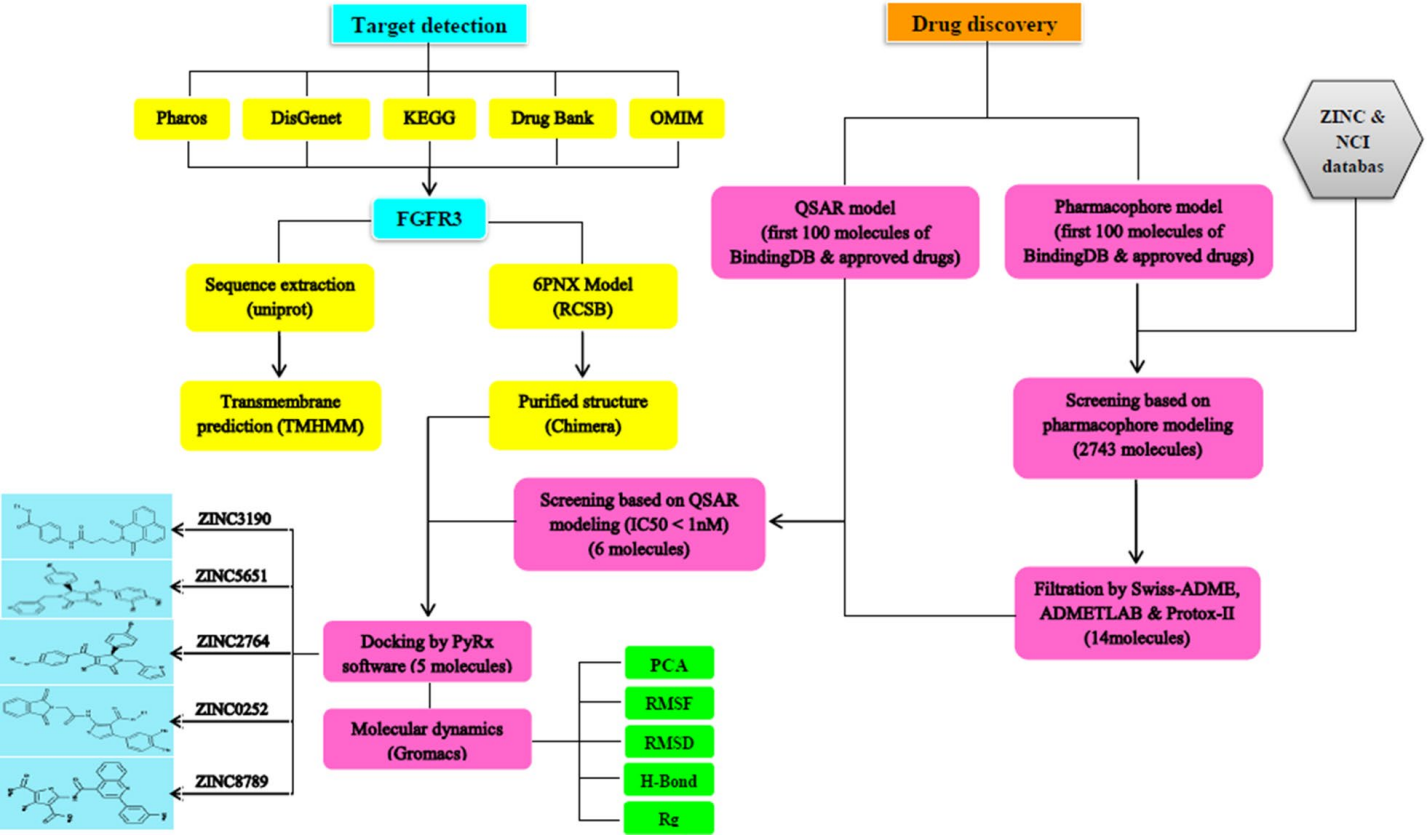
The schematic diagram of screening steps used in this study

Zinc12 (https://zinc12.docking.org/) and NCI (https://www.cancer.gov/about-nci) databases were used as databases containing potential lead compounds. The following steps were employed to screen the compounds. At first, the compounds were screened by a generated pharmacophore model based on 6PNX structure with a resolution of 2.20 Å [24]. Then, the pharmacokinetic parameters including absorption, distribution, metabolism, and excretion, as well as the toxicity were considered to filter the compounds. To select the potent compounds a QSAR model was generated [25]. The selected hits were then docked with the FGFR3, and finally the docked structures were subjected to the molecular dynamics to analyze the interaction between hits and FGFR3. The schematic diagram of screening steps is depicted in Fig. 1.

### Pharmacophore modeling and screening

In order to generate a pharmacophore model, the first 100 molecules related to *fgfr3* gene were extracted from binding database (https://www.bindingdb.org/bind/index.jsp) according to their IC50 values. Four approved drugs that are effective in FGFR3 inhibition, namely Ponatinib (BD50322535), Nintedanib (BD50026612), Pemigatinib (BD86705695), and Infigratinib (BD50355393) was used along with the relevant conformers for benchmark datasets [26, 27]. Schrödinger software version 2018 (Schrödinger, LLC, New York, NY) was used for modeling and screening of pharmacophore model. In the LigPrep module [28], the OPLS_2005 force field [29] and the ionization section were set to the “Do not change” option. Also, desalt, tautomerization, and computation was determined according to the software default. Given that Maestro format is the ideal and readable mode for Schrödinger software, this format was selected from the output directory. In the Develop Pharmacophore model module, the hypothesis match was set to at least 50% and the Generate Conformer and Minimize output conformer option was activated. Then, the molecules obtained from Zinc12 and NCI databases were converted to Maestro format using the Create Phase Database module [30], and from the Ligand filtering directory the Generate Qikprop properties and Prefilter by Lipinski’s Rule options were activated [31, 32]. Next, from the Input section, the target number of conformers was considered equal to 10 and the Minimization option was selected for screening process.

### ADMET screening

The lack of effectiveness and the toxicity are main reasons for the failure in the field of drugs research and development (R&D failure). This is largely related to pharmacokinetic properties such as absorption, distribution, metabolism, exertion, and toxicity. Therefore, the study of these properties can play an effective role in reducing the failure rate in the design and development of drugs. Thus, the pharmacokinetic properties of the ligands obtained in the previous step were predicted using ADME servers (http://www.swissadme.ch/) and ADMETLAB (https://admet.scbdd.com/). Moreover, since the toxicity level of drugs has a vital importance in the field of human and animal health, the Protox-II server (https://tox-new.charite.de/) was used to further evaluation of the toxicity.

### QSAR modeling and screening

PaDEL-Descriptor software was used to generate the molecular descriptors of the candidates obtained from Zinc, NCI, and Binding databases [33]. In this software, the descriptors were defined based on 1D and 2D and the standardize section was set to the default. Subsequently, SMLR software was used to select the most important molecular descriptors. Data pre-treatment, α-values, and process validation sections were activated. In the data pre-treatment section, the variance cut-off and the inter correlation cut-off was set to 0.001 and 0.9, respectively. The α-values section also includes two parameters, alpha-to-enter and alpha-to-remove, which both were set to 0.15. In the process validation section, the value of random model generation was set to 10. To generate the QSAR model, 109 molecules were considered in which the first 100 of molecules with the lowest IC50 were selected from Binding database and 9 molecules were related to the four FDA-approved drugs and their conformers. These molecules were used as input data to Chemoface software [34]. The PLS algorithm was selected from the regression method section for model generation and screening [35]. The molecular index obtained from SMLR software was given to the software as X-axis data and IC_50_ of these molecules was considered as Y-axis data. For model validation, 20% of 109 molecules (22 molecules) were provided to the software for Kennard-Stone test. After QSAR model generation, molecules which obtained from Zinc12 and NCI databases were screened.

### Molecular docking and virtual screening

Docking is a molecular modeling process that uses certain algorithms to predict the orientation and reciprocal position of two molecules involved in a complex [36]. Among the various methods available for docking, PyRx software, a useful tool for computational drug discovery and screening of compounds against potential drug targets, was selected [37]. At first, the screened molecules were converted to Pdbqt format, and FGFR3 protein was added as a target for the docking process. FGFR3 protein was prepared for docking via Chimera software by selecting Dockprep and structure minimization options [38]. In the Dockprep step, all non-complexed ions and solvent molecules were removed and hydrogen atoms and charges were added to the structure. The charge was assigned by the Gasteiger option. In the minimization step, all settings were set to default. Also, all screened molecules with pdbqt format were subjected to energy minimization and the process of docking was performed under 8 exhaustiveness. Besides, the grid volume of the box plot was selected to be 43.8219, 58.4611, and 54.2448 Å for x, y, and z dimensions, respectively. The grid center was fixed to 4.8214, − 20.485, and − 20.1315 Å x, y, and z dimensions, respectively. Finally, the binding affinity potency of each ligand was predicted using the Vina wizard.

It should be notified that angiogenesis in bladder cancer is induced by the mediation of various molecules, which is the result of an intricate balance between proangiogenic and antiangiogenic factors. Among the proteins involved in this pathway, EGFR and ERBB2 are also considered as the main factors in starting this process [39]. Therefore, the potential binding capability of screened compounds with EGFR and ERBB2 could have high importance in the treatment of bladder cancer. For this purpose, 3D-structures of EGFR (RCSB: 5UG9) and ERBB2 (RCSB: 3PP0) proteins were retrieved from PDB server, purified, and prepared by Chimera software same as the process of docking for FGFR3. Utilizing PyRx software, the final candidates were subjected to energy minimization and different protein binding conformations (Exhaustiveness) were set to 8. Furthermore, the grid volume for EGFR was 43.6805, 49.1492, and 59.4835 Å (x, y, and z dimensions), and the grid center was set to − 3.5551, 18.8716, and − 21.5603 Å (x, y, and z dimensions). The grid volume and the grid center for ERBB2 were set to 59.7556, 47.6110, and 57.5142 Å (x, y, and z dimensions) and 12.4713, 21.6808, and 34.0837 (x, y, and z dimensions), respectively. The level of binding affinity of each ligand was predicted using the Vina wizard. The interaction of residues involved in the active sites of FGFR3, EGFR, and ERBB2 with ligands was analyzed at 3D and 2D levels using Pymol and Ligplot software, respectively.

### Molecular dynamics simulation

Molecular Dynamics (MD) simulation is a computational approach for evaluating the physical movements of atoms and molecules. The atoms and molecules are permitted to engage for a fixed period of time, giving a view of the dynamic “evolution” of the system. For this purpose, GROMACS 2019.1 software (https://manual.gromacs.org/documentation/2019.1/download.html) [40] and Amber03 force field were used to simulate the protein–ligand complex for 100 ns. The topology of the ligands was generated using GAFF force field in the antechamber module of Amber tools software. After that, Python (ACPYPE Script) was used for converting the format of topologies to the format which is matched with GROMACS. The SPC model was used for water molecules and the box dimensions were considered 8.628 × 8.628 × 8.628 nm. The distance of FGFR3 protein from the box walls was set to 1 nm. Four sodium ions were added to the box in order to neutralize the system and the number of water molecules in the box was determined 19,549. The systems were energy minimized in 50,000 steps using the steepest descent algorithm while holding the solute frozen. The periodic boundary condition was applied in all directions. Heat equilibration step using the Berendsen thermostat [41] and constant temperature equilibrium of 1 ns at 300° K were considered. The pressure balancing step was performed using a Berendsen barostat for a pressure of 1 bar for 1 ns. In this process, the bond lengths were constrained with LINCS algorithm [42], while SETTLE algorithm was used for water restrain. Particle-mesh Ewald (PME) [43] was applied to calculate electrostatic forces. A cut-off of 10 Å was considered for short-ranged interactions, whereas 14 Å cut-off was fixed for Van-der-Waals interactions.

Finally, the trajectories were analyzed in terms of Rg, RMSD, RMSF, H-bond, and principal component analysis (PCA) factors using GRACE software [44]. Moreover, Prodigy server (https://wenmr.science.uu.nl/prodigy/) was used to evaluate the Gibbs free energy (ΔG) between the ligands and FGFR3 [45, 46]. In this server, the prediction was performed based on the number of atomic contacts (ACs) with a distance threshold of 10.5 Å and categorized the ACs according to atoms that are engaged with interaction. Firstly, the ΔG of docked structures was calculated before MD. After MD simulations, the PDB structures were extracted from the last frame of simulations (100 ns). The ΔG of each complex was measured again. Finally, the 3D and 2D structures of each complex (before and after molecular dynamics) were analyzed using Pymol software (at a range of 3 Å) and Ligplot (at the software default), respectively.

### Off-targeting

The relationship between the screened compounds and non-target proteins was examined. The similarity between FGFR3 sequence and other proteins was studied using CDD server (https://ncbi.nlm.nih.gov/Structure/cdd/cdd.shtml). Also, due to the high affinity of Ponatinib and Nintedadinib to FGFR3, the interaction of other proteins (ABL1, KIT, KDR, FLT3, and RET) with these drugs was evaluated by STITCH server (http://stitch.embl.de/). The molecular docking of these drugs and the screened compounds was performed using the PyRx software. Finally, the residues involved in the interactions were analyzed in 2D and 3D levels using Ligplot and Pymol, respectively.

### The potential effect of screened compounds on other cancers

To investigate the potential interaction of the screened compounds with HRAS, KRAS, and RB1 proteins, molecular docking was carried out. The structure of proteins was retrieved from PDB (HRAS: 2CE2, KRAS: 6TAN, RB1: 2R7G). Then, the structures were cleaned and energy minimized using Chimera. Finally, the binding affinities were measured using PyRx and the interactions were analyzed using Pymol [47].

## Results and discussion

### Target identification for bladder cancer

Initial screening by the OMIM server indicated that FGFR3, HRAS, KRAS, and RB1 proteins can be potentially important in bladder cancer. However, KRAS and RB1 proteins have not been validated after reviewing by Disgenet, KEGG, and Pharos servers (Additional file 1: Table S1). Moreover, no drug found for RB1 in Drug bank, Pharos, and KEGG drug databases and in the case of KRAS, only one drug (Sotorasib^®^, Amgen) found in Drug bank for the treatment of non-small cell lung, colorectal, and appendix cancers. Both FGFR3 and HRAS proteins have been proposed as targets by the servers, however, only FGFR3 has provided reliable results. Checking the KEGG and Disgenet databases indicated that besides bladder cancer, FGFR3 also has a significant role in the pathogenesis of other cancers, and therefore, FGFR3 targeting could also be considered for treatment of several cancers. Among the screened targets, FGFR3 had the highest disease specificity index (DSI) and a high Scoregda index after HRAS protein (Additional file 1: Table S2). Also, the number of researches for FGFR3 is higher than HRAS in PubMed (94 for FGFR3 versus 29 for HRAS). Based on the results, FGFR3 was selected as the target protein and its approved drugs such as Ponatinib (BD50322535), Pemigatinib (BD86705695), Nintedanib (BD50026612), and Infigratinib (BD50355393) were retrieved from Pharos and Drug bank and bindingDB databases.

### FGFR3

**Fig. 2:**
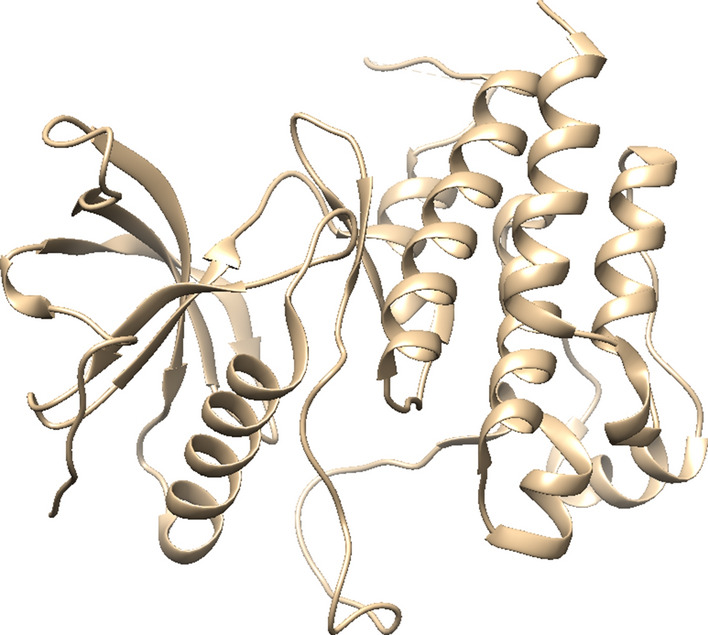
Final model proposed for FGFR3 protein using RCSB server

**Fig. 3:**
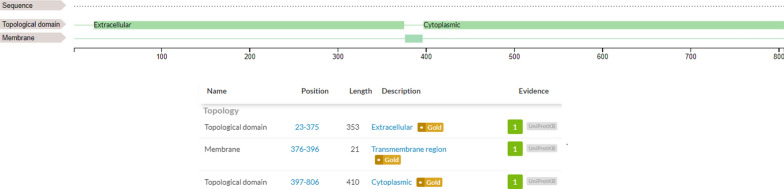
FGFR3 protein domains and structure in cell membrane

FGFR3 is pharmacologically involved in the initial phosphorylation of RAS-MAPK [11] cascade that eventually leads to proliferation, differentiation and cell metastasis (Additional file 1: Fig. S1). Structurally, the FGFR3 is a membrane protein that composed of alpha helix (α-helix), extended beta strand, and random coil [48] (Additional file 1: Fig. S2). The helical structures (composed of alpha and beta-sheets) and random coil provide the stability and the flexibility of the protein, respectively [49]. Topologically, the protein contains an extracellular domain (23–375), a transmembrane domain (376–396), and an intracellular domain (397–806). The binding domain (residues 478–508) and the active site (residues 613–625) of protein are located in the intracellular domain [50] (Fig. 2). The PDB structures of FGFR3 were analyzed based on their resolution and coverage on active and binding sites and among all available structures the PDB structure of 6PNX with Resolution 2.20 Å was selected and extracted from RCSB server (Fig. 3), Phi and psi analysis of this structure revealed that 91.8% residues (234 AA) are present in the favored region, 7.5% (19 AA) residues are in the allowed region, and 0.8% residues (2 AA) are in the outlier region (Additional file 1: Fig. S3). Structure assessment by ERRAT indicated that most residues are < 95%, which is in a reliable range, and just a limited numbers of residues are between 95 and 99% (yellow and red colors). The Quality Factor is calculated as 96.512 that is a favored value for this model (Additional file 1: Fig. S4). The Z-score of − 8.31 predicted by ProSA represents a good quality for the model (Additional file 1: Fig. S5). The Z-score indicates the overall quality of model and measures the deviation of total energy of a structure with respect to an energy distribution derived from random conformations [51].

### Dataset

A 6614 ligand complex from Zinc12 and a 5996 molecular complex from the NCI (National Cancer Institute) database were used for this purpose. It should be noted that the ligands received from Zinc12 were selected based on the Drug likeness.

### Pharmacophore modeling and screening

**Fig. 4:**
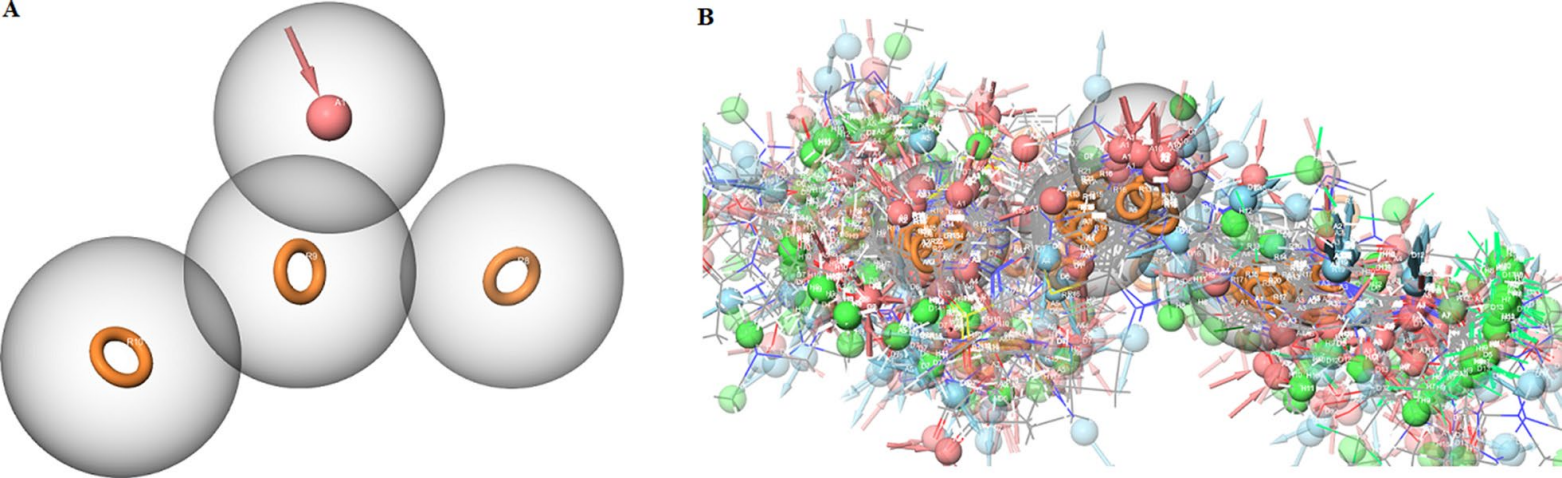
Illustrations for modeling and screening. **A** Pharmacophore hypothesis model ARRR-1 for FGFR3 inhibitor. **B** Arrangement of individual features in a fixed distance of pharmacophore hypothesis model ARRR-1 for FGFR3 inhibitor

After evaluating the pharmacophore models, the ARRR-1 model with a survival score of 5.383, a site score of 0.719, a vector score of 0.921, and a volume score (0.644) was selected as the best pharmacophore model to further steps. This model consists of 3 aromatic rings and 1 hydrogen acceptor and ligands that are present at radius of 2 Å (Matching Tolerance) in the selected model. 3274 ligands were selected for further steps after screening based on the pharmacophore model. Figure 4 shows the ARRR-1 model and the screened compounds.

### ADMET screening

At this step, LogS, Pgp-Substrate, HIA, respiratory and carcinogenicity criteria less than 0.35, DILI value less than 0.98, H-HT value less than 0.65, hERG value less than 0.60, clearance (CL) value less than 15, and terminal half-life (T_1/2_) in the range of 0.01–0.9 were evaluated using ADMETLAB server [52] (Table 1). *hERG* gene, which codes the α-subunit of potassium ion channel protein, is involved in the repolarization of the heart [53]. Therefore, the compounds that induce *hERG* gene were excluded. In comparison with approved drugs, ZINC08425746, ZINC00622382, ZINC00872960, ZINC00872940, ZINC08829718, NSC120289, ZINC00710252, ZINC00668789, and ZINC00692629 had a low ratio values for mentioned factors, which indicates a lower possibility for side effects. Among mentioned compounds, ZINC00622382, ZINC00872960, and ZINC00872940 NSC120289, as well as ZINC08433190 had also low value for DILI factor, which may reduce liver complications. Except for NSC58904, ZINC08425746, ZINC216633, ZINC00669405 and NSC55691 compounds that had a lower oral absorption rate than approved drugs, other compounds had a better oral absorption rate, which confirms the high potential of these ligands for oral route administration. Since the interaction between glycoprotein P (P-gp) and its substrates can cause them to leave the intracellular space, the lack of this kind of interaction can be a functional strategy to increase the intracellular concentration of the drug in the body [54]. Therefore, the substrates for P-gp including ZINC00702428, ZINC00622382, ZINC00872960, ZINC00625888 and ZINC00729387 were excluded from the set. In the case of solubility, just two compounds (ZINC00872960 and ZINC00710252) had an unacceptable solubility. It should be noted that the clearance of a drug is a proportionality factor that relates the concentration of drug measured in the body to the rate of elimination [55] and T_1/2_ refers to the time required for plasma concentration of a drug to decrease by 50% [56]. When the value for clearance is high, the drug is removed rapidly from the body which can be useful in reducing the side effects and toxicity of the drug, whereas a low clearance value indicates a slower removal of drug from the body which can increase the effectiveness of the drug in the body [57]. A high T_1/2_ value refers to more distribution of drug in the body [58]. Among screened compounds, NSC58904, ZINC00702428, ZINC08425746, ZINC00622382, ZINC00872960, ZINC00651156, ZINC00625888, and NSC216633 had a longer terminal half-life than the approved drugs.

**Table 1:** Evaluation of pharmacokinetic properties of approved drugs and screened molecules using ADME and ADMETLAB

No	Molecule ID	LogS	Pgp-sub	HIA	CL	T1:2	hERG	H-HT	DILI	Carcinogenicity	Respiratory Toxicity
1	Ponatinib	MdS	No	High	10.452	0.014	0.938	0.903	0.964	0.193	0.965
2	Pemigatinib	S	Yes	High	7.267	0.324	0.782	0.76	0.957	0.139	0.978
3	Infigratinib	MdS	No	High	4.039	0.026	0.935	0.651	0.986	0.078	0.694
4	Nintedanib	Mds	Yes	High	7.349	0.227	0.944	0.831	0.978	0.061	0.988
5	NSC58904	S	No	Low	10.952	0.873	0.003	0.025	0.965	0.333	0.266
6	ZINC00702428	MdS	Yes	High	8.462	0.749	0.54	0.069	0.97	0.145	0.056
7	ZINC08425746	MdS	No	Low	7.171	0.695	0.616	0.54	0.979	0.048	0.055
8	ZINC00622382	MdS	Yes	High	7.097	0.631	0.443	0.072	0.859	0.081	0.034
9	ZINC00872960	PS	Yes	High	8.085	0.59	0.001	0.303	0.855	0.013	0.098
10	ZINC00651156	MdS	No	High	7.65	0.559	0.245	0.424	0.965	0.31	0.103
11	ZINC00625888	MdS	Yes	High	2.234	0.422	0.086	0.138	0.978	0.272	0.138
12	NSC216633	MdS	No	Low	3.426	0.35	0.021	0.285	0.979	0.108	0.168
13	ZINC09045651	MdS	No	High	8.395	0.336	0.455	0.089	0.973	0.356	0.156
14	ZINC08433190	MdS	No	High	3.474	0.303	0.549	0.046	0.829	0.218	0.015
15	ZINC00872940	MdS	No	High	6.644	0.213	0.003	0.365	0.878	0.012	0.087
16	ZINC08829718	MdS	No	High	1.032	0.187	0.019	0.122	0.977	0.08	0.088
17	ZINC00659595	MdS	No	High	5.179	0.174	0.372	0.326	0.947	0.254	0.06
18	ZINC00729391	MdS	No	High	8.005	0.16	0.094	0.147	0.958	0.301	0.021
19	ZINC00729387	MdS	Yes	High	9.598	0.145	0.055	0.151	0.979	0.336	0.02
20	NSC120289	MdS	No	High	5.345	0.138	0.044	0.055	0.899	0.025	0.343
21	ZINC00702764	MdS	No	High	4.29	0.06	0.143	0.073	0.939	0.343	0.055
22	ZINC00669405	MdS	No	Low	5.575	0.054	0.12	0.475	0.952	0.247	0.301
23	ZINC00710252	PS	No	High	4.831	0.04	0.139	0.333	0.969	0.045	0.04
24	ZINC00668789	MdS	No	High	3.409	0.027	0.143	0.404	0.963	0.038	0.098
25	NSC55691	MdS	No	Low	1.765	0.012	0.085	0.154	0.963	0.146	0.023
26	ZINC00692629	MdS	No	High	3.907	0.297	0.163	0.559	0.98	0.071	0.019

S: Soluble (MdS: Moderately Soluble, Poorly Soluble), Pgp-sub: P Glycoprotein-Substrate, HIA: Human intestinal absorption, CL: Clearance, T1:2: Half-life, H-HT: Human Hepatotoxicity, DILI: Drug induced liver injury

Since prediction of toxicity is an important step in drug discovery [59], the toxicity of screened compounds was also evaluated using Protox-II server. ProTox-II currently includes methods for prediction of various toxicological endpoints such as cytotoxicity, mutagenicity, carcinogenicity, immunotoxicity, and LD50 [60] (Table 2). The LD50 is the median lethal dose meaning the dose at which 50% of test subjects die upon exposure to a compound [61]. Toxicity levels are including class I: fatal if swallowed (LD50 ≤ 5), class II: fatal if swallowed (5 < LD50 ≤ 50), class III: toxic if swallowed (50 < LD50 ≤ 300), class IV: harmful if swallowed (300 < LD50 ≤ 2000) class V: may be harmful if swallowed (2000 < LD50 ≤ 5000), and Class VI: non-toxic (LD50 > 5000) [62]. Since a high inactive toxicity value indicates a higher level of safety, it can be concluded that ZINC08425746, ZINC00622382, ZINC00625888, and ZINC08433190 are best in term of safety. In addition, ZINC00702428, ZINC00651156, NSC216633, ZINC09045651, ZINC00872940, and ZINC08829718 had only one active factor in term of toxicity. It should be noted that although some of the studied compounds have lower LD50 levels than the approved drugs, they had an acceptable toxicity class which selected for further steps.

**Table 2:** Evaluation of toxicity of approved drugs and screened molecules using Protox-II server

No	IDs	Class of Toxicity	Predicted LD50(mg/kg)	Hepatotoxicity	Carcinogenicity	Immunotoxicity	Mutagenicity	Cytotoxicity
1	Infigratinib	5	5000	Inactive	Inactive	Active	Inactive	Inactive
2	Nintedanib	4	500	Inactive	Active	Inactive	Inactive	Inactive
3	Ponatinib	5	5000	Inactive	Active	Active	Inactive	Inactive
4	Pemigatinib	4	840	Inactive	Inactive	Active	Inactive	Active
5	NSC58904	4	1000	Active	Active	Inactive	Active	Inactive
6	ZINC00702428	4	2000	Inactive	Inactive	Active	Inactive	Inactive
7	ZINC08425746	5	5000	Inactive	Inactive	Inactive	Inactive	Inactive
8	ZINC00622382	5	3000	Inactive	Inactive	Inactive	Inactive	Inactive
9	ZINC00872960	4	1460	Active	Active	Inactive	Inactive	Inactive
10	ZINC00651156	4	1700	Inactive	Inactive	Inactive	Active	Inactive
11	ZINC00625888	4	2000	Inactive	Inactive	Inactive	Inactive	Inactive
12	NSC216633	5	2300	Active	Inactive	Inactive	Inactive	Inactive
13	ZINC09045651	4	2000	Inactive	Inactive	Active	Inactive	Inactive
14	ZINC08433190	4	1000	Inactive	Inactive	Inactive	Inactive	Inactive
15	ZINC00872940	5	3040	Active	Inactive	Inactive	Inactive	Inactive
16	ZINC08829718	4	1250	Active	Inactive	Inactive	Inactive	Inactive
17	ZINC00659595	4	1200	Active	Active	Inactive	Inactive	Inactive
18	ZINC00729391	4	1500	Active	Inactive	Inactive	Inactive	Active
19	ZINC00729387	4	1500	Active	Inactive	Inactive	Inactive	Active
20	NSC120289	4	1000	Active	Inactive	Inactive	Inactive	Inactive
21	ZINC00702764	4	2000	Inactive	Inactive	Inactive	Inactive	Inactive
22	ZINC00669405	4	1500	Active	Inactive	Inactive	Active	Active
23	ZINC00710252	4	1500	Inactive	Inactive	Inactive	Inactive	Inactive
24	ZINC00668789	4	1500	Active	Inactive	Inactive	Inactive	Inactive
25	NSC55691	3	300	Active	Inactive	Inactive	Active	Inactive
26	ZINC00692629	4	1600	Inactive	Active	Active	Inactive	Active

### QSAR modeling and screening

**Fig. 5:**
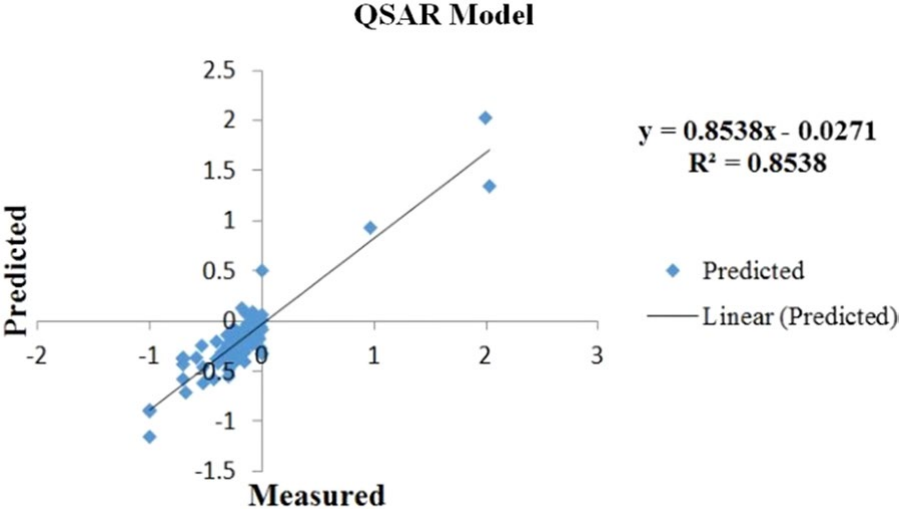
Developed QSAR model. R^2^ = 0.8538

As seen in the plot of QSAR model, the horizontal- and the vertical-axis is the measured IC_50_ and the predicted IC_50_, respectively (Fig. 5). The degree of correlation between the predicted IC_50_ and the measured IC_50_ is determined using a linear regression. The equation was y = 0.8538x–0.0271 with an acceptable R^2^ value of 0.8538. Among screened compounds, molecules with a pIC50 > 0 were selected (926 compounds). By comparing molecules obtained by the QSAR model with the screened molecules based on pharmacophore, pharmacokinetics, and toxicity (14 compounds), 6 compounds were selected for further evaluation (Table 3). Surprisingly, ZINC09045651 and ZINC00702764 had the highest pIC50 value, even more than the approved drugs.

**Table 3:** Results of QSAR overlap and molecules obtained through ADME and ADMETLAB with IC50 of confirmatory drugs

No	Molecule ID	pIC_50_ (nM)
1	Ponatininb	− 0.97313
2	Pemigatinib	− 1.99957
3	Infigratinib	0.154902
4	Nintedanib	− 2.03342
5	ZINC09045651	0.72049
6	ZINC08433190	0.51368
7	NSC120289	0.08396
8	ZINC00702764	0.55542
9	ZINC00710252	0.15670
10	ZINC00668789	0.38053

### Molecular docking and virtual screening

**Fig. 6:**
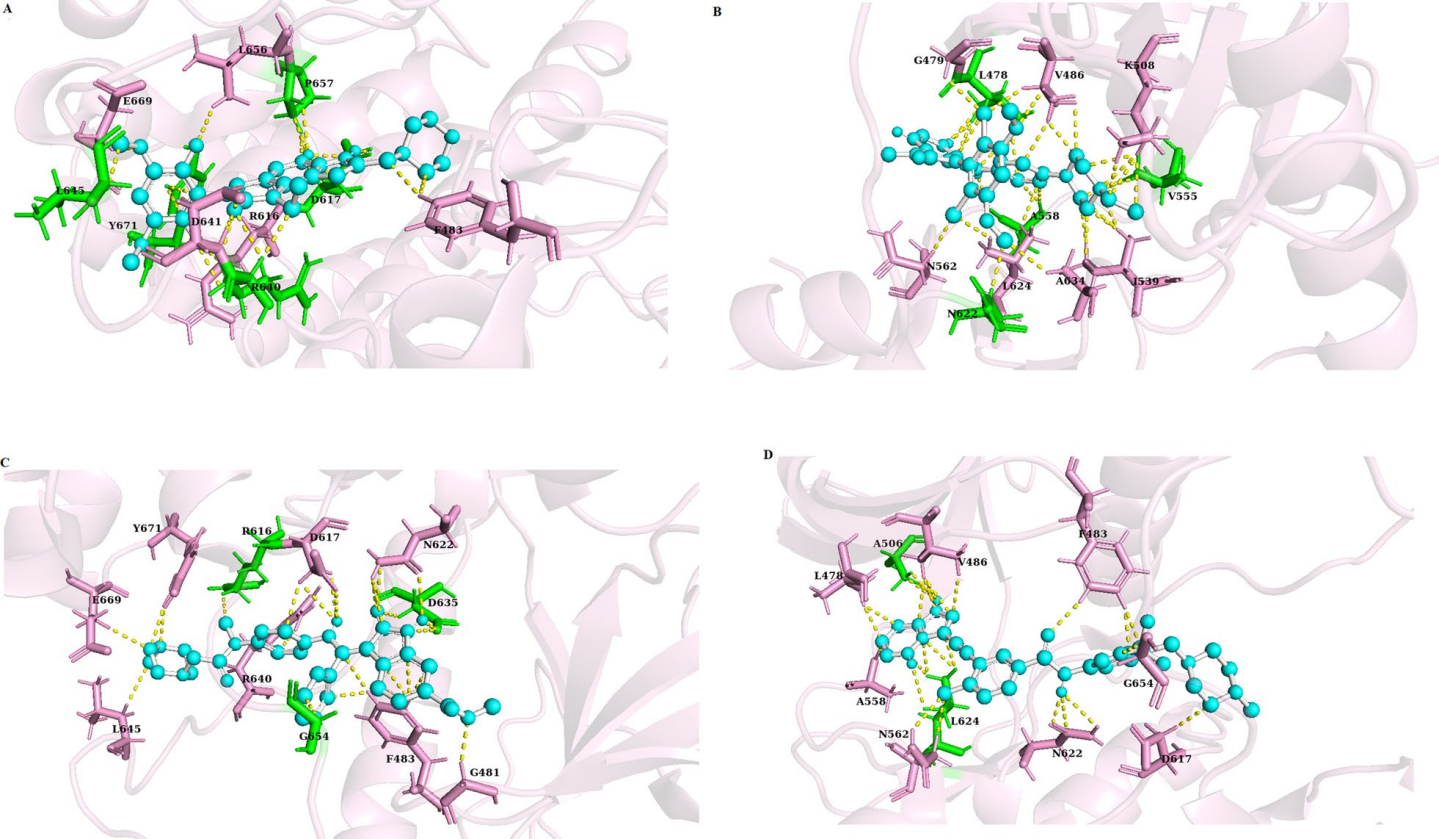
Three-dimensional (3D) structure related to the interaction between FGFR3 protein and approved drugs. **A** Pemigatinib. **B** Infigratinib. **C** Nintedanib. **D** Ponatinib. Yellow color indicates molecular bonds (hydrogen and hydrophobic), blue color indicates approved drugs and green color was used to improve differentiate of molecular bonds

**Fig. 7:**
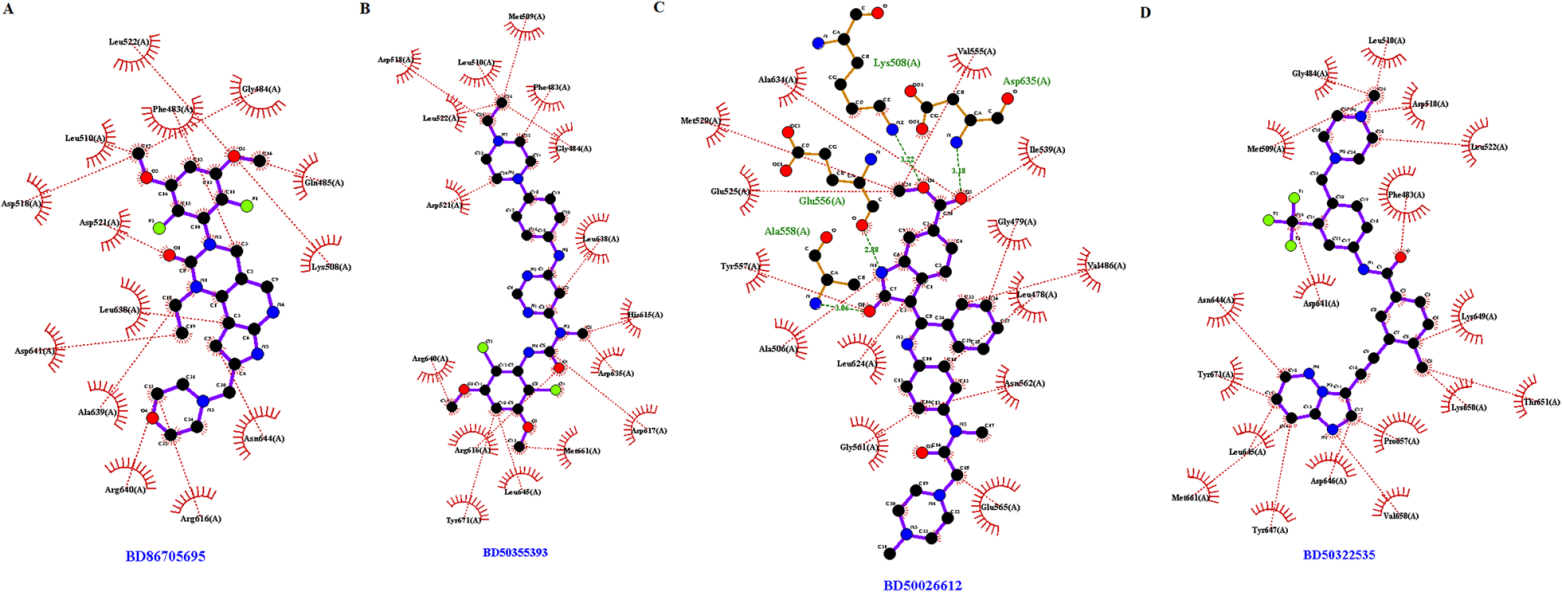
Two-dimensional (2D) structure related to the interaction between FGFR3 protein and approved drugs. **A** Pemigatinib. **B** Infigratinib. **C** Nintedanib. **D** Ponatinib, Red dotted lines indicate hydrophobic bonds and hydrogen bonding shown by green dotted lines

**Fig. 8:**
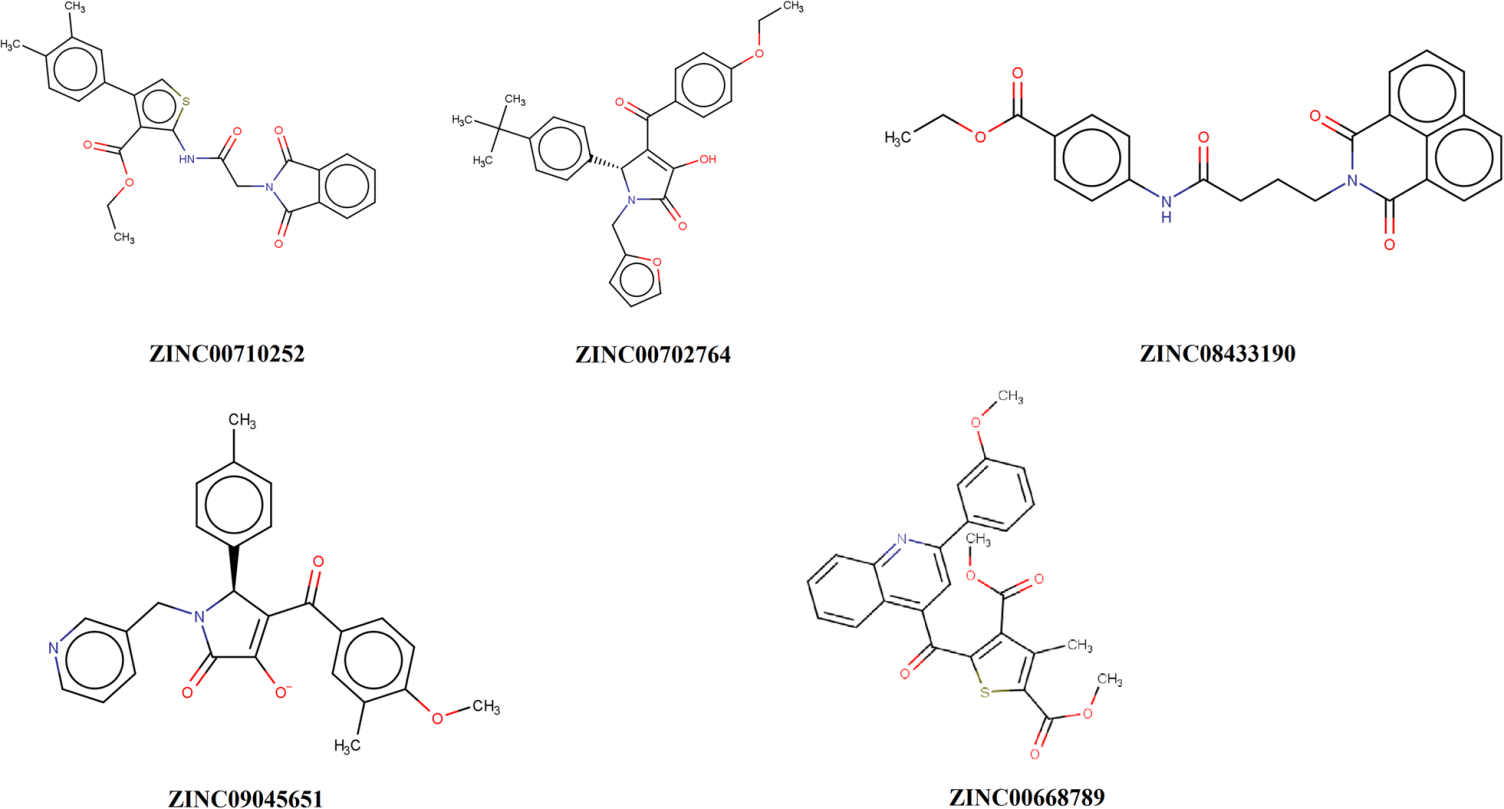
Chemical structure of candidate ligands

**Fig. 9:**
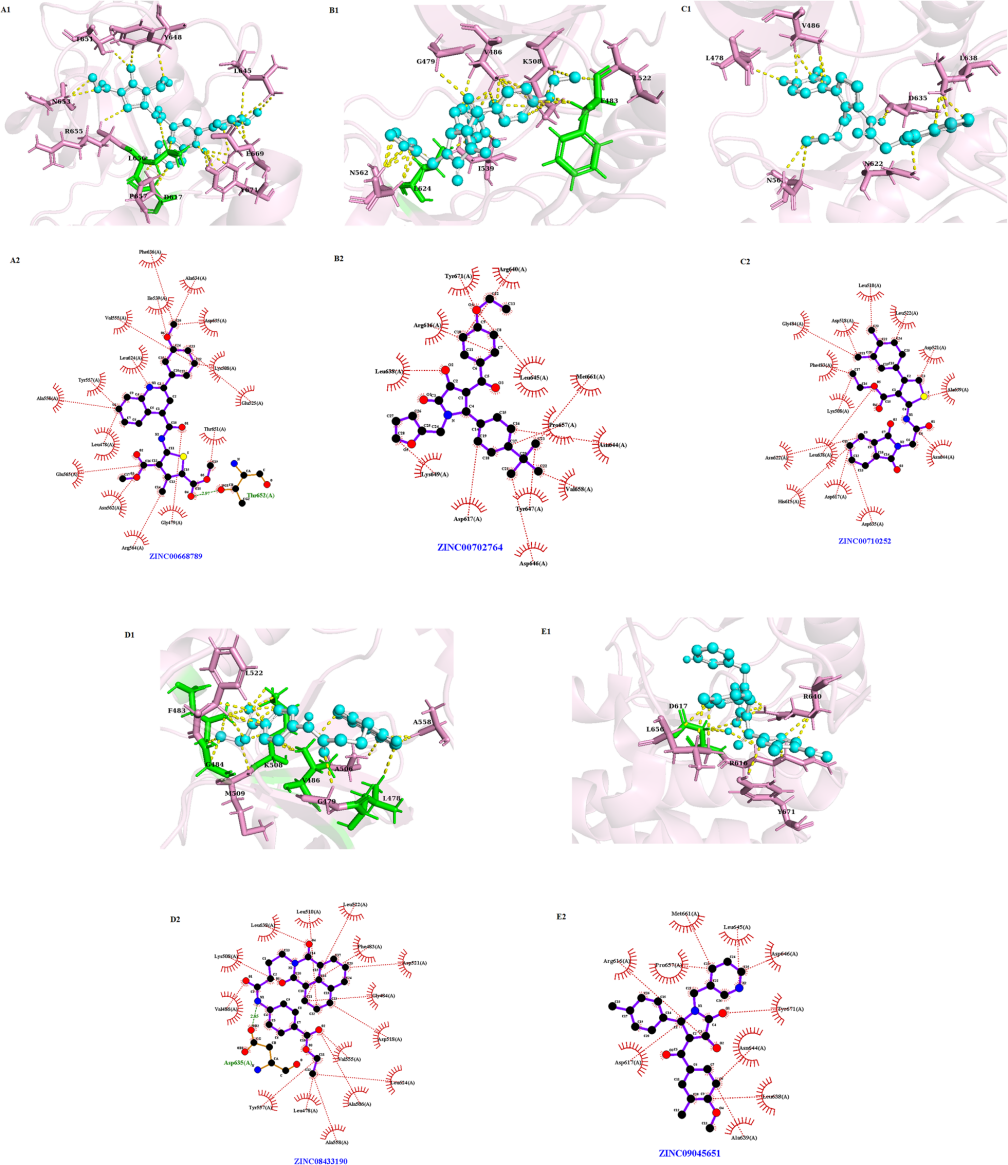
Three-dimensional (3D) structure related to the interactions of FGFR3 protein and candidate ligands at a distance of 3 Å. **A1** ZINC00668789. **B1** ZINC00702764. **C1** ZINC00710252. **D1** ZINC08433190. **E1** ZINC09045651. The two-dimensional structures are each candidate shown via **A2**, **B2**, **C2**, **D2** and **E2**, respectively

The FGFR3 binding affinity of approved drugs and the compounds with a binding affinity of less than − 8 kcal.mol^−1^ and RMSDs (lower and upper) equal to zero are displayed in Table 4. According to the results, although the screened compounds have a lower binding affinity with respect to FGFR3, they have better pharmacokinetic properties and IC_50_ value in comparison to Ponatinib. For docking analysis, all interactions between the approved drugs and FGFR3 at the active site (residues 613–625) and binding site (residues 478–508) were evaluated in 3D and 2D structures (Figs. 6 and 7, respectively). The chemical structures of finalized candidates are shown in Fig. 8 and their 3D and 2D structures of them are illustrated in Fig. 9. As mentioned before, angiogenesis in bladder cancer (Additional file 1: Fig. S1) is also mediated by EGFR and ERBB2 (Table 5). The molecular docking study between the screened compounds and these receptors revealed a high level of binding energy (< − 8 kcal.mol^−1^) for ZINC08433190, ZINC00710252 and ZINC00668789. All finalized compounds were analyzed in 3D and 2D structures which are illustrated in Additional file 1: Figs. S6 and S7.

**Table 4:** Results of molecular docking from PyRx software

No.	Molecule ID	FGFR3 protein		
		Binding affinity(kcal/mol)	Upper bind RMSD	Lower Bind RMSD
1	Ponatininb	− 10.0	0.0	0.0
2	Pemigatinib	− 8.1	0.0	0.0
3	Infigratinib	− 8.6	0.0	0.0
4	Nintedanib	− 9.1	0.0	0.0
5	ZINC09045651	− 8.3	0.0	0.0
6	ZINC08433190	− 9.2	0.0	0.0
7	ZINC00702764	− 8.2	0.0	0.0
8	ZINC00710252	− 8.3	0.0	0.0
9	ZINC00668789	− 9.1	0.0	0.0

**Table 5:** Molecular docking results of PyRx software for EGFR and ERBB2 proteins

Type of molecules	EGFR protein			ERBB2(HER-2) protein		
	Binding affinity (kcal/mol)	Upper bind RMSD	Lower bind RMSD	Binding affinity (kcal/mol)	Upper bind RMSD	Lower bind RMSD
ZINC09045651	− 7.4	0.0	0.0	− 7.8	0.0	0.0
ZINC08433190	− 9.4	0.0	0.0	− 9.5	0.0	0.0
ZINC00702764	− 7.3	0.0	0.0	− 7.2	0.0	0.0
ZINC00710252	− 9.0	0.0	0.0	− 8.9	0.0	0.0
ZINC00668789	− 8.6	0.0	0.0	− 8.3	0.0	0.0

### The interaction stability evaluation and free energy calculation after MD

**Fig. 10:**
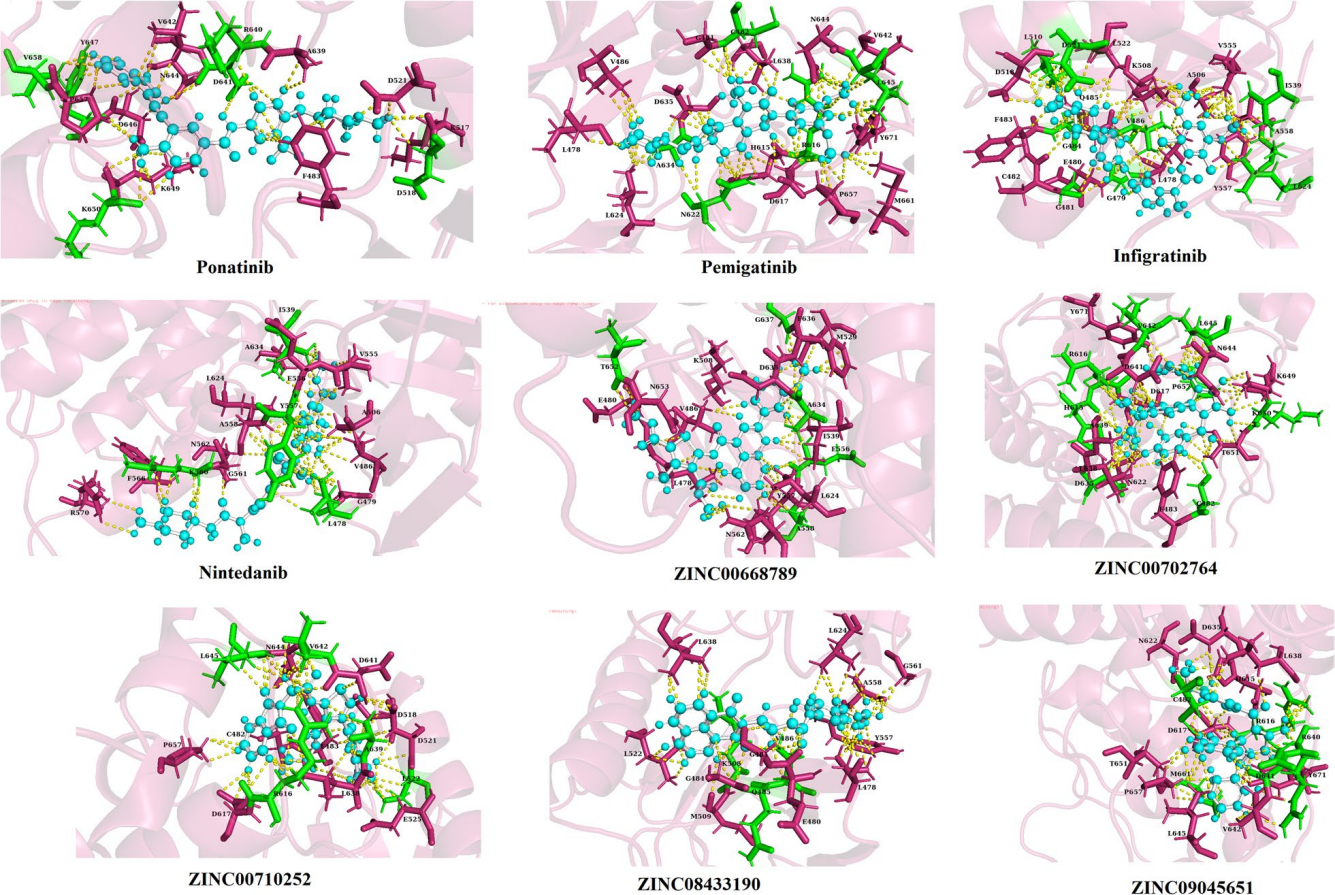
Three-dimensional structure (3D) related to the interaction between FGFR3 protein and approved drugs and candidate ligands after conducting molecular dynamics. Yellow color indicates molecular bonds (hydrogen and hydrophobic), blue color indicates approved drugs and green color was used to improve differentiate of molecular bonds

**Fig. 11:**
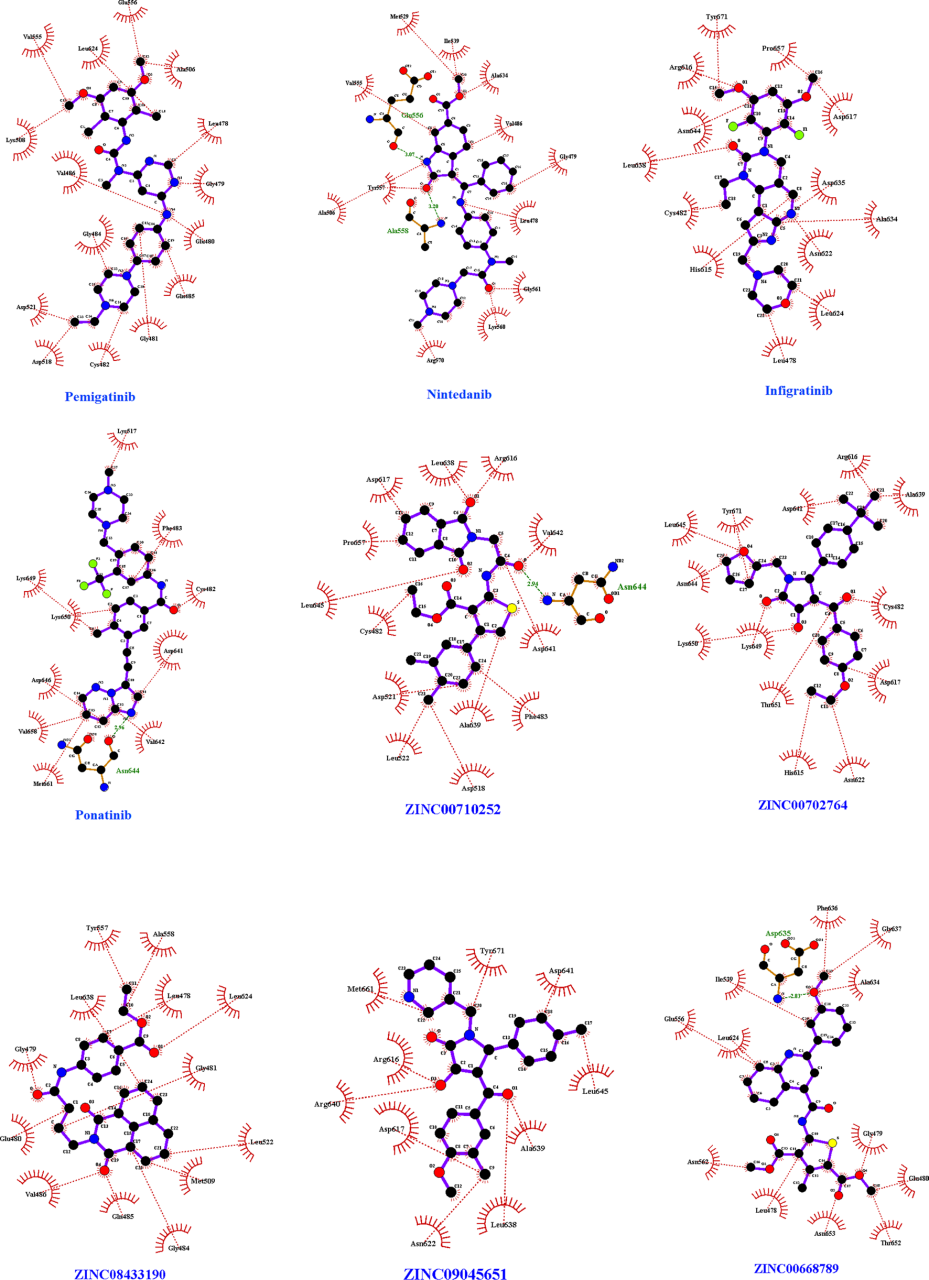
Two-dimensional (2D) structure related to the interaction between FGFR3 protein and approved drugs after conducting molecular dynamics. Red dotted lines indicate hydrophobic bonds and hydrogen bonding shown by green dotted lines

**Fig. 12:**
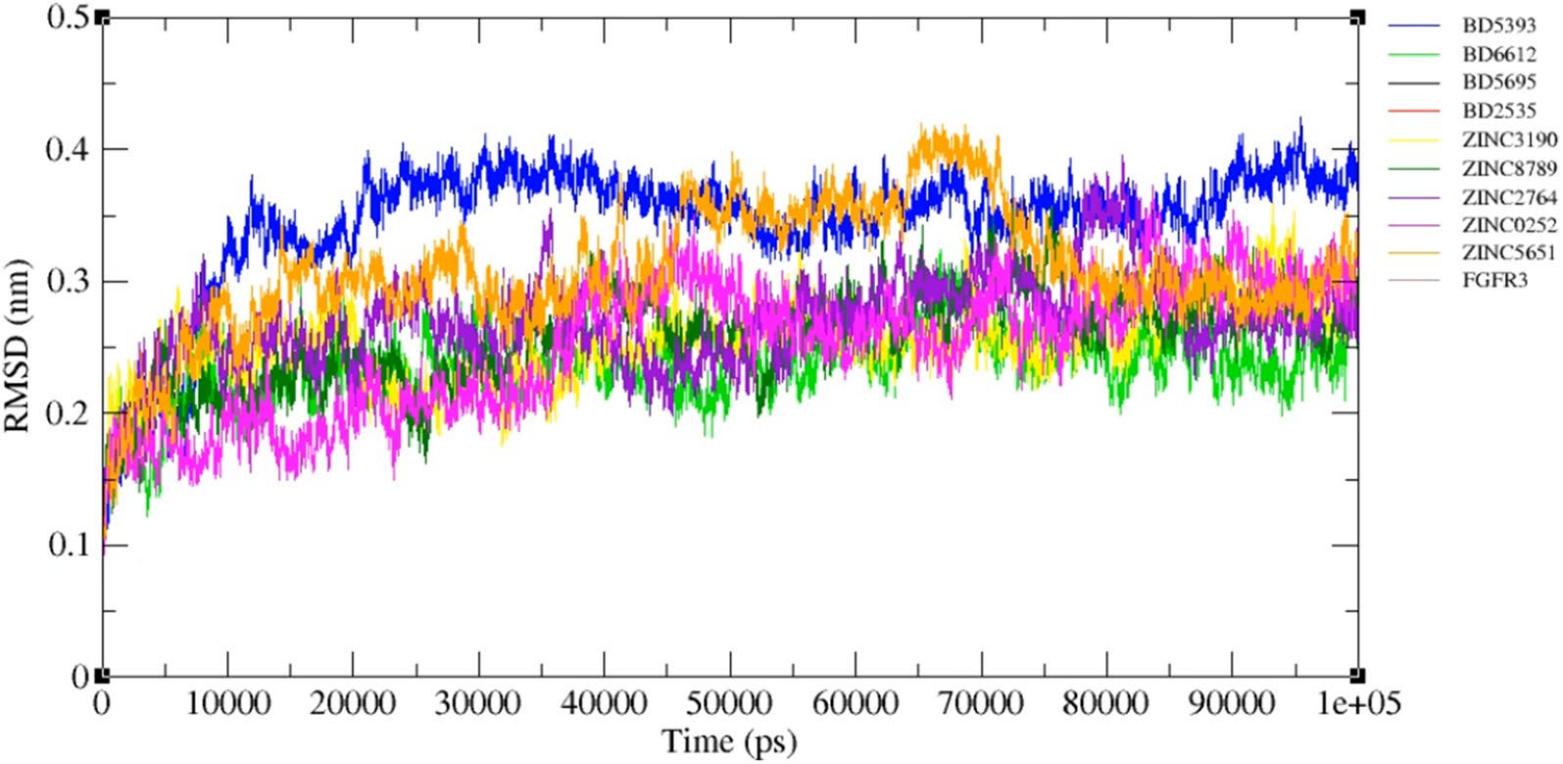
Root mean square deviation (RMSD) of free FGFR3 and FGFR3-ligand complexes during the 100 ns simulation

MD was employed to check the stability of docked structures (approved drugs and final compounds) as well as the interaction type which are illustrated in 3D and 2D structures (Figs. 10 and 11). The binding values (delta G) of screened compounds and drugs complexes with FGFR3 were also compared before and after simulation using Prodigy (Table 6). RMSD analysis revealed all screened compounds, except ZINC0252 and ZINC5651, have large fluctuations like BD6612, BD5695, and BD2535 at the beginning of simulations (0–15 ns) (Fig. 12). Thereafter (the time period of 15–100 ns), the RMSD values of all systems reached a plateau which implied the stability of systems. ZINC5651 reached a plateau at 15 ns, fluctuated between 45–70 ns, and returns to a plateau at 70 ns. Unlike other compounds, ZINC0252 reached equilibrium at initial 15 ns. The most root mean square deviation was for the BD5393-FGFR3 complex. This deviation is due to a significant increase in the number of interactions that occurred in the binding site of protein (from 2 interactions before MD to 10 interactions after MD). Moreover, although a minor deviation (in a scale of ~ 0.1 nm) has occurred for the ZINC5651-FGFR3 complex in the time range of 45–70 ns, but these deviations are under 0.2 nm, and as seen in the figure, the complex has reached the previous equilibrium state (an RMSD of 0.3 nm) with further simulation. Moreover, the free protein did not show a significant conformational change in comparison to the complexes. Therefore, it can be concluded that the binding of compounds did not cause a spatial change in the protein structure.

**Table 6:** Amino acids which involved in interactions between FGFR3 protein and candidate ligands before and after molecular dynamics using Ligplot

Type of molecules		Before MD				After MD			
		DG	H-bonds (length in Å)	Hydrophobic bonds	Active site interaction	DG	H-bonds (length in Å)	Hydrophobic bonds	Active site interaction
Drug	Ponatininb	− 5.20	–	Leu^510^,Asp^518^,Leu^522^,Phe^483^,Lys^649^,Thr^651^,Lys^650^,Pro^657^,Val^658^,Asp^646^,Tyr^647^,Met^661^,Leu^645^,Tyr^671^,Asn^644^,Asp^641^,Met^509^,Gly^484^	^−^	− 8.97	Asn^644^(2.96)	Lys^517^,Phe^483^,Cys^482^,Asp^641^,Val^642^,Met^661^,Val^658^,Asp^646^,Lys^650^,Lys^649^	^−^
	Pemigatinib	− 5.18	–	Leu^522^, Phe^483^, Leu^510^, Gly^484^, Asp^518^, Asp^521^, Leu^638^, Asp^641^, Ala^639^, Arg^640^, Arg^616^, Asn^644^, Lys^508^, Gln^485^	Asp^617^,Leu^624^,His^615^,Arg^616^	− 9.35	–	Pro^657^,Asp^617^,Asp^635^,Ala^634^,Asn^622^,Leu^624^,Leu^478^,His^615^,Cys^482^,Leu^638^,Asn^644^,Arg^616^,Tyr^671^	Asp^617^,Leu^624^,His^615^,Arg^616^
	Infigratinib	− 5.15	–	Met^509^, Leu^510^, Phe^483^, Asp^518^, Leu^522^, Gly^484^, Asp^521^, Leu^638^, His^615^, Asp^635^, Asp^617^, Met^661^, Leu^645^, Tyr^671^, Arg^616^, Arg^640^	Leu^624^	− 8.32	–	Glu^556^,Ala^506^,Leu^478^,Gly^479^,Glu^480^,Glu^485^,Gly^481^,Cys^482^,Asp^518^,Asp^521^,Gly^484^,Val^486^,Lys^508^,Val^555^,Leu^624^	Leu^624^
	Nintedanib	− 5.21	Asp^635^(3.18), Lys^508^(3.22), Glu^556^(2.88), Ala^558^(3.06)	Val^555^, Ile^539^, Gly^479^, Leu^478^, Val^486^, Asn^562^,Glu^565^, Gly^561^, Leu^624^, Ala^506^, Tyr^557^, Glu^525^, Met^529^, Ala^634^	^−^	− 9.82	Ala^558^(3.20),Glu^556^(3.07)	Met^529^,Ile^539^,Ala^634^,Val^486^,Gly^479^,Leu^478^,Gly^561^,Lys^560^,Arg^570^,Ala^506^,Tyr^557^,Val^555^	^−^
Final selected ligands	Zinc00668789	− 5.11	Thr^652^(2.97)	Phe^636^, Ala^634^, Ile^539^, Asp^635^, Val^555^, Leu^624^, Tyr^557^, Ala^558^, Leu^478^, Glu^565^, Asn^562^, Arg^564^, Gly^479^, Thr^651^, Glu^525^, Lys^508^	Leu^624^	− 10.26	Asp^635^(2.83)	Phe^636^,Gly^637^,Ala^634^,Gly^479^,Glu^480^,Thr^652^,Asn^653^,Leu^478^,Asn^562^,Leu^624^,Glu^556^,Ile^539^	Leu^624^
	Zinc00702764	− 5.09	–	Tyr^671^, Arg^640^, Arg^616^, Leu^638^, Lys^649^, Asp^617^, Asp^646^, Tyr^647^, Val^658^, Asn^644^, Pro^657^, Met^661^, Leu^645^	Asp^617^,Asn^622^,His^615^,Arg^616^	-10.69	–	Ala^639^,Cys^482^,Asp^617^,Asn^622^,His^615^,Thr^651^,Lys^649^,Lys^650^,Asn^644^,Leu^645^,Tyr^671^,Asp^641^,Arg^616^	Asp^617^,Asn^622^,His^615^,Arg^616^
	Zinc00710252	− 5.06	–	Leu^510^, Asp^518^, Gly^484^, Phe^483^, Lys^508^, Leu^638^, Asn^622^, His^615^, Asp^617^, Asp^635^, Asn^644^, Ala^639^, Asp^521^, Leu^522^	Asp^617^,Arg^616^	− 10.53	Asn^644^(2.94)	Val^642^,Asp^641^,Phe^483^,Ala^639^,Asp^518^,Leu^522^,Asp^521^,Cys^482^,Leu^645^,Pro^657^,Asp^617^,Leu^638^,Arg^616^	Asp^617^,Arg^616^
	Zinc08433190	− 5.03	Asp^635^(2.85)	Leu^522^, Leu^510^, Leu^638^, Lys^508^, Val^486^, Tyr^557^, Leu^478^, Ala^558^, Ala^506^, Leu^624^,Val^555^, Asp^518^, Gly^484^, Asp^521^, Phe^483^	Leu^624^	− 9.69	–	Ala^558^,Leu^478^,Leu^624^,Gly^481^,Leu^522^,Met^509^,Gly^484^,Gln^485^,Val^486^,Glu^480^,Gly^479^,Leu^638^,Tyr^557^	Leu^624^
	Zinc09045651	− 5.15	–	Met^661^, Pro^657^, Arg^616^, Asp^617^, Ala^639^, Leu^638^, Asn^644^, Tyr^671^, Asp^646^, Leu^645^	Asp^617^,Asn^622^,Arg^616^	− 10.04	–	Asp^641^,Leu^645^,Ala^639^,Leu^638^,Asn^622^,Asp^617^,Arg^640^,Arg^616^,Met^661^,Tyr^671^	Asp^617^,Asn^622^,Arg^616^

**Fig. 13:**
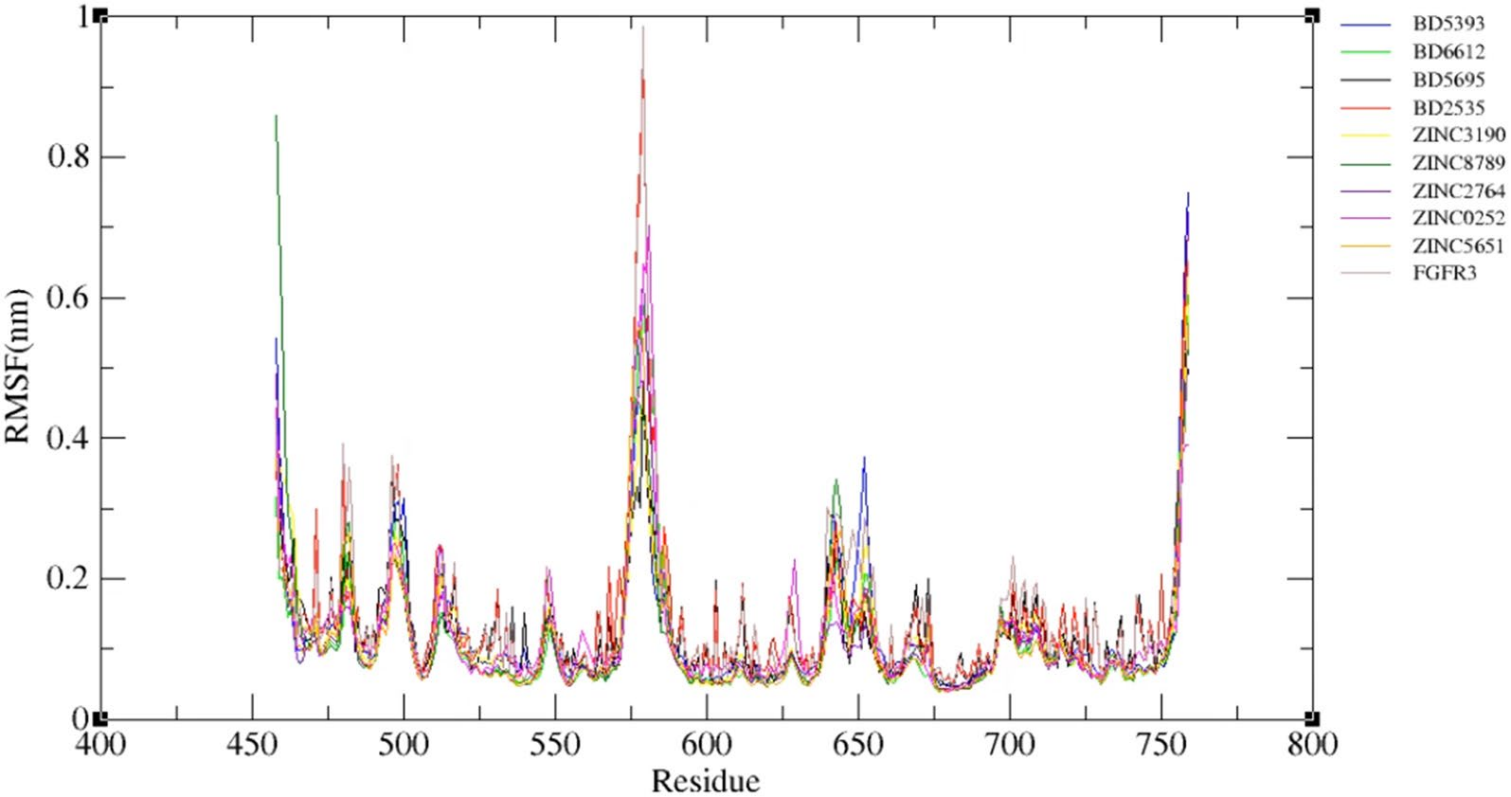
Root mean square fluctuation (RMSF) value per residue of free FGFR3 and FGFR3-ligands complexes during the 100 ns simulation

The RMSF of backbone atoms was calculated during 100 ns in each system to examine the flexibility of complexes. A higher RMSF value indicates a more flexible residue, whereas the lower one, a more stable residue. Generally, loop regions and the N-terminus and C-terminus of a protein display more flexibility throughout the MD simulation than secondary structures and residues involved in inter- and intramolecular interactions. As shown in Fig. 13, although the screened compounds had a more fluctuation in the C- and N-terminus regions, however, the fluctuations of these complexes and the approved drugs (BD2535, BD5695, BD6612, and BD5393) was approximately equal in compared to the free protein at the active site (residues 613–625).

**Fig. 14:**
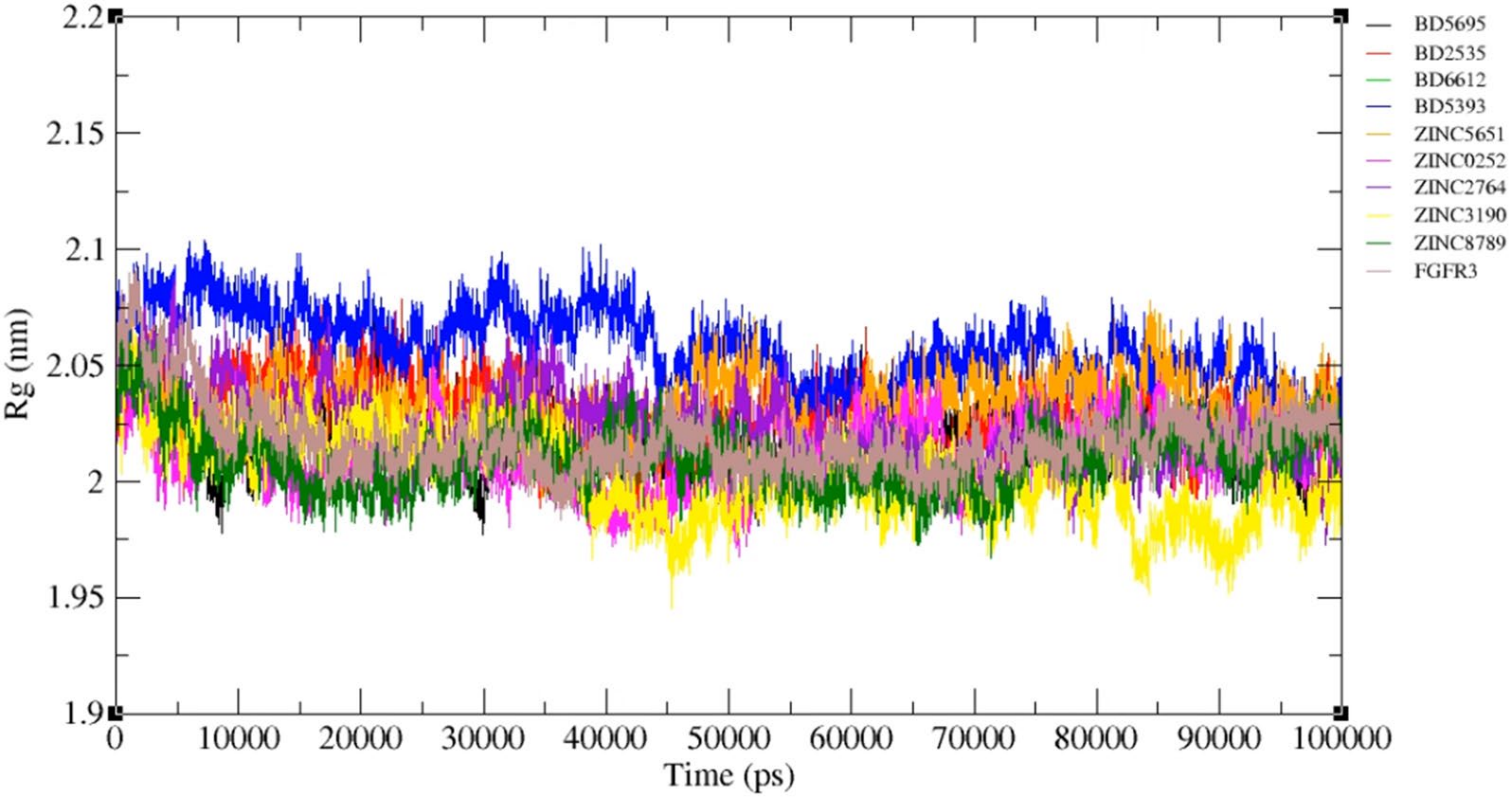
Radius of gyration (Rg) value of free FGFR3 and FGFR3-ligands complexes during the 100 ns simulation

The radius of gyration (Rg) for free FGFR3 and the complexes was calculated. The Rg value provides the level of protein compactness. A higher value means a lower compactness in the protein [63]. As shown in Fig. 14, all proteins had a same Rg value.

**Fig. 15:**
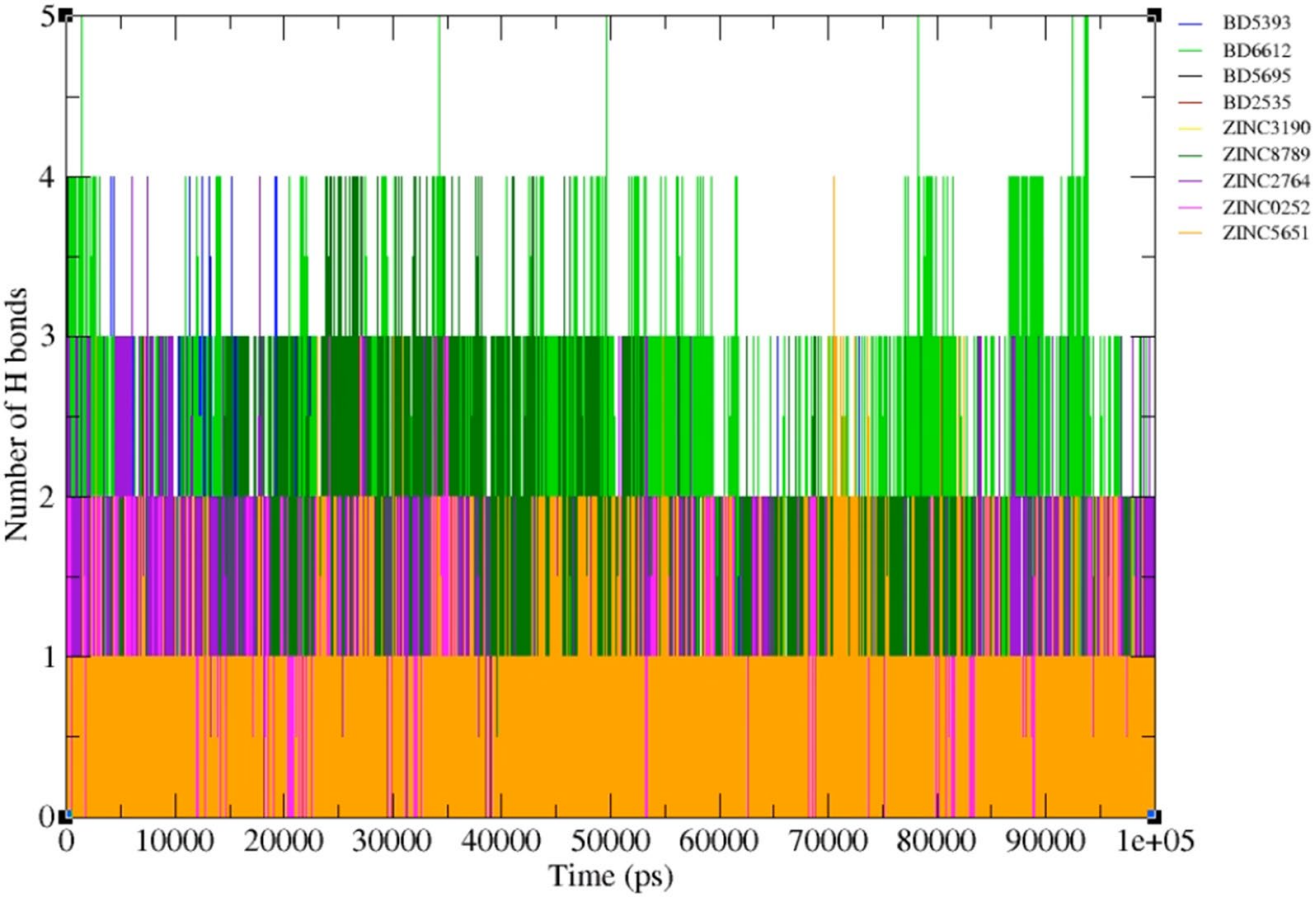
Numbers of intermolecular hydrogen bonds in FGFR3-ligands complexes during the 100 ns simulation

H-bonds play a vital role in forming strong bonds between the ligands and receptor [64]. As shown in Fig. 15, all compounds have at least two hydrogen bonds in the majority of the time. Among approved drugs, BD6612 had formed the maximum number of hydrogen bonds (5 bonds) within the simulation time. Among screened compounds, ZINC8789 and ZINC2764 formed H-bonds with the active site indicating these compounds had a more capacity for H-bond forming in comparison to BD5393, BD2535, and BD5695.

**Fig. 16.**
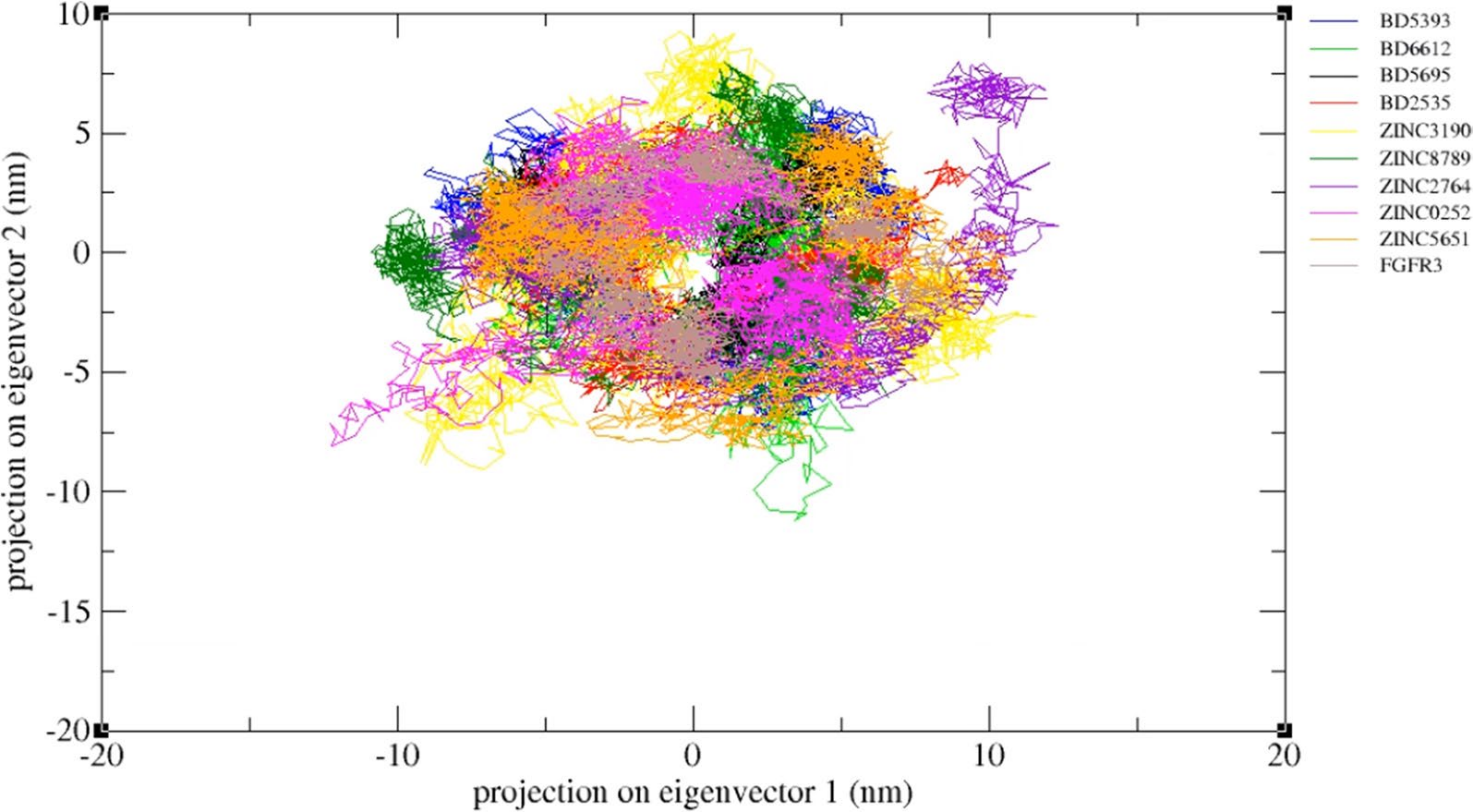
2D projections of the nine complexes on eigenvector 1 and eigenvector 2

Structural changes and molecular motions in the protein domains were evaluated using PCA method, regardless of its intrinsic fluctuations [65]. The distribution of dots on the plot shows the level of motion in the protein structure (Fig. 16). More scattered dots imply more flexibility and motions in a complex, and less scattered dots indicate a lower level of motions and hence a more inhibitory effect of the ligand on the protein [66]. The higher density and less dispersion of ZINC0252 and ZINC5651 in comparison to BD5393 and BD2535 display the stability, hardness, and high inhibitory potency of these compounds in complex with FGFR3 protein.

Among approved drugs, the binding values for Infigratinib and Nintedanib decreased, while the binding value increased for Pemigatinib and remained unchanged for Ponatinib. In contrast, all binding values became more negative after the MD simulations, which indicated the stability of interactions between the screened compounds and FGFR3. It must be notified that the screened compounds showed a higher reduction in the delta G than the approved drugs.

According to Table 6, all drugs had hydrophobic interactions, although the number of these bonds decreased slightly after MD. Ponatinib had no hydrogen bond in the active site of FGFR3 before MD, whereas after simulation a hydrogen bond was formed between nitrogen atom and residue Asn644. Pemigatinib had no hydrogen bond and a hydrophobic bond (Arg616) in the active site before MD, but after MD the number of bonds increased to four (Asp617, Leu624, His615, Arg616). Infigratinib formed three bonds in the active site (His615, Asp617 and Arg616) before MD. However, the number of bonds was reduced to one after MD simulation (Leu624). Nintedanib had four hydrogen bonds (Asp635, Lys508, Glu556, and Ala558) and a hydrophobic bond (Leu624) with the active site before MD, which reduced after MD to two (Ala558, Glu556) and zero bonds, respectively.

ZINC00710252 showed a decrease in the number of bonds for the active site. ZINC00668789 and ZINC08433190 exhibited an equal number of bonds before and after MD. However, ZINC00702764 and ZINC09045651 displayed an increase in the number of bonds for the active site. Among the screened compounds, only ZINC00668789 (Thr652), ZINC08433190 (Asp635) had one and ZINC00710252 had no hydrogen bond before MD, while ZINC00668789 (Asp635) ZINC00710252 (Asn644) showed one and ZINC08433190 had no hydrogen bond after simulation. Before MD, ZINC00668789 (Leu624) and ZINC08433190 (Leu624) have one bond with the active site, ZINC00702764 (Arg616 and Asp617) and ZINC09645651 (Asp617 and Arg616) have two bonds and ZINC00710252 (Asn622, His615, and Asp617) has three bonds with the active site, while after MD, ZINC00668789 and ZINC08433190 maintained their bond to active site in Leu624. The number of interactions with active site in ZINC00702764 (Asp617, Asn622, His615, and Arg616) and ZINC09045651 (Asp617, Asn622, and Arg616) increased to four and three bonds after MD, in turn. Interactions with active site declined to two bonds in ZINC00710252 after simulation (Asp617 and Arg616).

### Structural characterization of screened compounds in terms of in vivo stability

According to Table 1, all screened compounds have passed the ADMET filters. But, it is very important to practically check the ester bonds (ethyl or methyl ester) in the screened compounds because these bonds can be easily degraded by esterases in the body, and if these bonds are involved in the interaction, the inhibitory effect of ligands may be affected. As seen in Table 7, there is no interaction between the ester bonds of Zinc3190 and Zinc5651 with the binding site (478–508) or active site (617–625) of FGFR3 protein. However, there are limited interactions between the ester bonds of Zinc0252 (ethyl ester bond with the binding site), Zinc2764 (ethyl ester bond with the active site), and Zinc8789 (methyl ester bond with the binding site) and the protein that can be potentially affected by esterases. Undoubtedly, the in vivo tests can be useful to determine the degradability level of ligands in the body. Nonetheless, improving stability against esterases is now simply possible through the chemical derivatization of compounds in the field of pharmaceutical chemistry.

**Table 7:** The ester bonds involved in the interaction with the active site or binding site of FGFR3 protein

Ligand ID	Ethyl ester		Methyl ester	
	Active site	Binding site	Active site	Binding site
Zinc0252	–	Cys^482^	–	–
Zinc3190	–	–	–	–
Zinc2764	His^615^ Asn^622^	–	–	–
Zinc5651	–	–	–	–
Zinc8789	–	–	–	Glu^480^

### The interaction analysis between the screened compounds and clinically important FGFR3 mutations

We used the native FGFR3 protein for the screening procedure. But as mentioned before, the clinically important mutations that naturally occurred in bladder cancer patients may reduce the efficacy of screened compounds. To check this issue, we retrieved the clinically important variants reported for bladder cancer from UniprotKB database (https://www.uniprot.org/uniprotkb/P22607) and analyzed the interaction of screened compounds with these positions. Data revealed there are five clinically important point mutations for FGFR3 including R248C, S249C, G370C, K650E, and K650Q (variant IDs of VAR_004148, VAR_004149, VAR_004151, VAR_004160, and VAR_018390, respectively). Among the approved drugs and screened compounds, only Ponatininb and Zinc2764 have interactions with the Lys 650. It must also be notified that this residue is not located in the active site (residues 613–625) and binding site (residues 478–508) of FGFR3 protein. Therefore, these mutations cannot potentially affect the efficacy of Zinc0252, Zinc3190, Zinc5651, and Zinc8789, but in vitro and in vivo experiments should be conducted in the future to reveal this issue.

### Off-targeting

Conserved domain analysis in NCBI revealed a high sequence similarity between the active site of FGFR3 protein (residues 613–625) and protein kinase C (PKC) superfamily (Additional file 1: Fig. S8A and S8B). Moreover, assessing Ponatinib and Nintedanib in STITCH server indicates a strong interaction between these drugs and kinase family including ABL1 (score 0.996), KDR (score 0.968), KIT (score 0.977), FLT3 (score 0.987) and RET (score 0.978) (Additional file 1: Fig. S8C). These proteins have various physiological roles such as wound healing, epithelial cell proliferation, regulation of colon, small intestine and stomach smooth muscle contractions, and differentiation of lymphocytes, B cells, and red blood cells [67–71]. The binding of drugs to these proteins can cause possible adverse effects such as weakening of the immune system, anemia, delay in wound healing, gastric ulcers, nausea, and vomiting [72, 73]. Therefore, the binding energy of screened compounds to the proteins was studied using molecular docking and compared to the approved drugs (Ponatinib and Nintedanib). According to Additional file 1: Table S3, the binding energy of ZINC08433190, ZINC00668789, ZINC00710252, ZINC00702764, and ZINC09045651 to ABL1, KDR, KIT, FLT3 and RET proteins was lower than Ponatinib. Also, the binding energy values for KDR, KIT, ABL1, and RET proteins were less or equal in compared to Nintedanib. It should be noted that the binding energy of ZINC00668789 on FLT3 protein was less than the binding energy of Nintedanib under similar conditions. Therefore, based on the obtained binding energy values for screenzfed compounds, the side effects of candidates can be potentially less than Ponatinib and Nintedanib. The 3D and 2D of docked structures are shown in Additional file 1: Figs. S9 and S10, respectively. Also, according to the pathology and biotech section of Uniprot server, these proteins are also involved in the development of various cancers such as leukemia, thyroid, stomach and intestine, colorectal, and breast [74]. Therefore, these proteins can be considered as targets for such cancers.

### The potential effect of screened compounds on other cancers

As stated in Additional file 1: Table S1, HRAS, KRAS, and RB1 proteins also have key roles in the development of other cancers [75–77]. Therefore, the binding ability of screened compounds to HRAS, KRAS and RB1 proteins was studied using molecular docking (Additional file 1: Table S4). The outcomes revealed the effectiveness of screened compounds in binding to HRAS, KRAS and RB1 proteins. This suggests the potential application of screened compounds in the treatment of other cancers in addition to bladder cancer. The 3D and 2D structures are shown in Additional file 1: Figs. S11 and S12, respectively.

## Conclusion

In this study, we discovered the potential FGFR3 inhibitors as anti-bladder cancer agents. At first, the top 100 molecules obtained from BindingDB and the FGFR3-approved drugs were used to generate the QSAR and pharmacophore models. Then, ZINC and NCI databases were screened by the pharmacophore models. The outputs (2743 molecules) were filtered by Swiss-ADME, ADMETLAB, and Protox-II to check the ADMET properties and the drug-like criteria. The resulted compounds (14 molecules) were screened by the QSAR model to catch the potent binding molecules. The final candidates (5 molecules) were checked by molecular docking and molecular dynamics simulation for the potential inhibitory effect. Our results indicated that ZINC3190, ZINC5651, ZINC2764, ZINC0252, and ZINC8789 could potentially inhibit FGFR3 which were comparable to the approved drugs. Moreover, the off-targeting analysis indicated that some of them exhibit lower toxicity than the approved drugs. The candidates were suggested for further in vitro and in vivo experiments to verify the potential inhibitory effect in bladder cancer therapy.

## Supplementary Information

**Additional file 1: Table S1.** Target detection for treatment of bladder cancer. **Table S2.** Analysis of target genes in bladder cancer via DisGeNET server. **Figure S1.** The effect of FGFR3 gene in bladder cancer signalling pathway. **Figure S2.** Secondary structure of FGFR3. **Figure S3.** Ramachandran plot of FGFR3. **Figure S4.** ERRAT plot of FGFR3 (overall quality factor: 96.512%). **Figure S5.** Quality factor assessment of FGFR3 using ProsAweb server. **Figure S6.** 3D docking structure of finalized ligands with EGFR (A-E) & ERBB2 (F-J). Yellow color indicates interaction (Hydrogen & hydrophobic bonds), Cyan represents ligands. The red & green color used in some pictures for distinguishing the interaction bonds. **Figure S7.** 2D docking structure of finalized ligands with EGFR & ERBB2. The green color indicates H-bonds and red color signifies hydrophobic contacts. **Figure S8.** (A): Detection of conserved domains using NCBI (B): The alignment results of FGFR3, ABL1, KDR (VGFR), KIT, FLT3, RET (red color: active site, green color: binding site). (C): The interaction of ponatinib and nintedanib with ABL1, KDR, KIT, FLT3, RET. **Table S3.** molecular docking of finalized ligands with ABL1, KDR, KIT, FLT3, RET. **Figure S9.** 3D structure of docking ABL1 (1–7), KDR (8–13), KIT (14–20), FLT3 (21–27), RET (28–34). Yellow color indicates interaction (Hydrogen & hydrophobic bonds), Cyan represents ligands. The red & green color used in some pictures for distinguishing the interaction bonds. **Figure S10.** 2D docking structure of finalized ligands and approved drugs with ABL1 (1–7), KDR (8–13), KIT (14–20), FLT3 (21–27), RET (28–34). The green color indicates H-bonds and red color signifies hydrophobic contacts. **Table S4.** molecular docking of finalized ligands with HRAS, KRAS and RB1. **Figure S11.** Prediction of docking 3D structure using Pymol software (any contacts between finalized ligands and proteins within 3 Å). HRAS (A-E), KRAS (FJ), RB1 (K–O).Yellow color indicates interaction (Hydrogen & hydrophobic bonds), Cyan represents ligands. The red color used in some pictures for distinguishing the interaction bonds. **Figure S12.** prediction of 2D docking structure with HRAS, KRAS, RB1using Ligplot software (H-bonds & hydrophobic contacts between finalized ligands and proteins by software defaults).

## Data Availability

The authors confirm that the data supporting the findings of this study are available within the article [and/or] its additional file.
