# Supplementary material for: Discovery of potential FGFR3 inhibitors via QSAR, pharmacophore modeling, virtual screening and molecular docking studies against bladder cancer
Source: J Transl Med. 2023 Feb 10;21:111. doi: 10.1186/s12967-023-03955-5 (PMC9913026; doi:10.1186/s12967-023-03955-5)
Supplement: Supplementary file 1 — Additional file 1: Table S1. Target detection for treatment of bladder cancer. Table S2. Analysis of target genes in bladder cancer via DisGeNET server. Figure S1. The effect of FGFR3 gene in bladder cancer signalling pathway. Figure S2. Secondary structure of FGFR3. Figure S3. Ramachandran plot of FGFR3. Figure S4. ERRAT plot of FGFR3 (overall quality factor: 96.512%). Figure S5. Quality factor assessment of FGFR3 using ProsAweb server. Figure S6. 3D docking structure of finalized ligands with EGFR (A-E) & ERBB2 (F-J). Yellow color indicates interaction (Hydrogen & hydrophobic bonds), Cyan represents ligands. The red & green color used in some pictures for distinguishing the interaction bonds. Figure S7. 2D docking structure of finalized ligands with EGFR & ERBB2. The green color indicates H-bonds and red color signifies hydrophobic contacts. Figure S8. (A): Detection of conserved domains using NCBI (B): The alignment results of FGFR3, ABL1, KDR (VGFR), KIT, FLT3, RET (red color: active site, green color: binding site). (C): The interaction of ponatinib and nintedanib with ABL1, KDR, KIT, FLT3, RET. Table S3. molecular docking of finalized ligands with ABL1, KDR, KIT, FLT3, RET. Figure S9. 3D structure of docking ABL1 (1–7), KDR (8–13), KIT (14–20), FLT3 (21–27), RET (28–34). Yellow color indicates interaction (Hydrogen & hydrophobic bonds), Cyan represents ligands. The red & green color used in some pictures for distinguishing the interaction bonds. Figure S10. 2D docking structure of finalized ligands and approved drugs with ABL1 (1–7), KDR (8–13), KIT (14–20), FLT3 (21–27), RET (28–34). The green color indicates H-bonds and red color signifies hydrophobic contacts. Table S4. molecular docking of finalized ligands with HRAS, KRAS and RB1. Figure S11. Prediction of docking 3D structure using Pymol software (any contacts between finalized ligands and proteins within 3 Å). HRAS (A-E), KRAS (FJ), RB1 (K–O).Yellow color indicates interaction (Hydrogen & hydrophobic bo [file 12967_2023_3955_MOESM1_ESM.pdf]

Table S1: Target detection for treatment of bladder cancer

Target Genes	Databases								
	Target Genes				Approved Drug			Other cancers	
	OMIM	KEGG	Pharos	DisGeNET	Drug Bank	Pharos	KEGG	KEGG	DisGeNET (Score$_{gda}$)
FGFR3	✓	✓	✓	✓	✓	✓	✓	Multiple myeloma	Multiple myeloma(0.700), Colorectal Carcinoma(0.660), Adenocarcinoma of lung(0.650), Cervical Cancer(0.540), Adenocarcinoma of large intestine(0.500)
HRAS	✓	✓	✓	✓	×	×	×	Cervical Cancer, PenileCancer, Thyroid Cancer,Squamous cell carcinoma, Giant Cell Tumor Bone, Merkel Cell Carcinoma, Medullary Thyroid cancer	Melanoma(0.800), Squamous cell carcinoma(0.600), Breast Carcinoma(0.600), Thyroid carcinoma(0.560), Glioma(0.520), Adenocarcinoma of lung(0.500), Leukemia(0.500), Neuroblastoma(0.600), Liver Carcinoma(0.500)
KRAS	✓	×	×	✓	✓	×	×	-	Non-Small Cell Lung Carcinoma(0.700), Juvenile Myeloma(1.00), Adenocarcinoma of lung(0.700), Carcinoma of lung(0.700), Pancreatic carcinoma(0.700), Breast Carcinoma(0.700), Leukemia(0.700), Liver carcinoma(0.700), Colorectal arcinoma(0.600), Lymphoma(0.440), Neuroblastoma(0.450), Squamous cell carcinoma of lung(0.430), Ovarian Carcinoma(0.470)
RB1	✓	×	✓	✓	×	×	×	Chronic Myeloid Leukemia, Small Cell Lung cancer Esophageal Cancer, Osteosarcoma, Glioma, Hepatocellular Carcinoma, Retinoblastoma	Small cell carcinoma of lung(0.720), Osteosarcoma(0.700), Breast Carcinoma(0.600), Liver carcinoma(0.400), Squamous cell carcinoma of esophagus(0.340), Childhood Acute Lymphoblastic Leukemia(0.320), Retinoblastoma(0.800)

*Score$_{gda}$: Gene-disease association score

Table S2: Analysis of target genes in bladder cancer via DisGeNET server

Target Genes	Accession number	Length	DSI_g	$Score_{gda}$	N.PMIDs	First Ref	Last Ref
FGFR3	P22607	806	0.391	0.500	94	2001	2019
HRAS	P01112	189	0.378	0.700	29	1983	2018
KRAS	P01116	189	0.320	0.090	9	2001	2019
RB1	P06400	928	0.444	0.310	1	2002	2002

*DSI_g: Disease specificity Index for the gene *$Score_{gda}$: Gene-disease association score *N.PMIDs: Total number of PMIDs supporting the association

*Ref: Reference

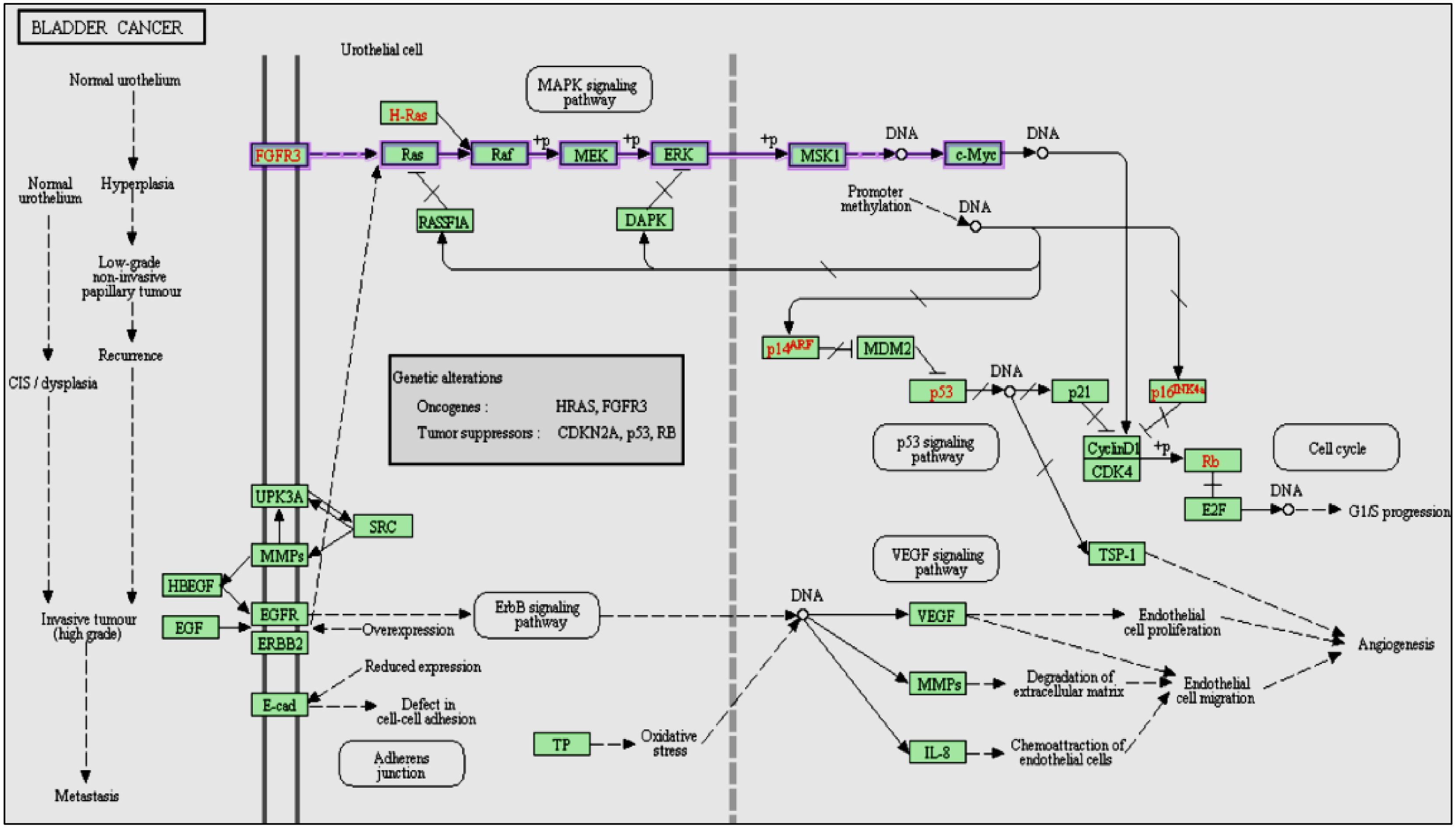

Figure S1: The effect of FGFR3 gene in bladder Cancer signaling pathway

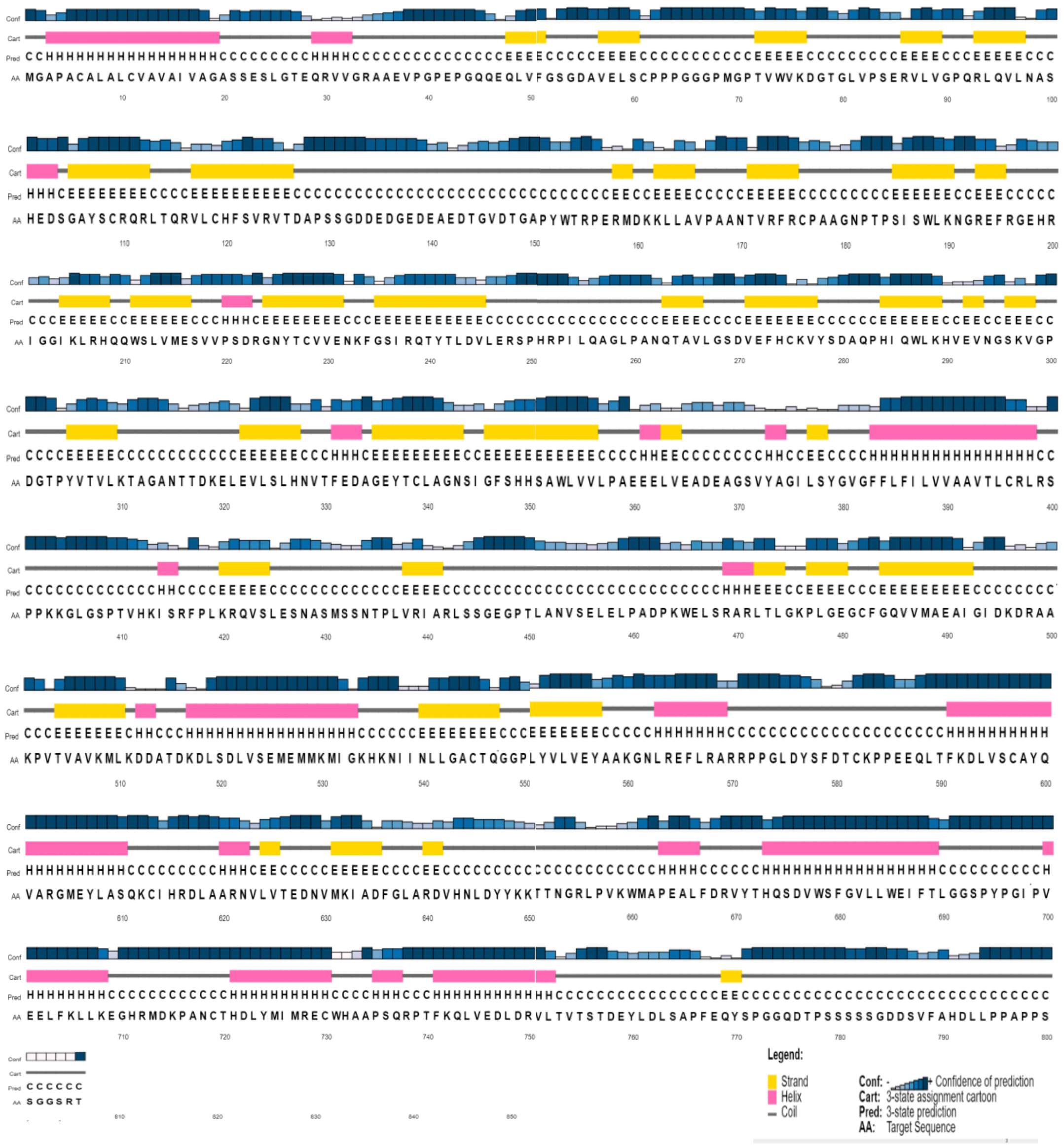

Figure S2: Secondary structure of FGFR3

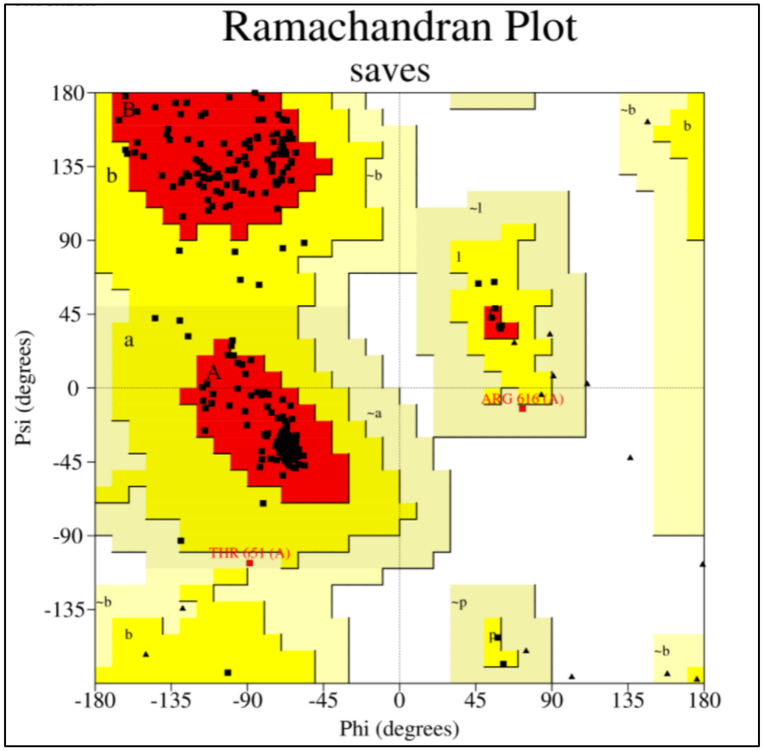

Plot statistics

Residues in most favoured regions [A,B,L]	234	91.8%
Residues in additional allowed regions [a,b,l,p]	19	7.5%
Residues in generously allowed regions [~a,~b,~l,~p]	2	0.8%
Residues in disallowed regions	0	0.0%

Figure S3: Ramachandran plot of FGFR3

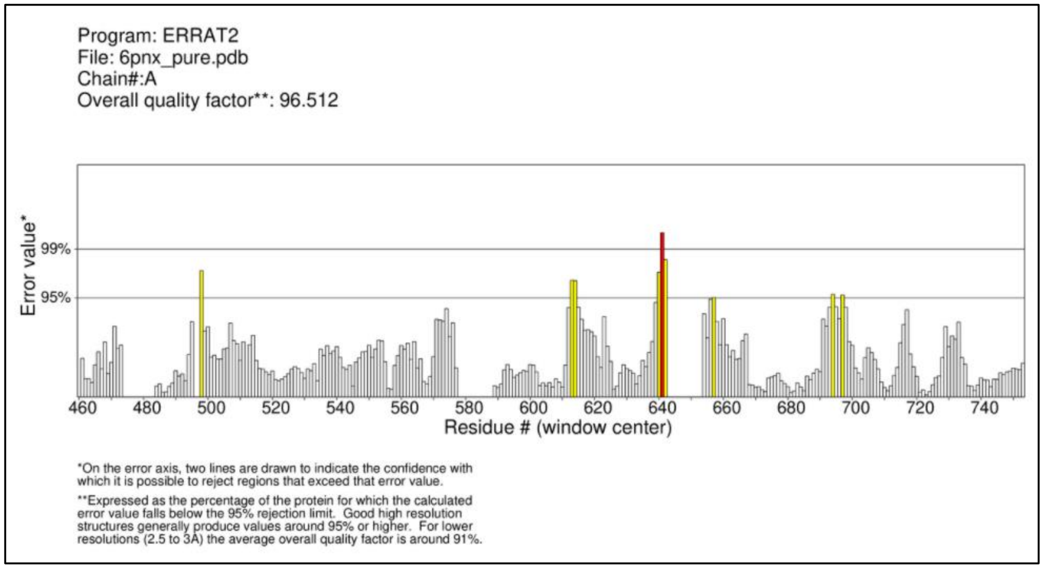

Figure S4: ERRAT plot of FGFR3 (overall quality factor: 96.512%)

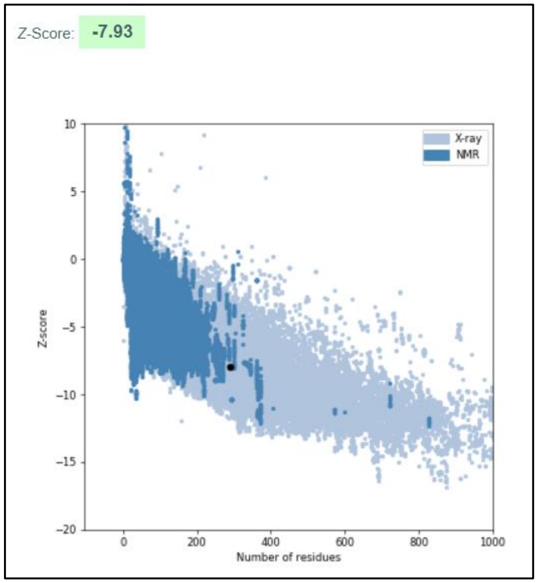

Figure S5: Quality factor assessment of FGFR3 using **ProsAweb server**

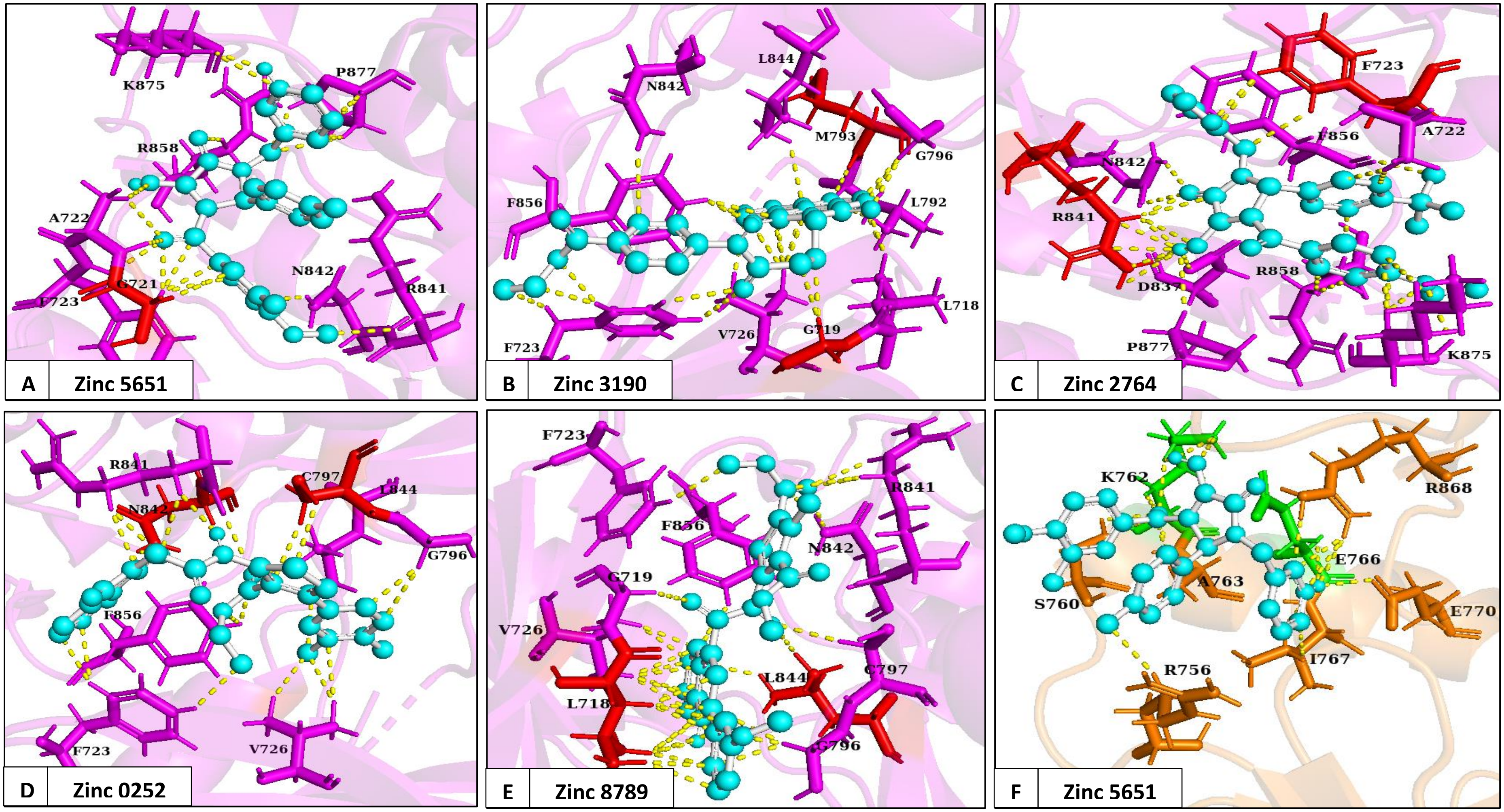

K875
P877
R858
A722
G721
N842
R841
A
Zinc 5651
N842
L844
M793
G796
L792
F856
L718
F723
V726
G719
B
Zinc 3190
F723
F856
A722
N842
R841
R858
D837
P877
K875
C
Zinc 2764
R841
C797
L844
N842
G796
F856
F723
V726
D
Zinc 0252
F723
R841
F856
N842
G719
V726
L844
C797
L718
G796
E
Zinc 8789
K762
R868
E766
A763
S760
E770
I767
R756
F
Zinc 5651

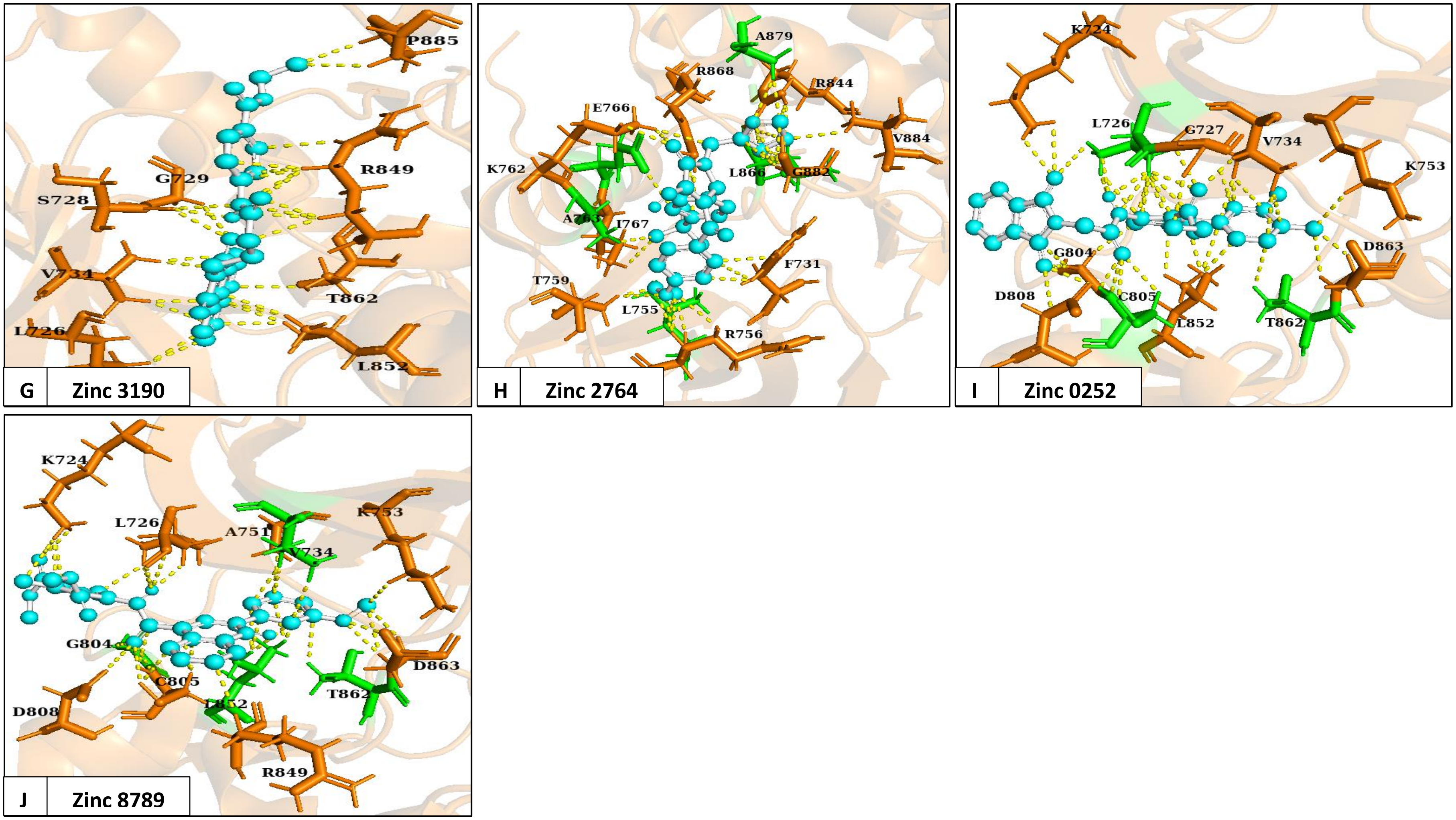

Figure S6: 3D docking structure of finalized ligands with EGFR (A-E) & ERBB2 (F-J). Yellow color indicates interaction (Hydrogen & hydrophobic bonds), Cyan represents ligands. The red & green color used in some pictures for distinguishing the interaction bonds.

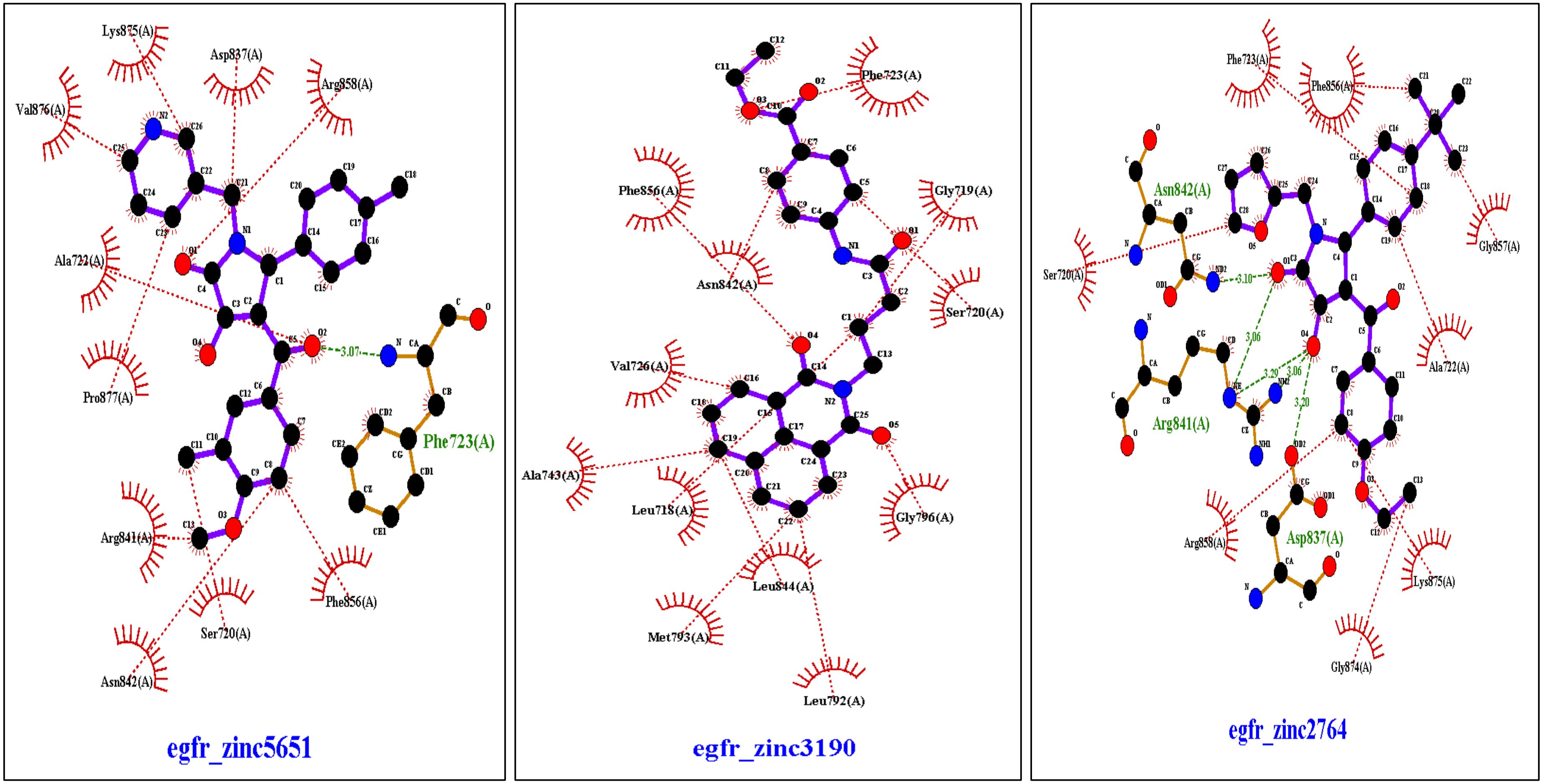
egfr_zinc5651
Lys875(A)
Asp837(A)
Arg858(A)
Val876(A)
Ala722(A)
Pro877(A)
Phe723(A)
3.07
Arg841(A)
Ser720(A)
Phe856(A)
Asn842(A)
egfr_zinc3190
Phe723(A)
Phe856(A)
Gly719(A)
Asn842(A)
Ser720(A)
Val726(A)
Ala743(A)
Leu718(A)
Gly796(A)
Leu844(A)
Met793(A)
Leu792(A)
egfr_zinc2764
Phe723(A)
Phe856(A)
Asn842(A)
Ser720(A)
Gly857(A)
3.10
3.06
3.20
Arg841(A)
Ala722(A)
Arg858(A)
Asp837(A)
Lys875(A)
Gly874(A)

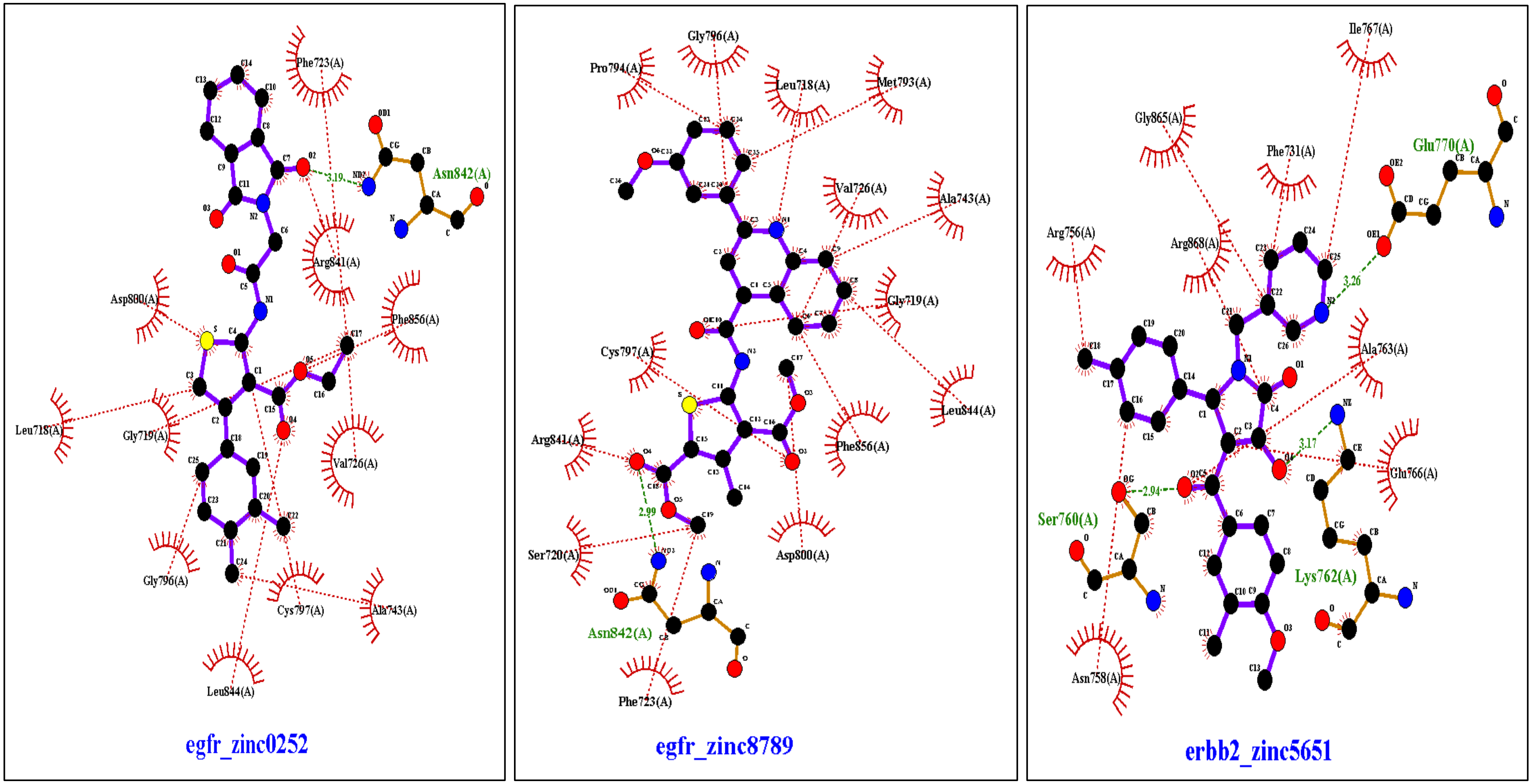

Phe723(A)
Asn842(A)
3.19
Arg841(A)
Asp800(A)
Phe856(A)
Leu718(A)
Gly719(A)
Val726(A)
Gly796(A)
Cys797(A)
Ala743(A)
Leu844(A)
egfr_zinc0252
Gly796(A)
Pro794(A)
Leu718(A)
Met793(A)
Val726(A)
Ala743(A)
Gly719(A)
Cys797(A)
Leu844(A)
Arg841(A)
Phe856(A)
2.99
Ser720(A)
Asp800(A)
Asn842(A)
Phe723(A)
egfr_zinc8789
Ile767(A)
Gly865(A)
Phe731(A)
Glu770(A)
Arg756(A)
Arg868(A)
3.26
Ala763(A)
3.17
Glu766(A)
2.94
Ser760(A)
Lys762(A)
Asn758(A)
erbb2_zinc5651

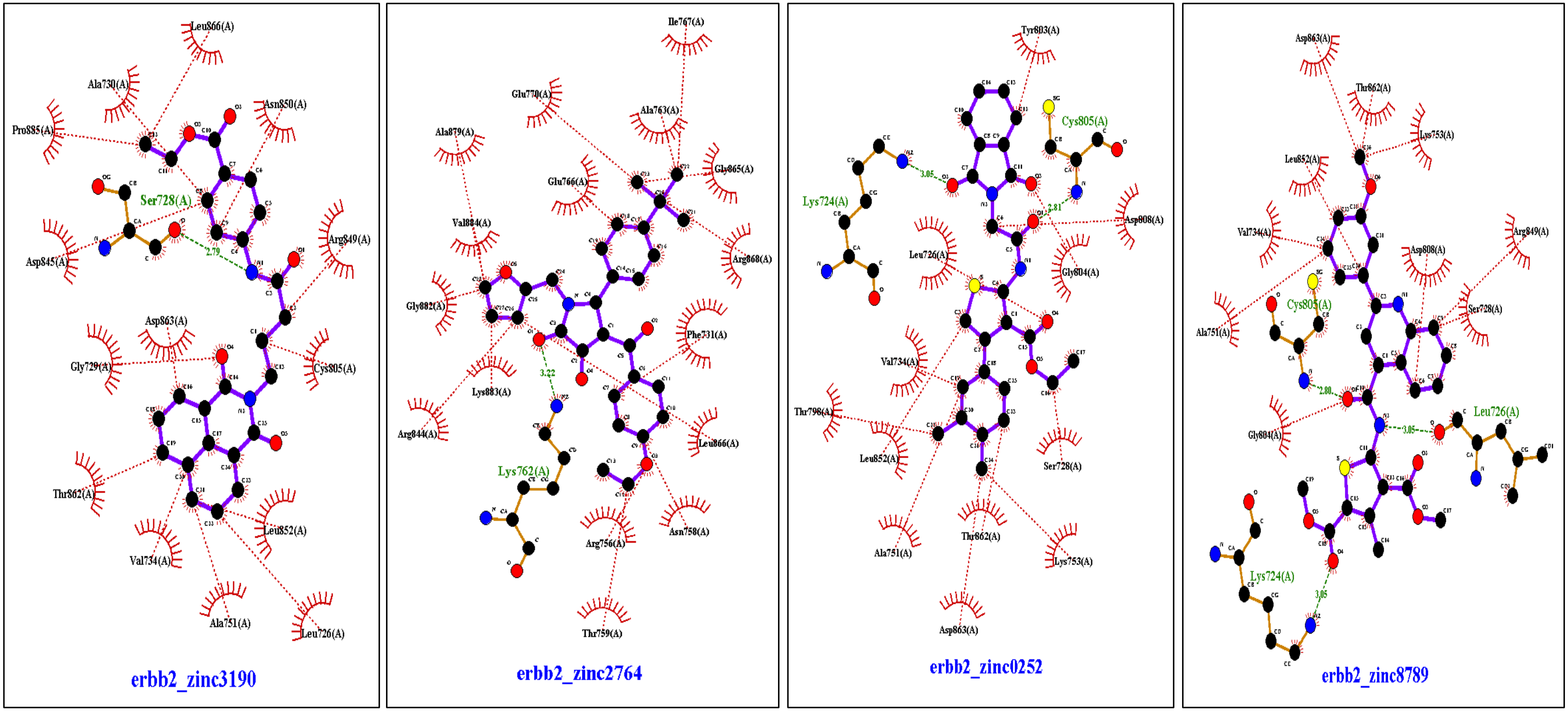

Figure S7: 2D docking structure of finalized ligands with EGFR & ERBB2. The green color indicates H-bonds and red color signifies hydrophobic contacts.

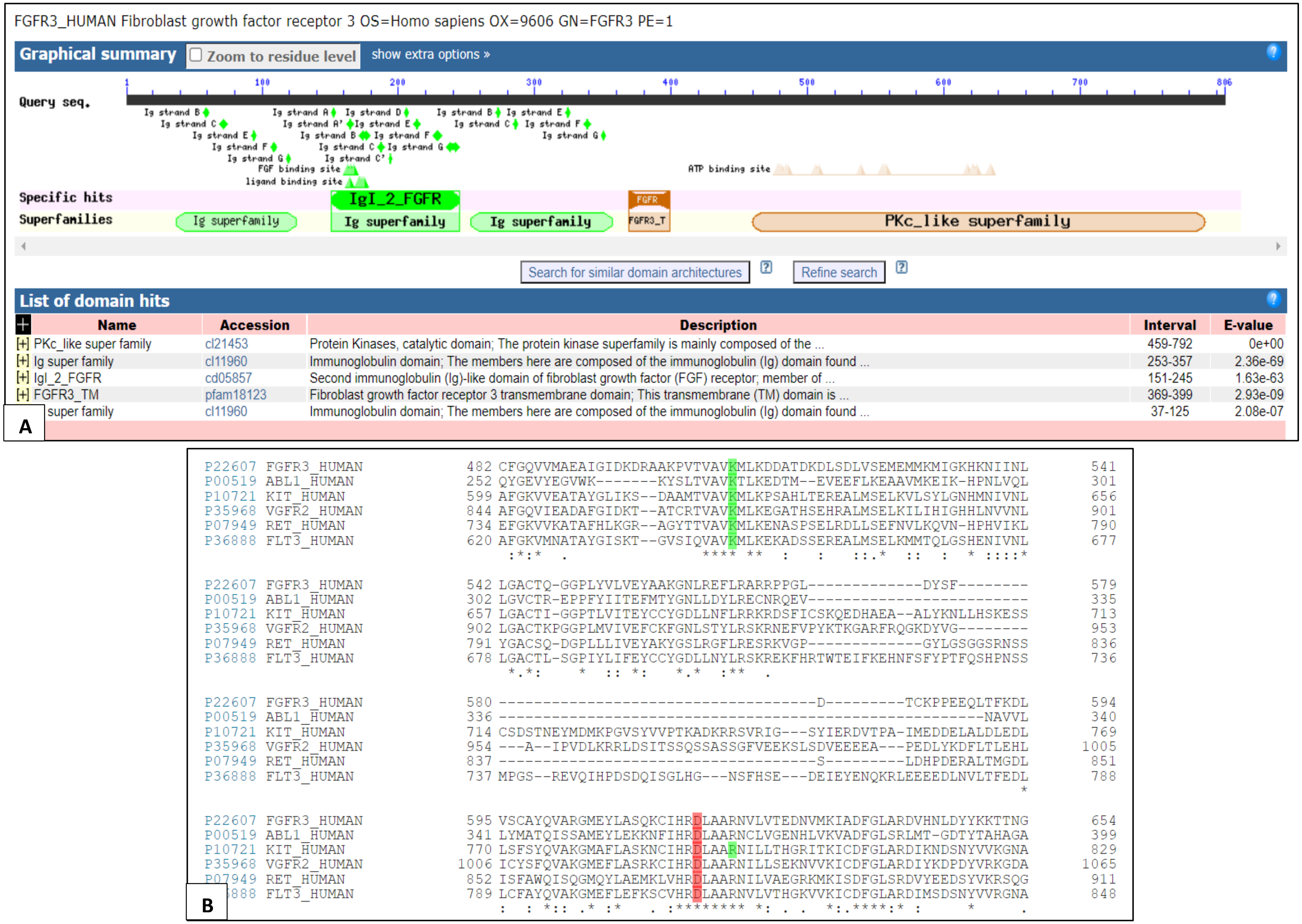

FGFR3_HUMAN Fibroblast growth factor receptor 3 OS=Homo sapiens OX=9606 GN=FGFR3 PE=1
Graphical summary
Zoom to residue level
show extra options »
Query seq.
ATP binding site
FGF binding site
ligand binding site
Specific hits
IgI_2_FGFR
FGFR
Superfamilies
Ig superfamily
Ig superfamily
Ig superfamily
FGFR3_T
PKc_like superfamily
Search for similar domain architectures
Refine search
List of domain hits
Name | Accession | Description | Interval | E-value
[+] PKc_like super family | cl21453 | Protein Kinases, catalytic domain; The protein kinase superfamily is mainly composed of the ... | 459-792 | 0e+00
[+] Ig super family | cl11960 | Immunoglobulin domain; The members here are composed of the immunoglobulin (Ig) domain found ... | 253-357 | 2.36e-69
[+] IgI_2_FGFR | cd05857 | Second immunoglobulin (Ig)-like domain of fibroblast growth factor (FGF) receptor; member of ... | 151-245 | 1.63e-63
[+] FGFR3_TM | pfam18123 | Fibroblast growth factor receptor 3 transmembrane domain; This transmembrane (TM) domain is ... | 369-399 | 2.93e-09
super family | cl11960 | Immunoglobulin domain; The members here are composed of the immunoglobulin (Ig) domain found ... | 37-125 | 2.08e-07
A
P22607 FGFR3_HUMAN 482 CFGQVVMAEAIGIDKDRAAKPVTVAVKMLKDDATDKDLSDLVSEMEMMKMIGKHKNIINL 541
P00519 ABL1_HUMAN 252 QYGEVYEGVWK-------KYSLTVAVKTLKEDTM--EVEEFLKEAAVMKEIK-HPNLVQL 301
P10721 KIT_HUMAN 599 AFGKVVEATAYGLIKS--DAAMTVAVKMLKPSAHLTEREALMSELKVLSYLGNHMNIVNL 656
P35968 VGFR2_HUMAN 844 AFGQVIEADAFGIDKT--ATCRTVAVKMLKEGATHSEHRALMSELKILIHIGHHLNVVNL 901
P07949 RET_HUMAN 734 EFGKVVKATAFHLKGR--AGYTTVAVKMLKENASPSELRDLLSEFNVLKQVN-HPHVIKL 790
P36888 FLT3_HUMAN 620 AFGKVMNATAYGISKT--GVSIQVAVKMLKEKADSSEREALMSELKMMTQLGSHENIVNL 677
P22607 FGFR3_HUMAN 542 LGACTQ-GGPLYVLVEYAAKGNLREFLRARRPPGL------------DYSF-------- 579
P00519 ABL1_HUMAN 302 LGVCTR-EPPFYIITEFMTYGNLLDYLRECNRQEV------------------------ 335
P10721 KIT_HUMAN 657 LGACTI-GGPTLVITEYCCYGDLLNFLRRKRDSFICSKQEDHAEA--ALYKNLLHSKESS 713
P35968 VGFR2_HUMAN 902 LGACTKPGGPLMVIVEFCKFGNLSTYLRSKRNEFVPYKTKGARFRQGKDYVG-------- 953
P07949 RET_HUMAN 791 YGACSQ-DGPLLLIVEYAKYGSLRGFLRESRKVGP------------GYLGSGGSRNSS 836
P36888 FLT3_HUMAN 678 LGACTL-SGPIYLIFEYCCYGDLLNYLRSKREKFHRTWTEIFKEHNFSFYPTFQSHPNSS 736
P22607 FGFR3_HUMAN 580 ------------------------------------D---------TCKPPEEQLTFKDL 594
P00519 ABL1_HUMAN 336 ---NAVVL 340
P10721 KIT_HUMAN 714 CSDSTNEYMDMKPGVSYVVPTKADKRRSVRIG---SYIERDVTPA-IMEDDELALDLEDL 769
P35968 VGFR2_HUMAN 954 ---A--IPVDLKRRLDSITSSQSSASSGFVEEKSLSDVEEEEA---PEDLYKDFLTLEHL 1005
P07949 RET_HUMAN 837 ------------------------------------S---------LDHPDERALTMGDL 851
P36888 FLT3_HUMAN 737 MPGS--REVQIHPDSDQISGLHG---NSFHSE---DEIEYENQKRLEEEEDLNVLTFEDL 788
P22607 FGFR3_HUMAN 595 VSCAYQVARGMEYLASQKCIHRDLAARNVLVTEDNVMKIADFGLARDVHNLDYYKKTTNG 654
P00519 ABL1_HUMAN 341 LYMATQISSAMEYLEKKNFIHRDLAARNCLVGENHLVKVADFGLSRLMT-GDTYTAHAGA 399
P10721 KIT_HUMAN 770 LSFSYQVAKGMAFLASKNCIHRDLAARNILLTHGRITKICDFGLARDIKNDSNYVVKGNA 829
P35968 VGFR2_HUMAN 1006 ICYSFQVAKGMEFLASRKCIHRDLAARNILLSEKNVVKICDFGLARDIYKDPDYVRKGDA 1065
P07949 RET_HUMAN 852 ISFAWQISQGMQYLAEMKLVHRDLAARNILVAEGRKMKISDFGLSRDVYEEDSYVKRSQG 911
P36888 FLT3_HUMAN 789 LCFAYQVAKGMEFLEFKSCVHRDLAARNVLVTHGKVVKICDFGLARDIMSDSNYVVRGNA 848
B

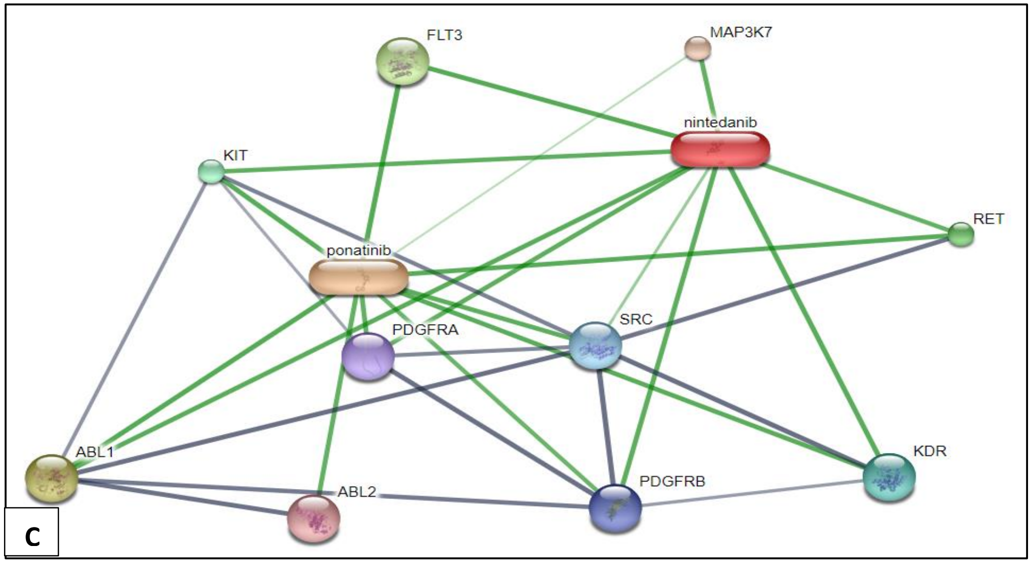

Figure S8. (A): Detection of conserved domains using NCBI (B): The alignment results of FGFR3, ABL1, KDR (VGFR), KIT, FLT3, RET (red color: active site, green color: binding site). (C): The interaction of ponatinib and nintedanib with ABL1, KDR, KIT, FLT3, RET

Table S3: molecular docking of finalized ligands with ABL1, KDR, KIT, FLT3, RET

Type of proteins	Molecule ID	Binding Affinity(kcal/mol)	Upper Bind RMSD	Lower Bind RMSD
**ABL1**	Ponatininb	-9.8	0.0	0.0
	Nintedanib	-8.5	0.0	0.0
	ZINC08433190	-9.6	0.0	0.0
	ZINC00668789	-9.2	0.0	0.0
	ZINC00710252	-9.0	0.0	0.0
	ZINC00702764	-8.5	0.0	0.0
	ZINC09045651	-7.6	0.0	0.0
**KDR**	Ponatininb	-9.1	0.0	0.0
	Nintedanib	-9.2	0.0	0.0
	ZINC08433190	-8.0	0.0	0.0
	ZINC00668789	-8.9	0.0	0.0
	ZINC00710252	-8.0	0.0	0.0
	ZINC00702764	-9.1	0.0	0.0
	ZINC09045651	-7.7	0.0	0.0
**KIT**	Ponatininb	-8.5	0.0	0.0
	Nintedanib	-8.1	0.0	0.0
	ZINC08433190	-8.0	0.0	0.0
	ZINC00668789	-7.6	0.0	0.0
	ZINC00710252	-7.6	0.0	0.0
	ZINC00702764	-6.9	0.0	0.0
	ZINC09045651	-7.2	0.0	0.0
**FLT3**	Ponatininb	-9.1	0.0	0.0
	Nintedanib	-8.6	0.0	0.0
	ZINC08433190	-8.7	0.0	0.0
	ZINC00668789	-8.5	0.0	0.0
	ZINC00710252	-8.8	0.0	0.0
	ZINC00702764	-7.3	0.0	0.0
	ZINC09045651	-7.7	0.0	0.0

**RET**	Ponatininb	-9.7	0.0	0.0
	Nintedanib	-8.5	0.0	0.0
	ZINC08433190	-9.3	0.0	0.0
	ZINC00668789	-9.5	0.0	0.0
	ZINC00710252	-9.2	0.0	0.0
	ZINC00702764	-7.2	0.0	0.0
	ZINC09045651	-7.8	0.0	0.0

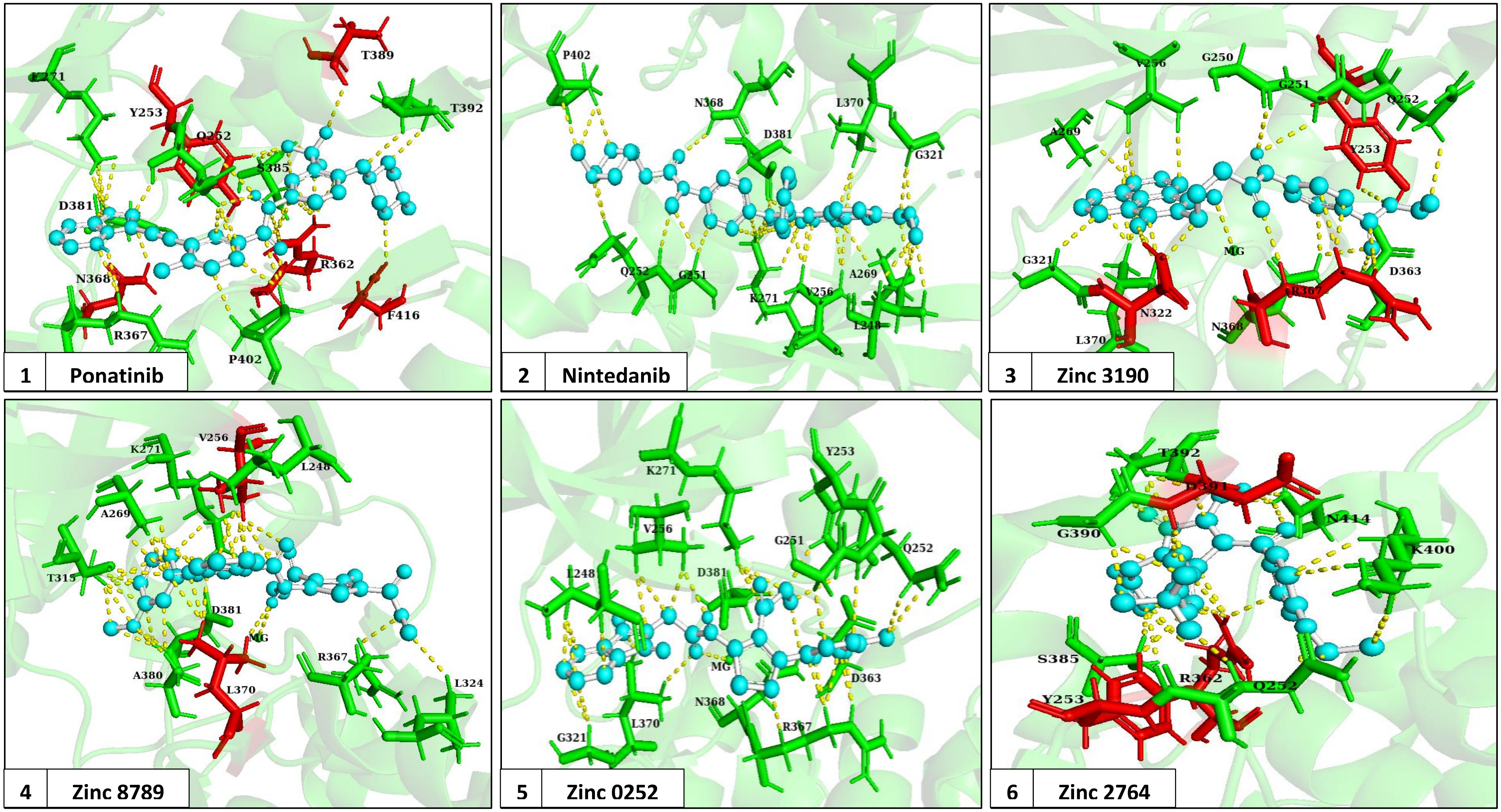
1
Ponatinib
T389
T392
Y253
Q252
S385
D381
R362
N368
F416
R367
P402
2
Nintedanib
P402
N368
L370
D381
G321
Q252
G251
K271
V256
A269
L248
3
Zinc 3190
V256
G250
G251
Q252
A269
Y253
G321
MG
D363
N322
R367
N368
L370
4
Zinc 8789
V256
K271
L248
A269
T315
D381
MG
A380
L370
R367
L324
5
Zinc 0252
K271
Y253
V256
G251
Q252
L248
D381
MG
D363
N368
R367
G321
L370
6
Zinc 2764
T392
D391
N414
G390
K400
S385
R362
Q252
Y253

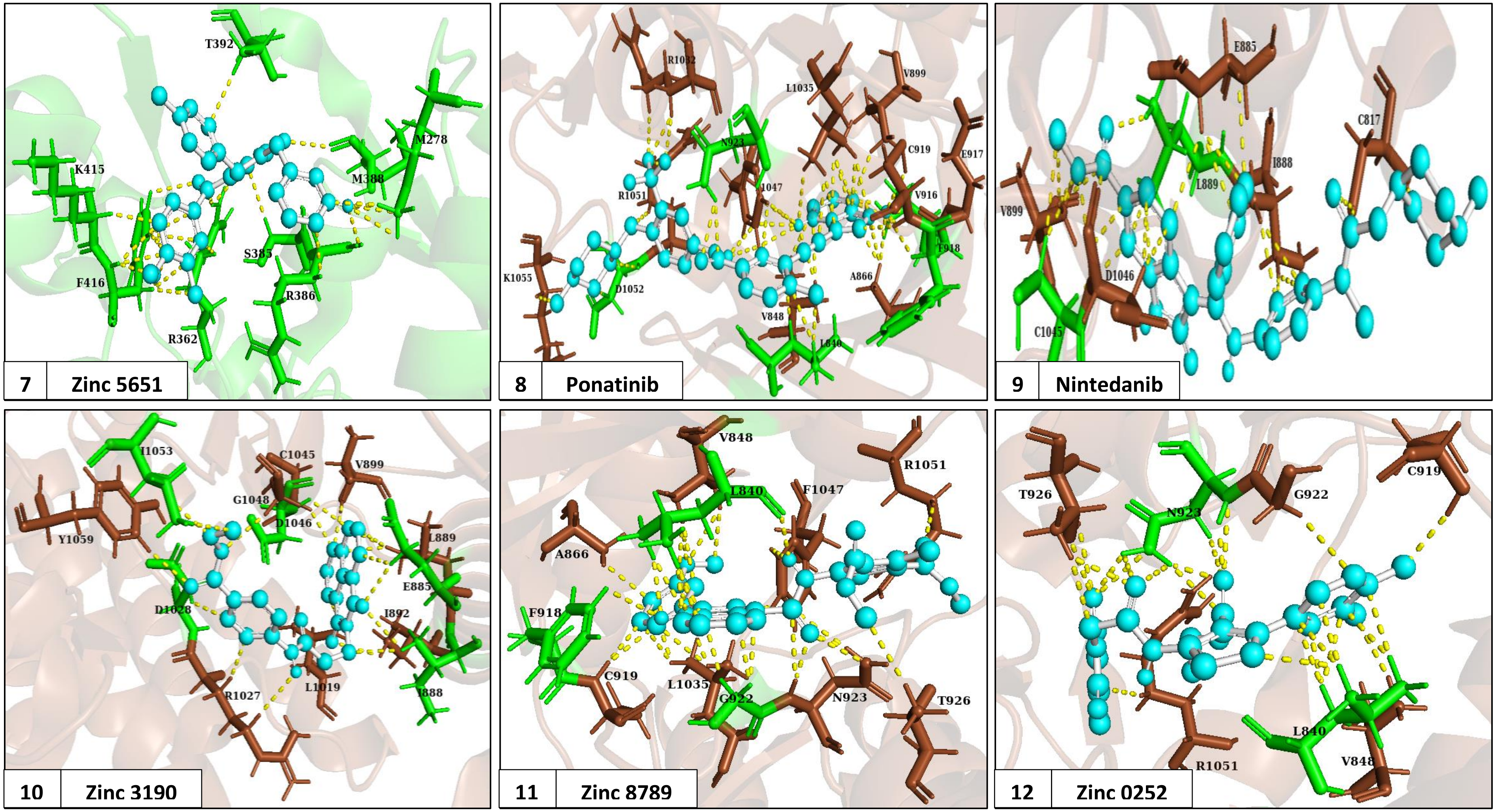

7
Zinc 5651
T392
M278
K415
M388
S385
F416
R386
R362
8
Ponatinib
R1032
L1035
V899
N923
C919
E917
R1051
V916
F918
K1055
D1052
A866
V848
L840
9
Nintedanib
E885
C817
I888
L889
V899
D1046
C1045
10
Zinc 3190
I1053
C1045
V899
G1048
D1046
Y1059
L889
E885
D1028
I892
R1027
L1019
I888
11
Zinc 8789
V848
R1051
L840
F1047
A866
F918
C919
L1035
G922
N923
T926
12
Zinc 0252
T926
G922
C919
N923
L840
V848
R1051

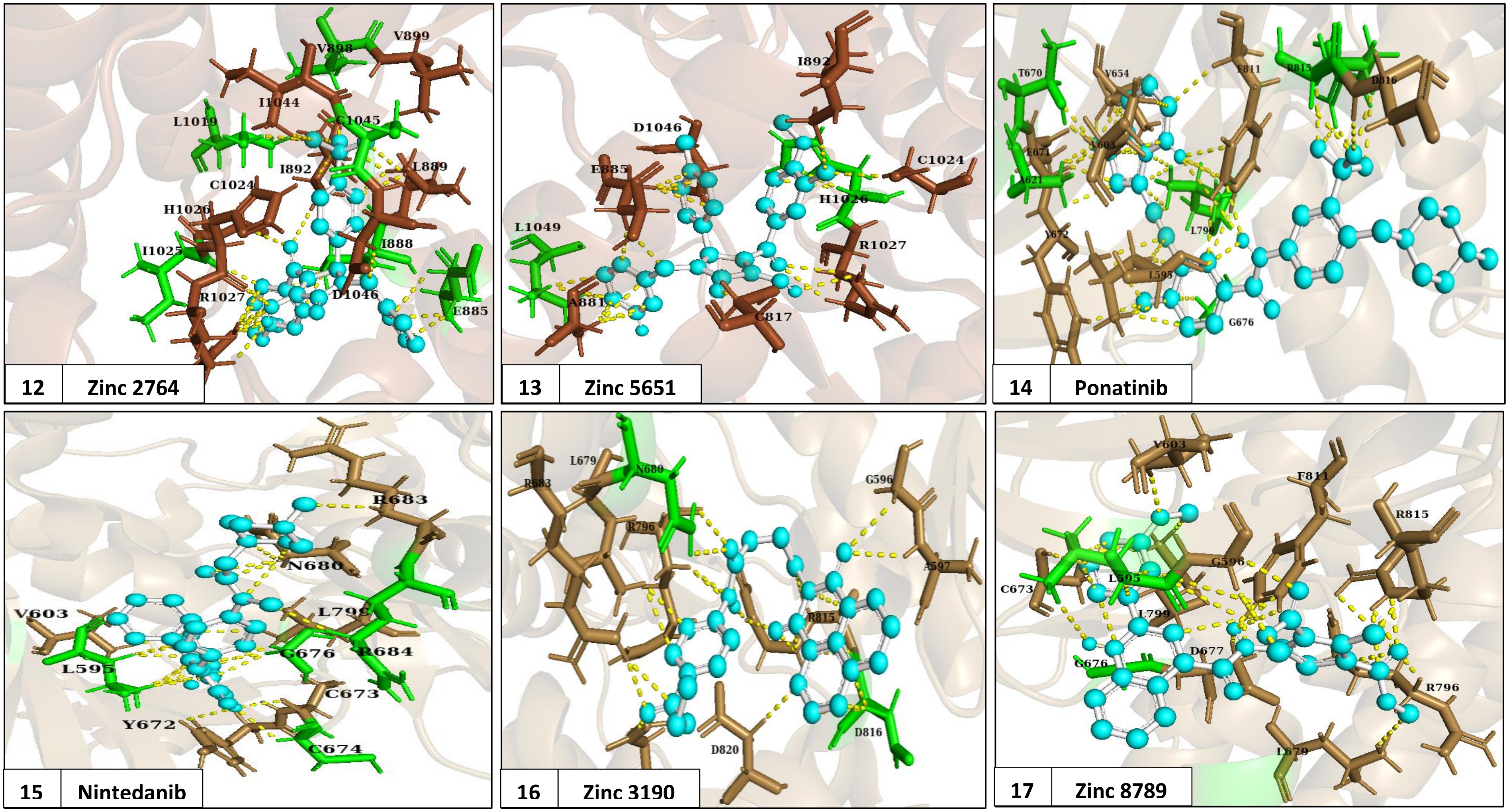
V898
V899
I1044
L1019
C1045
I892
L889
C1024
H1026
I888
I1025
R1027
D1046
E885
12 Zinc 2764
I892
D1046
E885
C1024
H1026
L1049
R1027
A881
C817
13 Zinc 5651
T670
V654
F811
R815
D816
E671
V603
A621
Y672
L799
L595
G676
14 Ponatinib
R683
N680
V603
L799
G676
R684
L595
C673
Y672
C674
15 Nintedanib
L679
N680
R683
G596
R796
A597
R815
D820
D816
16 Zinc 3190
V603
F811
R815
G596
C673
L595
L799
D677
G676
R796
L679
17 Zinc 8789

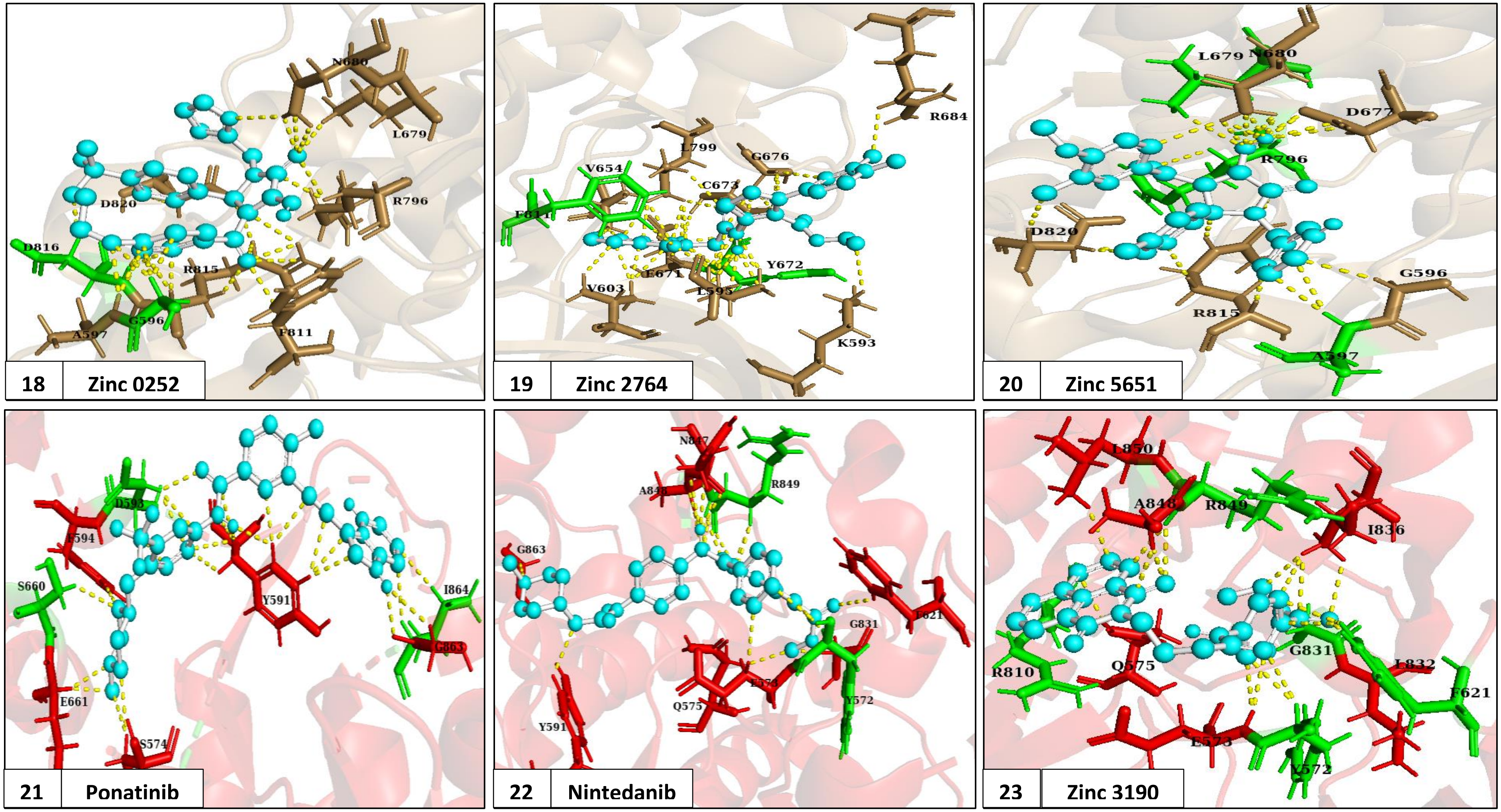

N680
L679
R796
D820
D816
R815
A597
G596
F811
18
Zinc 0252
R684
L799
G676
V654
C673
F811
Y672
V603
E671
L595
K593
19
Zinc 2764
L679
N680
D677
R796
D820
G596
R815
A597
20
Zinc 5651
D593
F594
S660
Y591
I864
G863
E661
S574
21
Ponatinib
N847
A848
R849
G863
G831
Q575
Y591
Y572
22
Nintedanib
I850
A848
R849
I836
G831
I832
R810
Q575
F621
E573
Y572
23
Zinc 3190

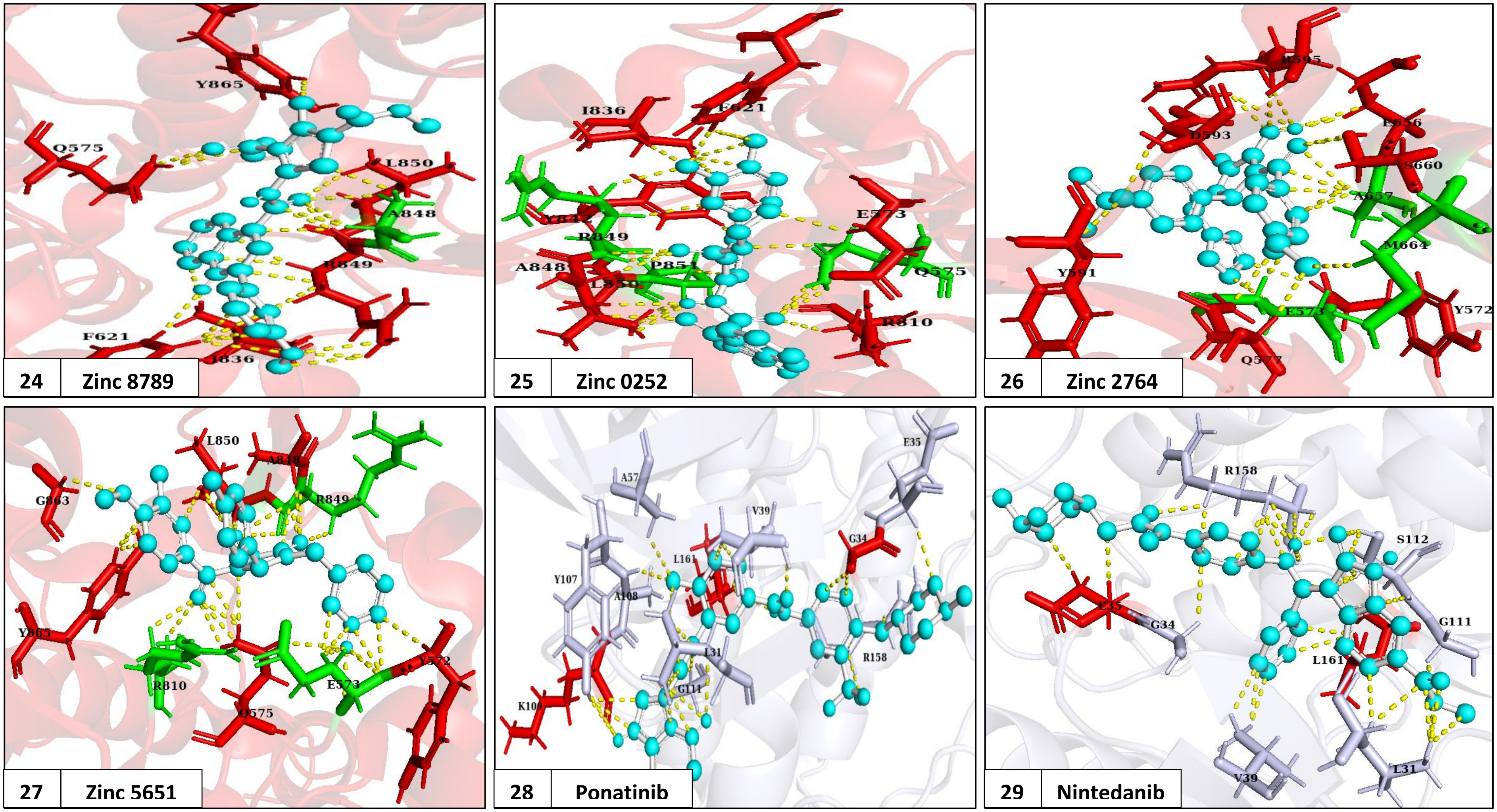
24
Zinc 8789
Y865
Q575
L850
A848
R849
F621
I836
25
Zinc 0252
I836
F621
Y842
R849
A848
P851
E573
Q575
R810
26
Zinc 2764
R595
D593
E656
S660
A657
M664
Y591
E573
Y572
Q577
27
Zinc 5651
L850
A848
G863
R849
Y865
R810
Q575
E573
Y572
28
Ponatinib
E35
A57
V39
G34
Y107
L161
A108
L31
R158
G111
K100
29
Nintedanib
R158
S112
E35
G34
G111
L161
V39
L31

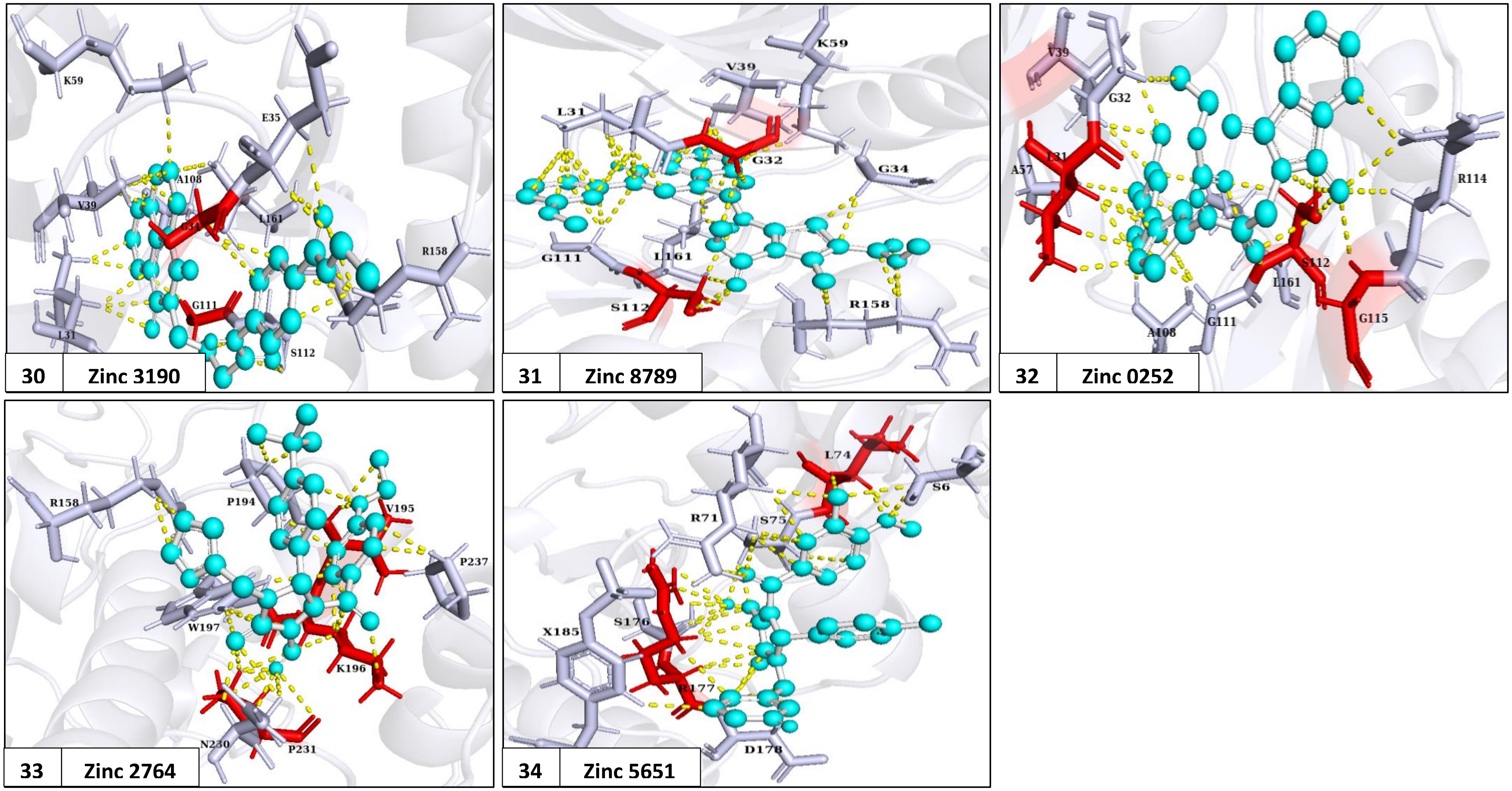

Figure S9: 3D structure of docking ABL1 (1-7), KDR (8-13), KIT (14-20), FLT3 (21-27), RET (28-34). Yellow color indicates interaction (Hydrogen & hydrophobic bonds), Cyan represents ligands. The red & green color used in some pictures for distinguishing the interaction bonds.

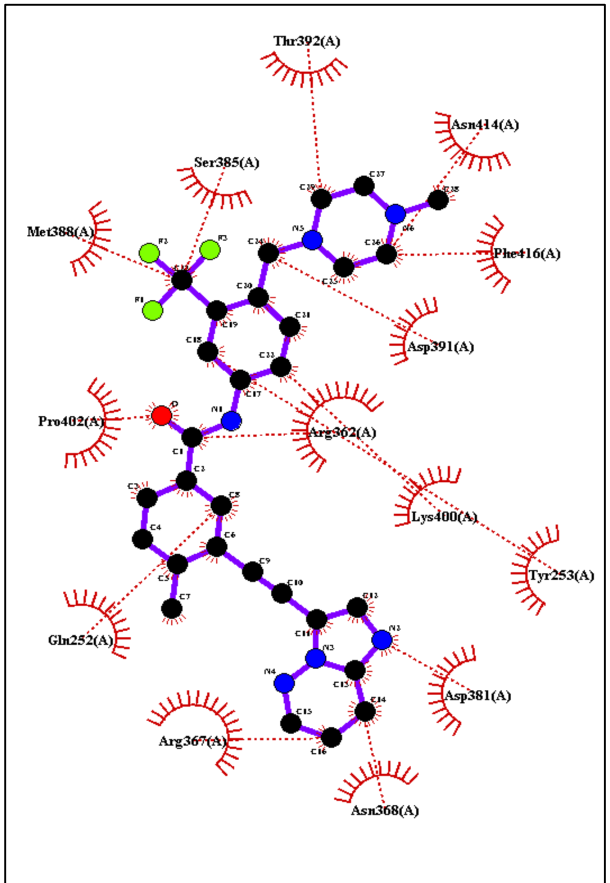

abl1_ponatinib

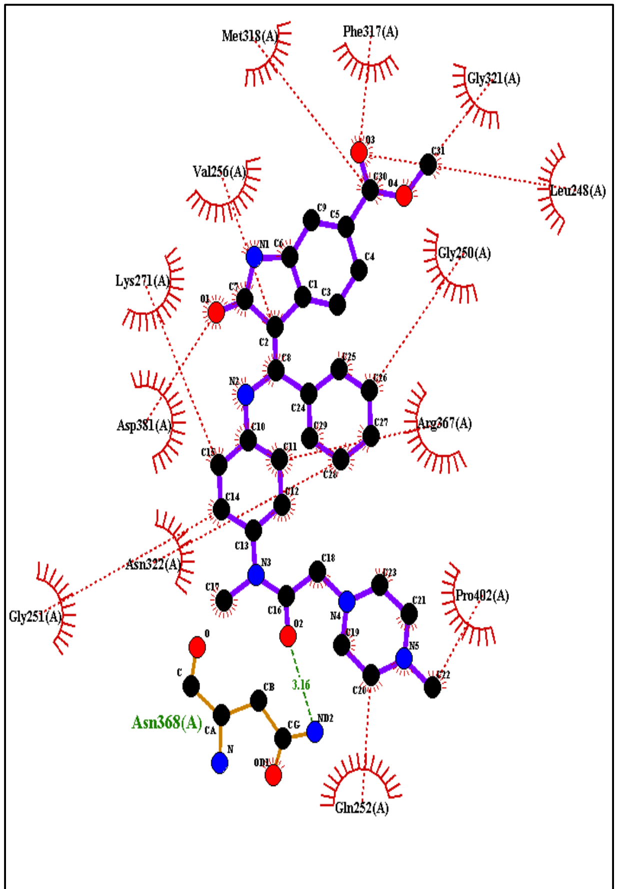

abl1_nintedanib

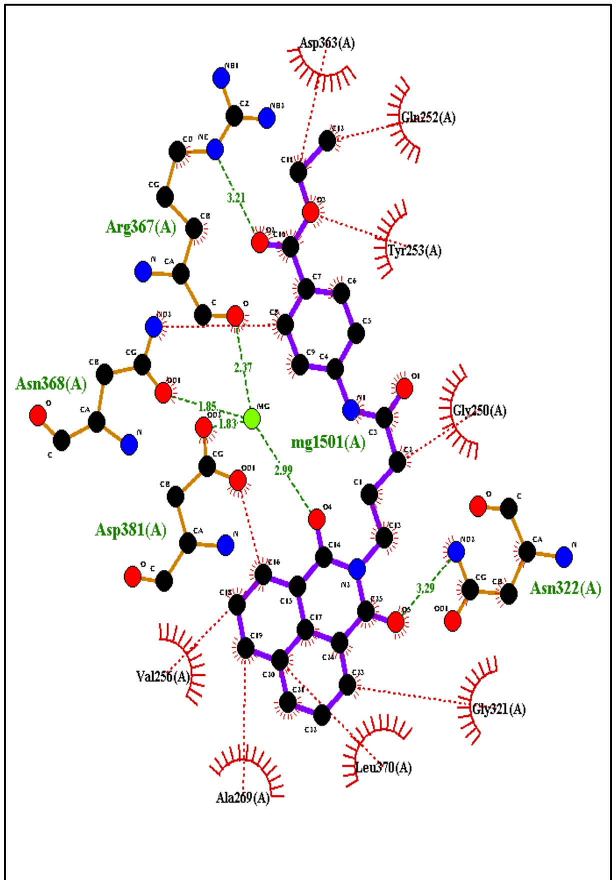

abl1_zinc3190

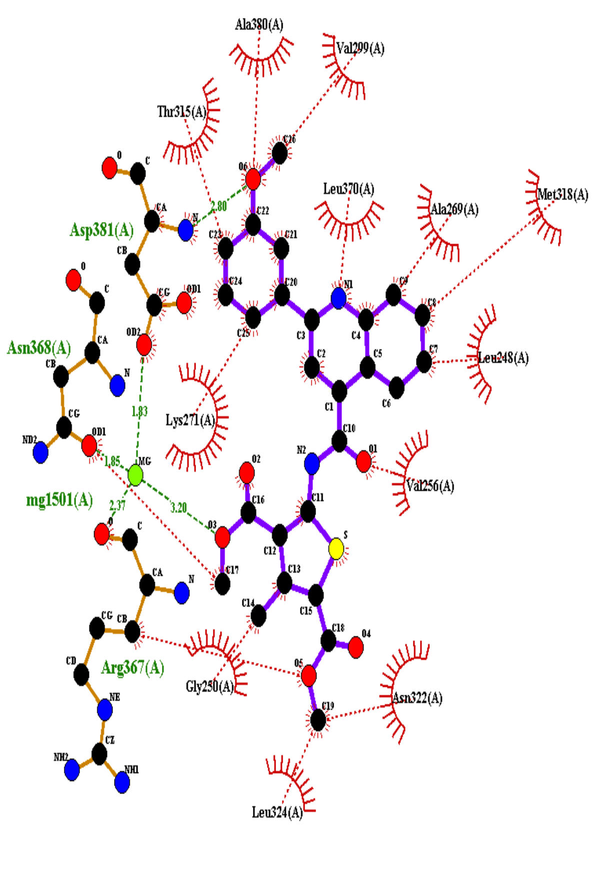

abl1_zinc8789

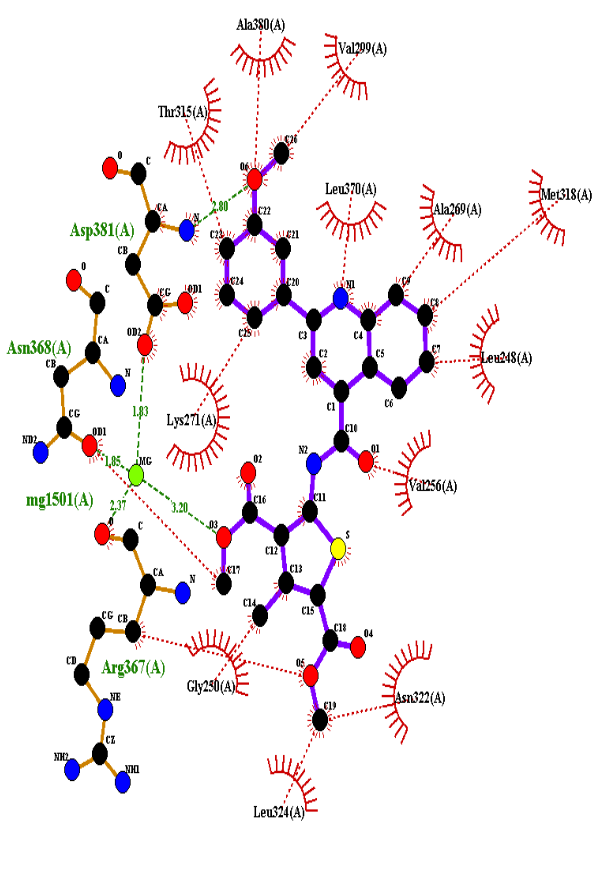

abl1_zinc8789

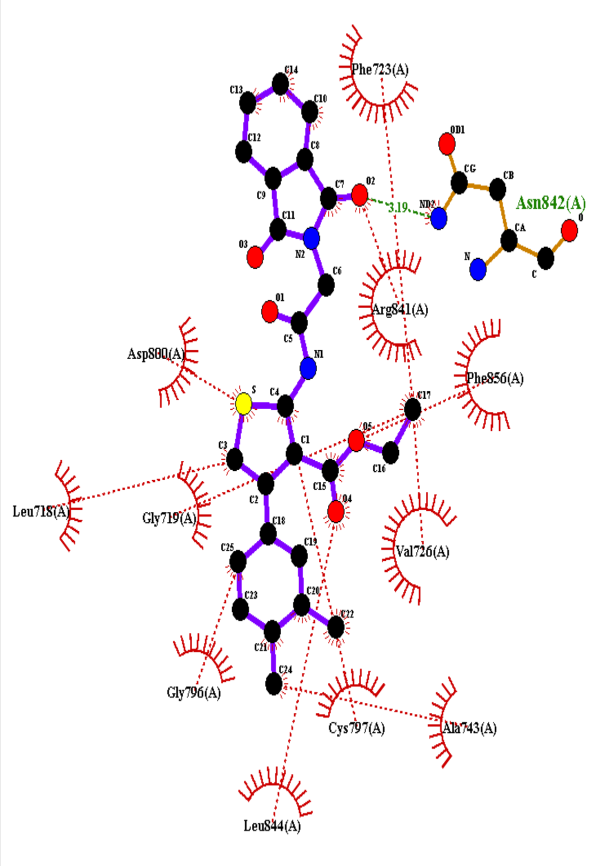

egfr_zinc0252

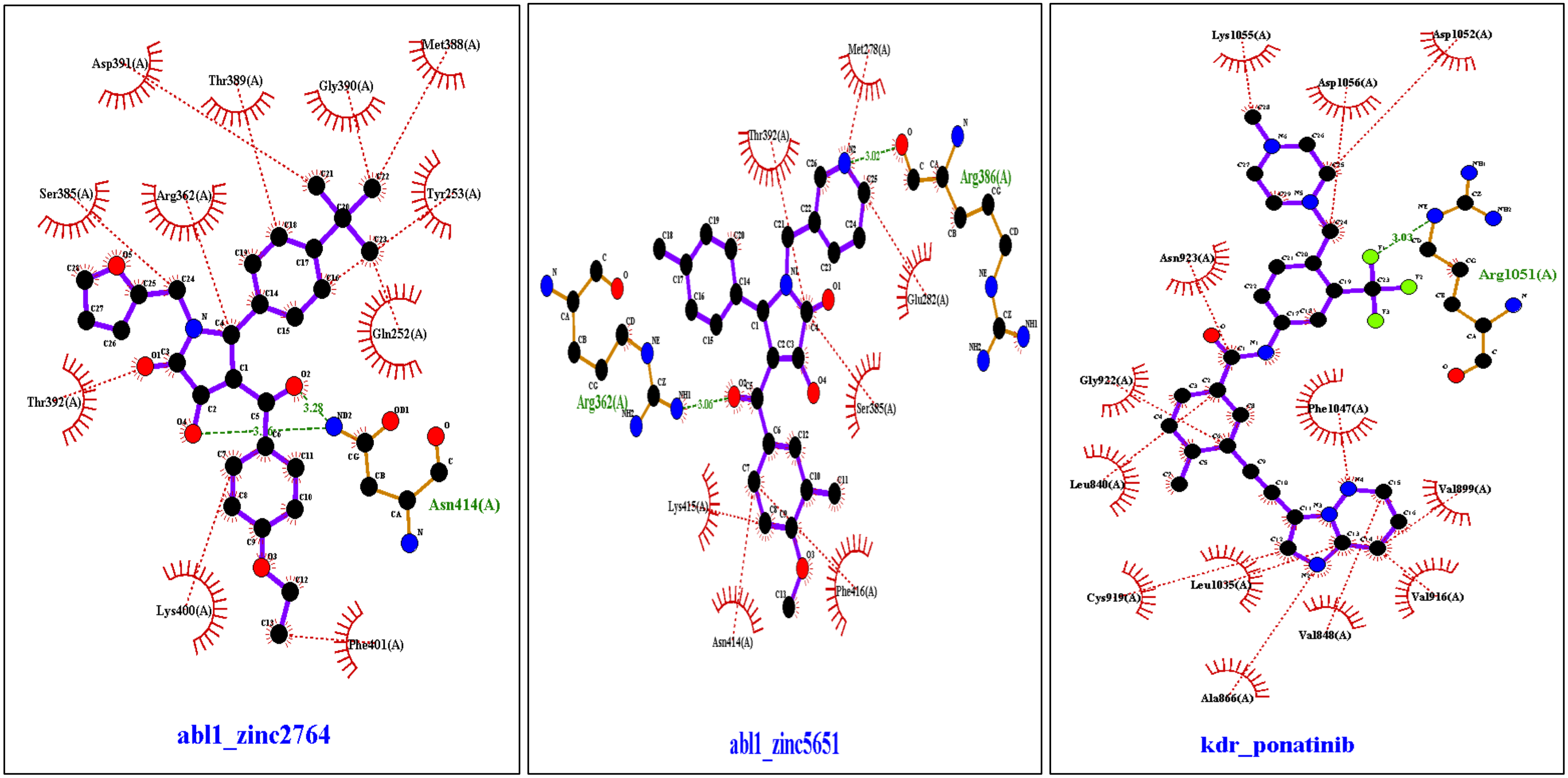
Asp391(A)
Thr389(A)
Gly390(A)
Met388(A)
Ser385(A)
Arg362(A)
Tyr253(A)
Gln252(A)
Thr392(A)
3.28
Asn414(A)
Lys400(A)
Phe401(A)
abl1_zinc2764
Met278(A)
Thr392(A)
3.02
Arg386(A)
Glu282(A)
Arg362(A)
3.06
Ser385(A)
Lys415(A)
Phe416(A)
Asn414(A)
abl1_zinc5651
Lys1055(A)
Asp1052(A)
Asp1056(A)
Asn923(A)
3.03
Arg1051(A)
Gly922(A)
Phe1047(A)
Leu840(A)
Val899(A)
Cys919(A)
Leu1035(A)
Val916(A)
Val848(A)
Ala866(A)
kdr_ponatinib

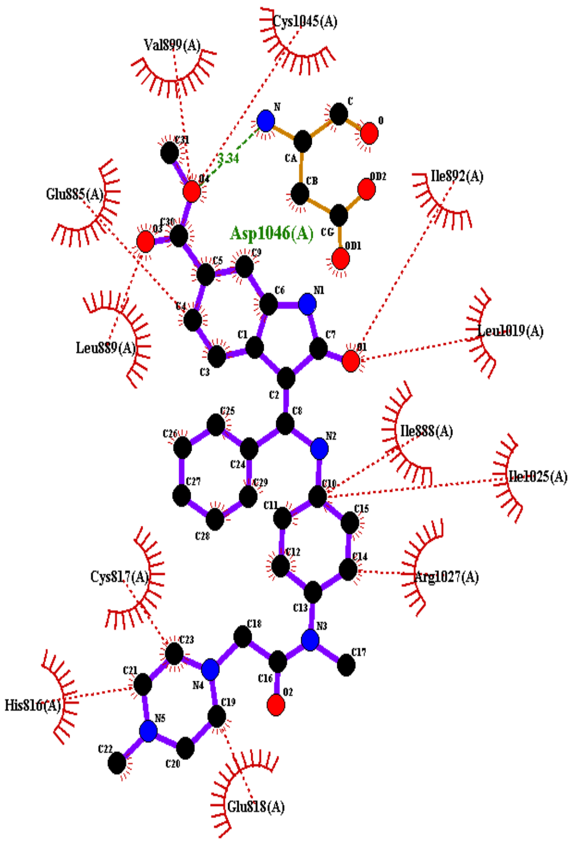

kdr_nintedanib

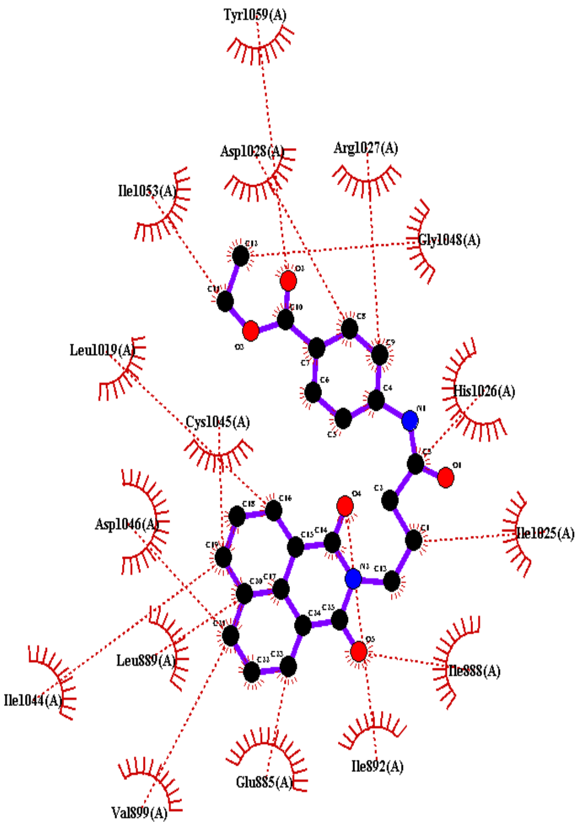

kdr_zinc3190

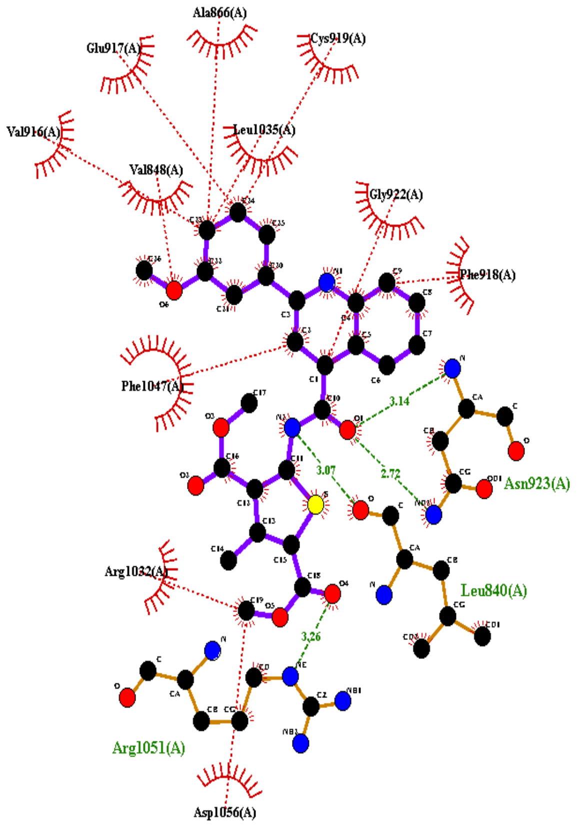

kdr_zinc8789

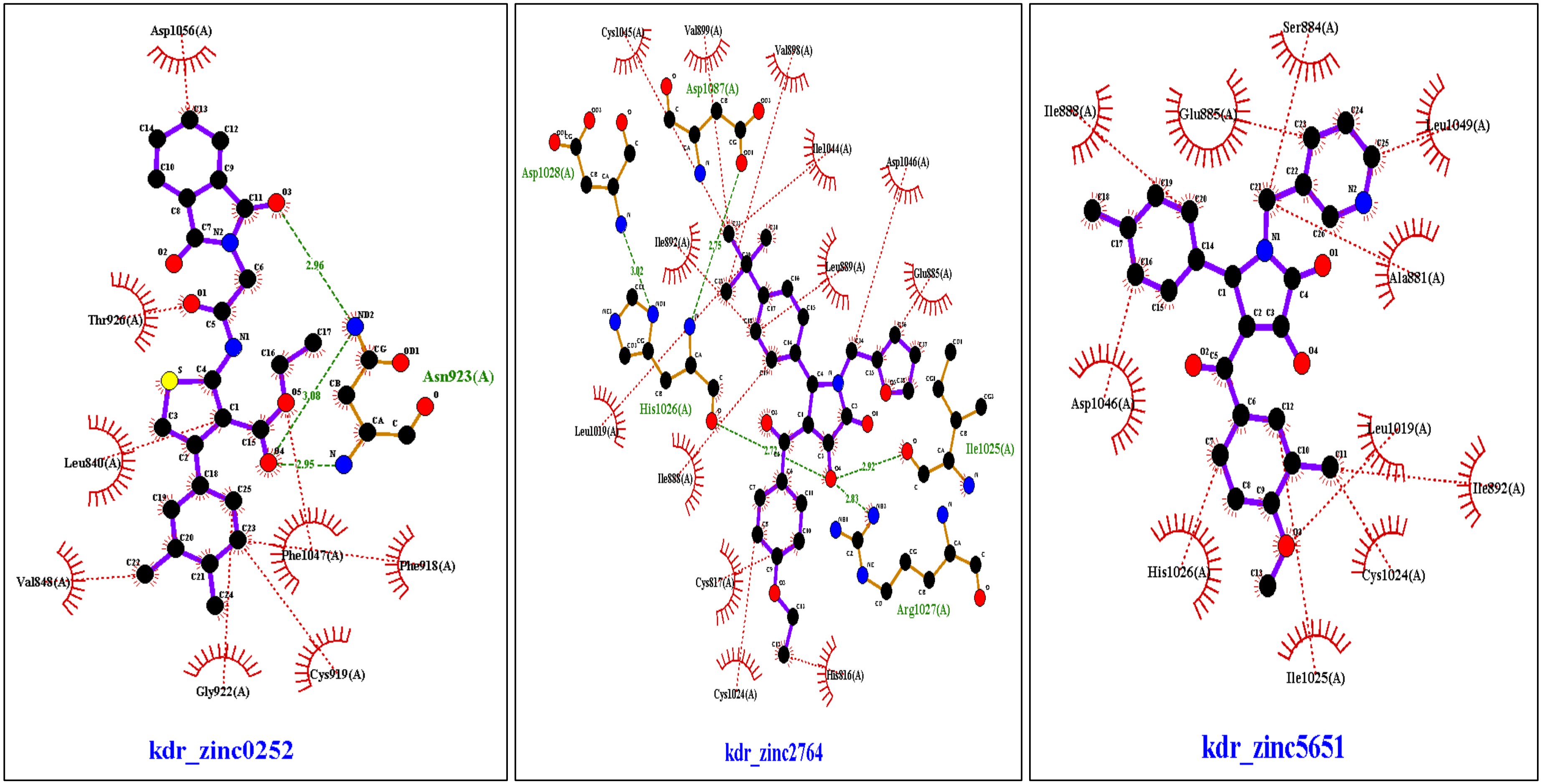
Asp1056(A)
Thr926(A)
Asn923(A)
Leu840(A)
Val848(A)
Phe1047(A)
Phe918(A)
Gly922(A)
Cys919(A)
kdr_zinc0252
Cys1045(A)
Val899(A)
Val898(A)
Asp1087(A)
Asp1028(A)
Ile1044(A)
Asp1046(A)
Ile892(A)
Leu889(A)
Glu885(A)
His1026(A)
Leu1019(A)
Ile888(A)
Ile1025(A)
Cys817(A)
Arg1027(A)
Cys1024(A)
His816(A)
kdr_zinc2764
Ser884(A)
Ile888(A)
Glu885(A)
Leu1049(A)
Ala881(A)
Asp1046(A)
Leu1019(A)
Ile892(A)
His1026(A)
Cys1024(A)
Ile1025(A)
kdr_zinc5651

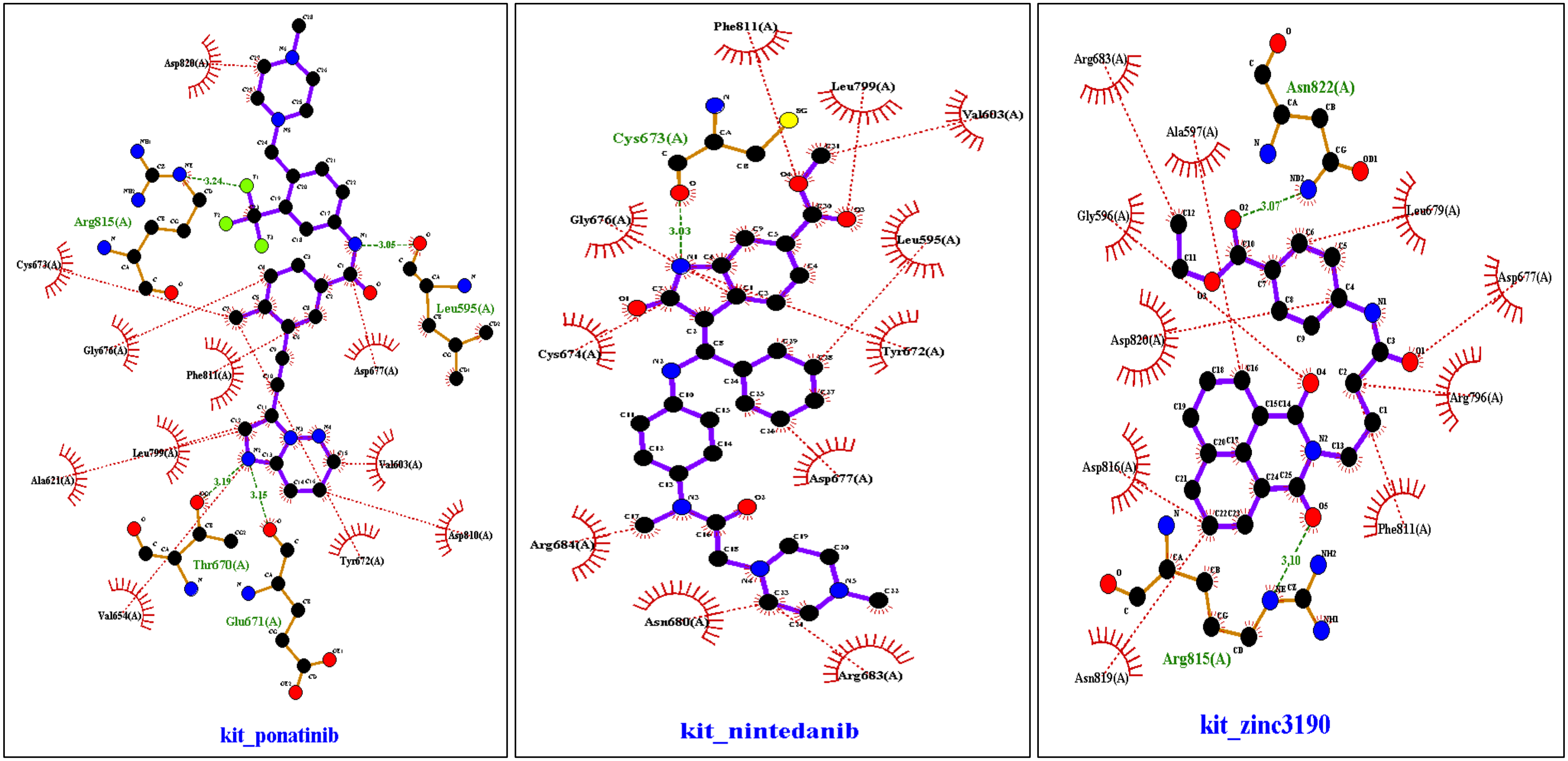
kit_ponatinib
Asp820(A)
Arg815(A)
Cys673(A)
Gly676(A)
Phe811(A)
Asp677(A)
Leu595(A)
Leu799(A)
Ala621(A)
Val603(A)
Thr670(A)
Val654(A)
Glu671(A)
Tyr672(A)
Asp810(A)
3.24
3.05
3.19
3.15
kit_nintedanib
Phe811(A)
Leu799(A)
Val603(A)
Cys673(A)
Gly676(A)
Leu595(A)
Cys674(A)
Tyr672(A)
Asp677(A)
Arg684(A)
Asn680(A)
Arg683(A)
3.03
kit_zinc3190
Arg683(A)
Asn822(A)
Ala597(A)
Gly596(A)
Leu679(A)
Asp677(A)
Asp820(A)
Arg796(A)
Asp816(A)
Phe811(A)
Arg815(A)
Asn819(A)
3.07
3.10

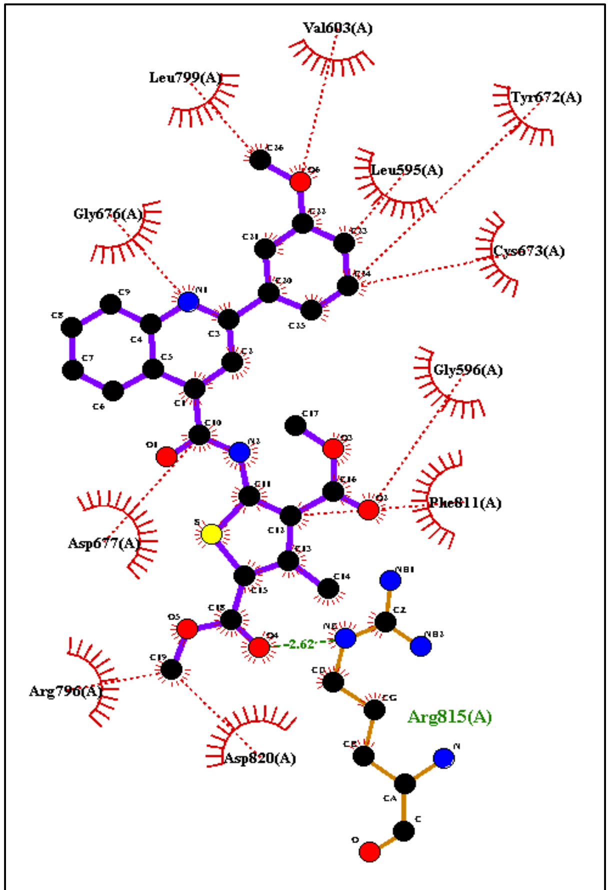

kit_zinc8789

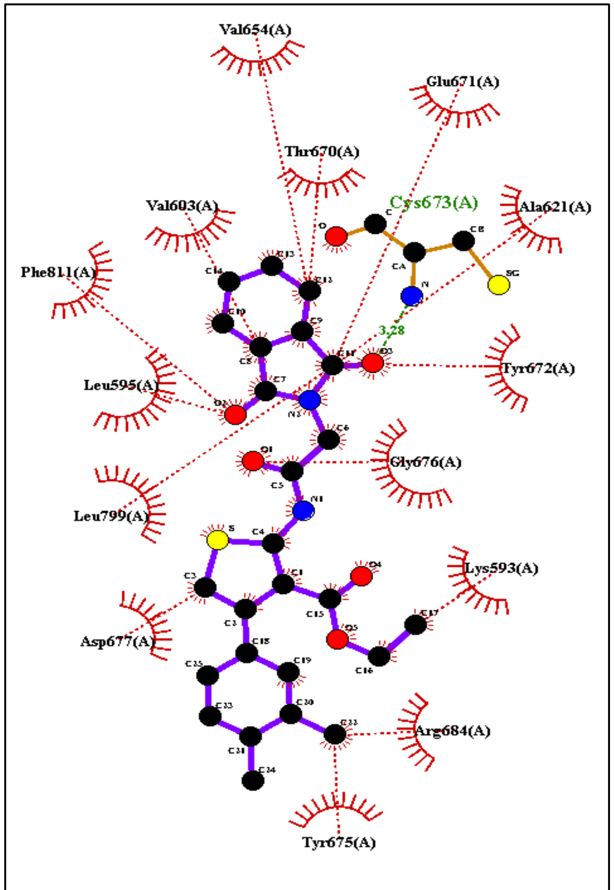

kit_zinc0252

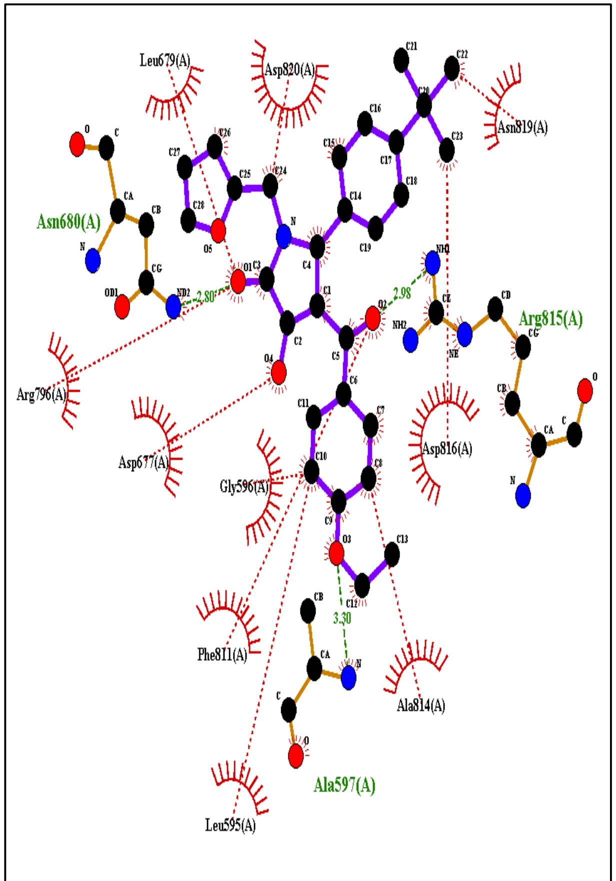

kit_zinc2764

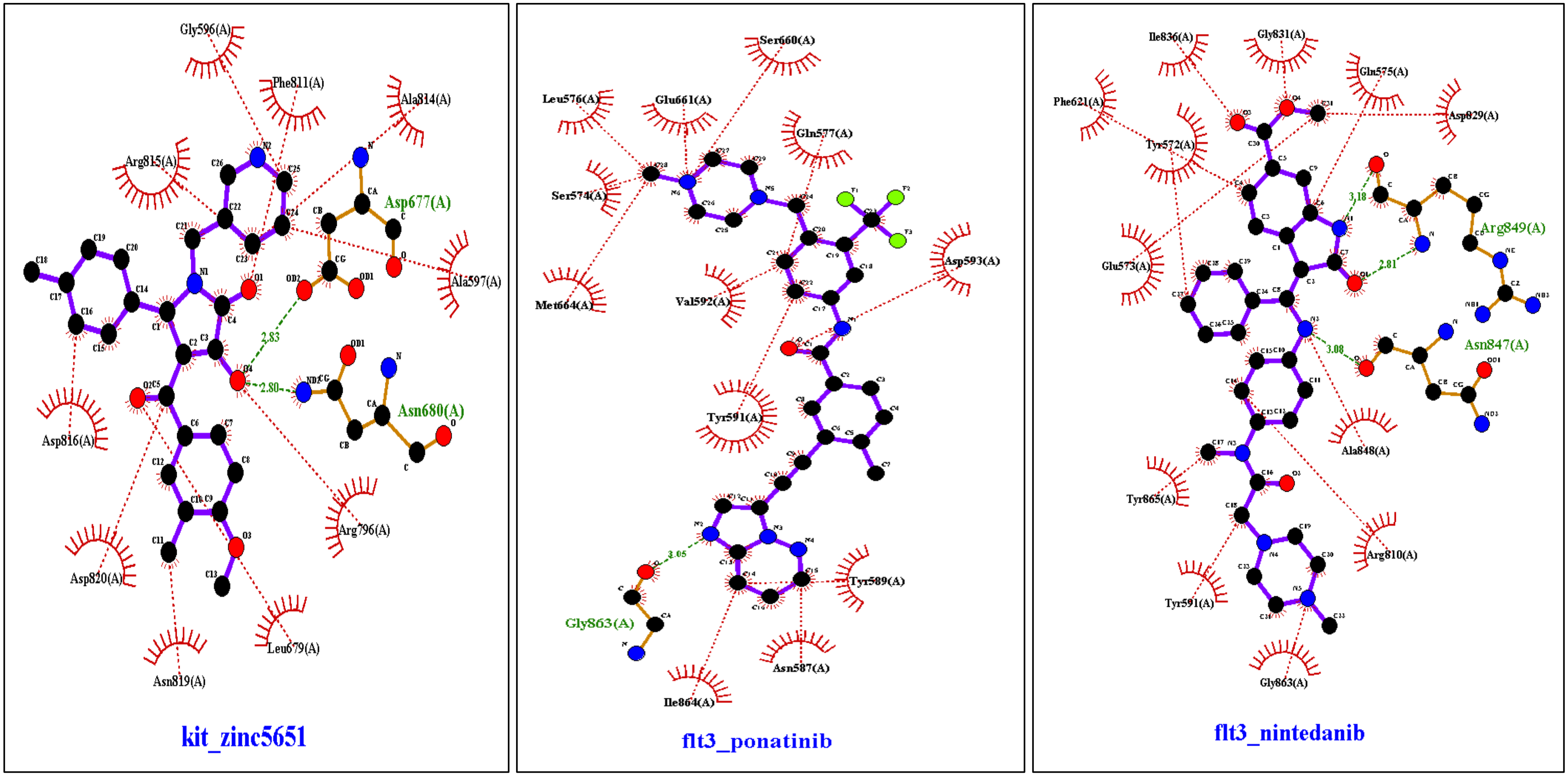
Gly596(A)
Phe811(A)
Ala814(A)
Arg815(A)
Asp677(A)
Ala597(A)
2.83
2.80
Asn680(A)
Asp816(A)
Arg796(A)
Asp820(A)
Leu679(A)
Asn819(A)
kit_zinc5651
Ser660(A)
Leu576(A)
Glu661(A)
Gln577(A)
Ser574(A)
Asp593(A)
Met664(A)
Val592(A)
Tyr591(A)
3.05
Tyr589(A)
Gly863(A)
Asn587(A)
Ile864(A)
flt3_ponatinib
Ile836(A)
Gly831(A)
Gln575(A)
Phe621(A)
Asp829(A)
Tyr572(A)
3.18
Arg849(A)
Glu573(A)
2.81
3.08
Asn847(A)
Ala848(A)
Tyr865(A)
Arg810(A)
Tyr591(A)
Gly863(A)
flt3_nintedanib

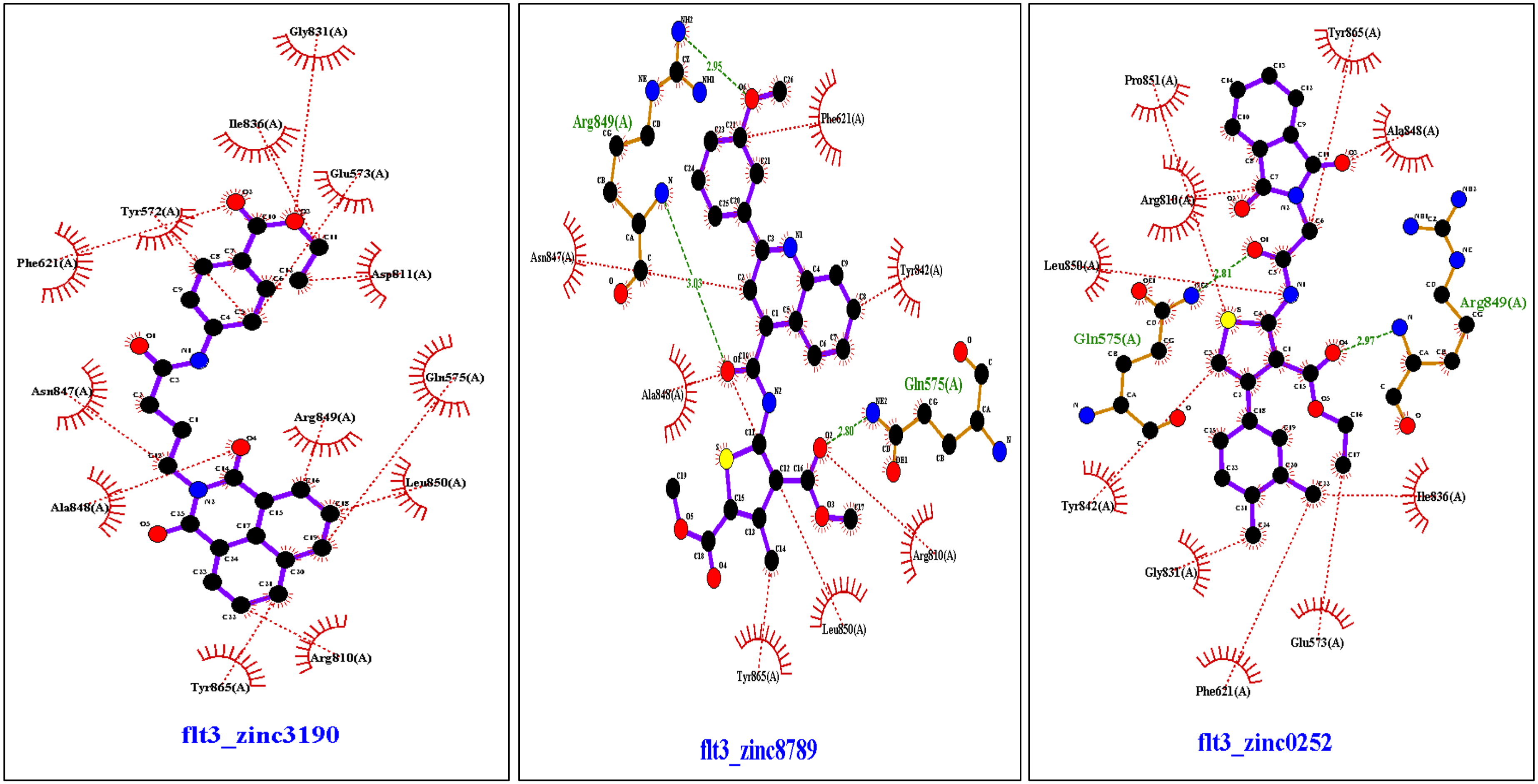
Gly831(A)
Ile836(A)
Glu573(A)
Tyr572(A)
Phe621(A)
Asp811(A)
Asn847(A)
Gln575(A)
Arg849(A)
Leu850(A)
Ala848(A)
Arg810(A)
Tyr865(A)
flt3_zinc3190
Arg849(A)
2.95
Phe621(A)
Asn847(A)
3.03
Tyr842(A)
Ala848(A)
Gln575(A)
2.80
Arg810(A)
Leu850(A)
Tyr865(A)
flt3_zinc8789
Tyr865(A)
Pro851(A)
Ala848(A)
Arg810(A)
Leu850(A)
2.81
Arg849(A)
Gln575(A)
2.97
Tyr842(A)
Ile836(A)
Gly831(A)
Glu573(A)
Phe621(A)
flt3_zinc0252

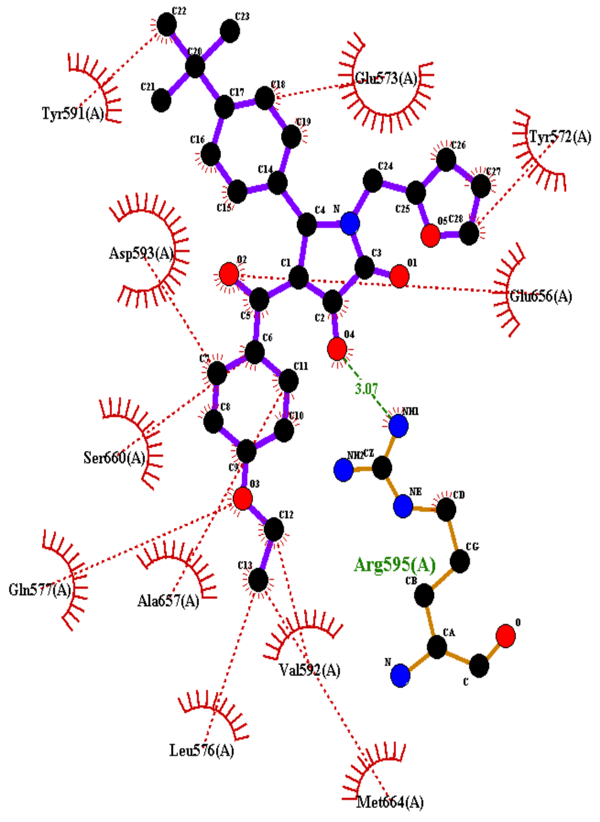

flt3_zinc2764

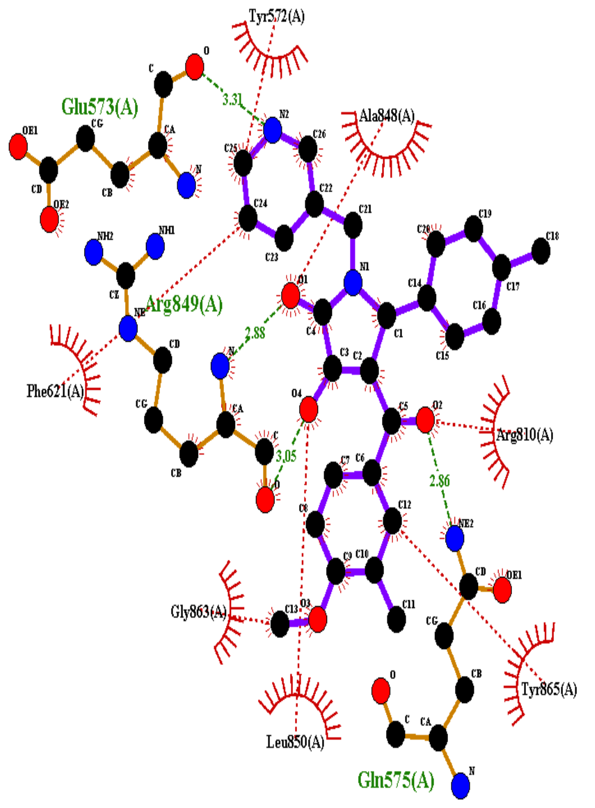

flt3_zinc5651

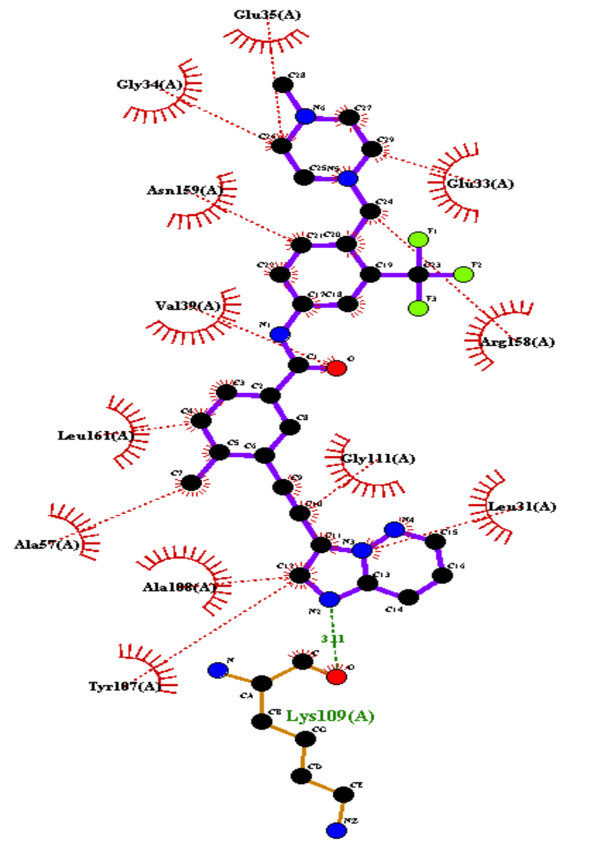

ret_ponatinib

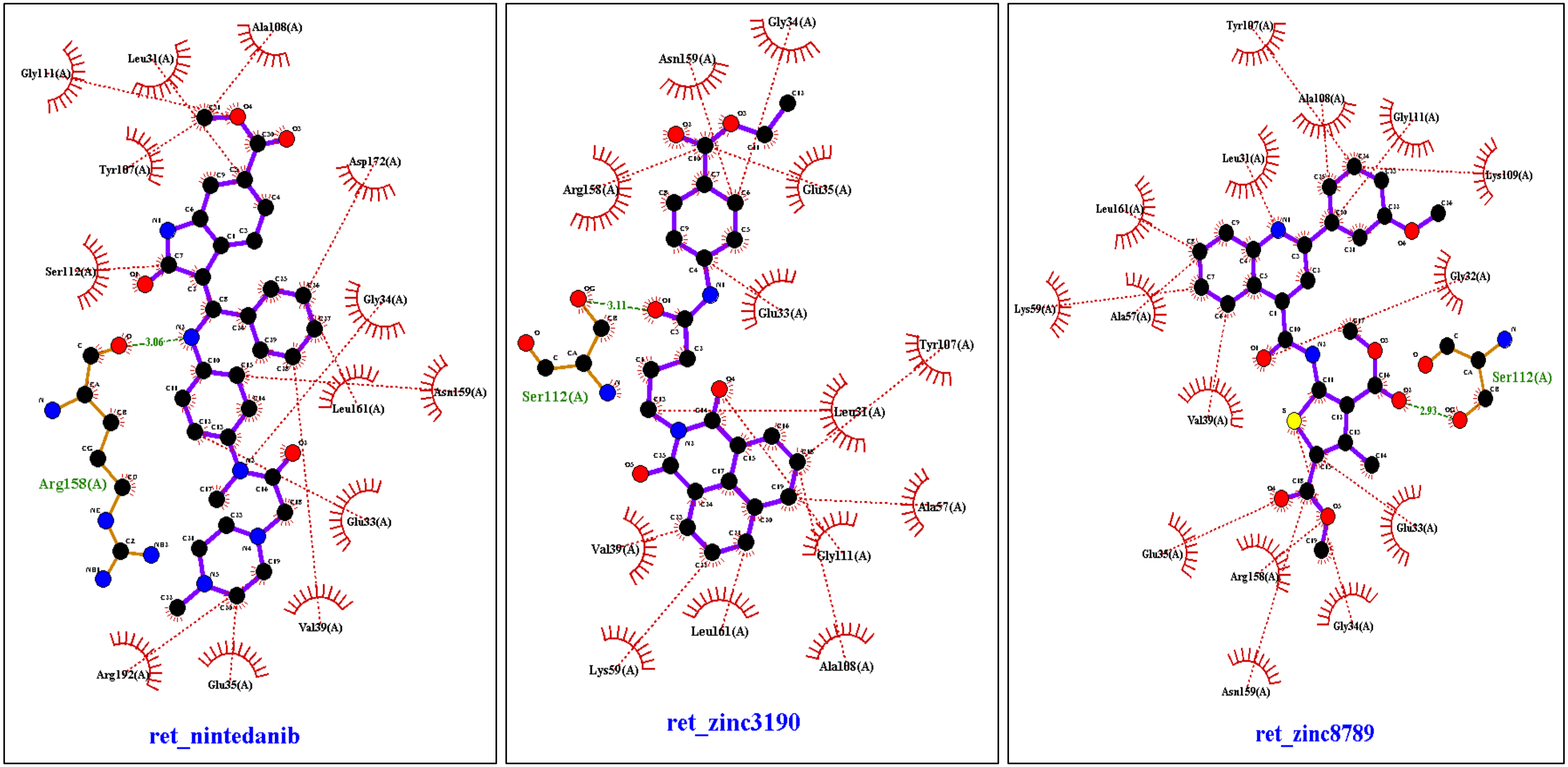
Ala108(A)
Leu31(A)
Gly111(A)
Tyr107(A)
Asp172(A)
Ser112(A)
Gly34(A)
3.06
Asn159(A)
Leu161(A)
Arg158(A)
Glu33(A)
Val39(A)
Arg192(A)
Glu35(A)
ret_nintedanib
Gly34(A)
Asn159(A)
Arg158(A)
Glu35(A)
3.11
Glu33(A)
Ser112(A)
Tyr107(A)
Leu31(A)
Ala57(A)
Val39(A)
Gly111(A)
Leu161(A)
Lys59(A)
Ala108(A)
ret_zinc3190
Tyr107(A)
Ala108(A)
Gly111(A)
Leu31(A)
Lys109(A)
Leu161(A)
Gly32(A)
Lys59(A)
Ala57(A)
Ser112(A)
Val39(A)
2.93
Glu35(A)
Glu33(A)
Arg158(A)
Gly34(A)
Asn159(A)
ret_zinc8789

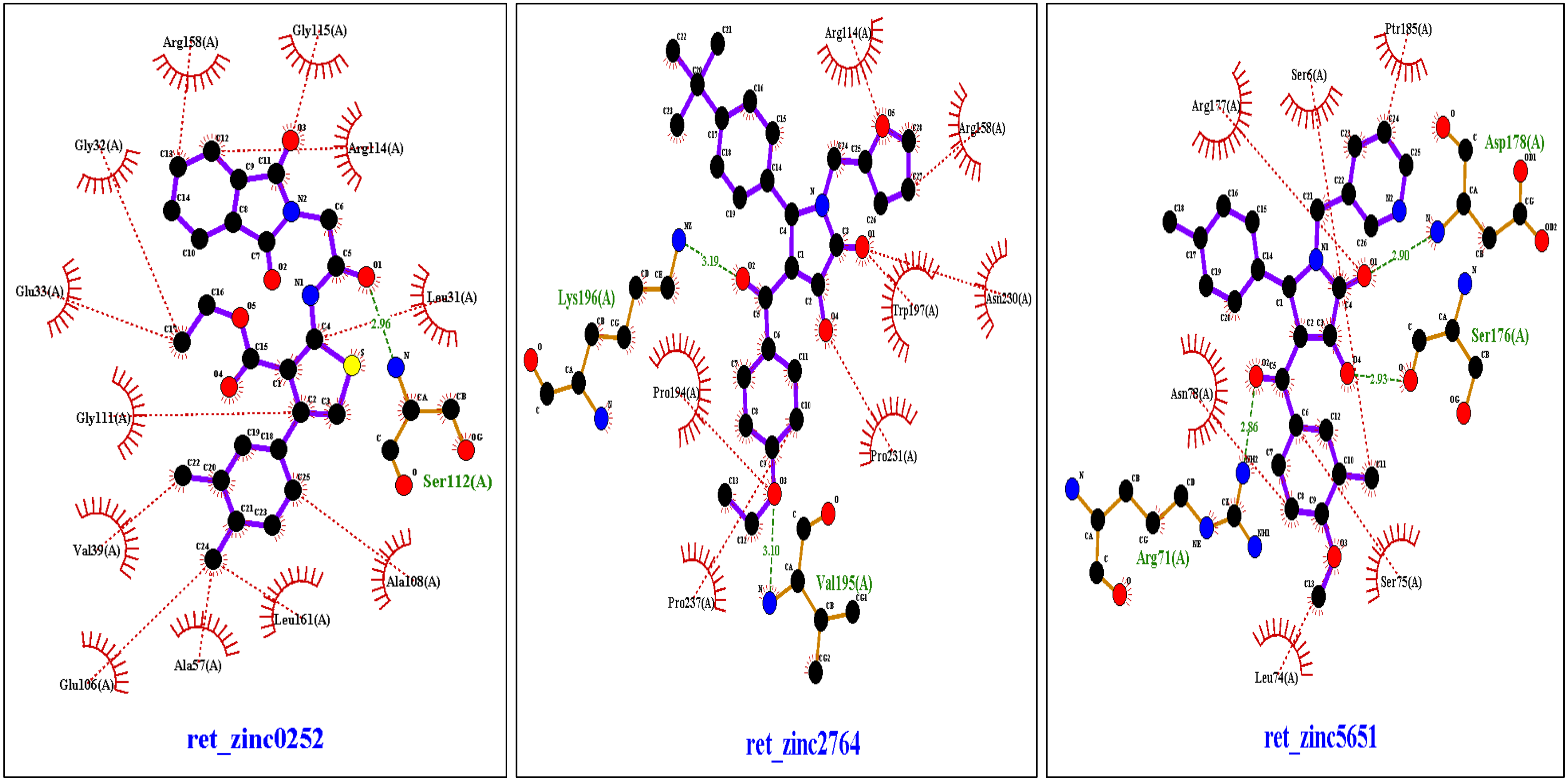

Figure S10: 2D docking structure of finalized ligands and approved drugs with ABL1 (1-7), KDR (8-13), KIT (14-20), FLT3 (21-27), RET (28-34). The green color indicates H-bonds and red color signifies hydrophobic contacts.

Table S4: molecular docking of finalized ligands with HRAS, KRAS and RB1

Type of protein	Molecule ID	Binding Affinity(kcal/mol)	Upper Bind RMSD	Lower Bind RMSD
**FGFR3**	ZINC08433190	-9.2	0.0	0.0
	ZINC00710252	-8.3	0.0	0.0
	ZINC00668789	-9.1	0.0	0.0
	ZINC09045651	-8.3	0.0	0.0
	ZINC00702764	-8.2	0.0	0.0
**HRAS**	ZINC08433190	-9.9	0.0	0.0
	ZINC00710252	-8.2	0.0	0.0
	ZINC00668789	-8.0	0.0	0.0
	ZINC09045651	-6.7	0.0	0.0
	ZINC00702764	-6.5	0.0	0.0
**KRAS**	ZINC08433190	-9.3	0.0	0.0
	ZINC00710252	-8.1	0.0	0.0
	ZINC00668789	-8.7	0.0	0.0
	ZINC09045651	-8.1	0.0	0.0
	ZINC00702764	-8.7	0.0	0.0
**RB1**	ZINC08433190	-8.5	0.0	0.0
	ZINC00710252	-7.9	0.0	0.0
	ZINC00668789	-8.0	0.0	0.0
	ZINC09045651	-8.3	0.0	0.0
	ZINC00702764	-7.1	0.0	0.0

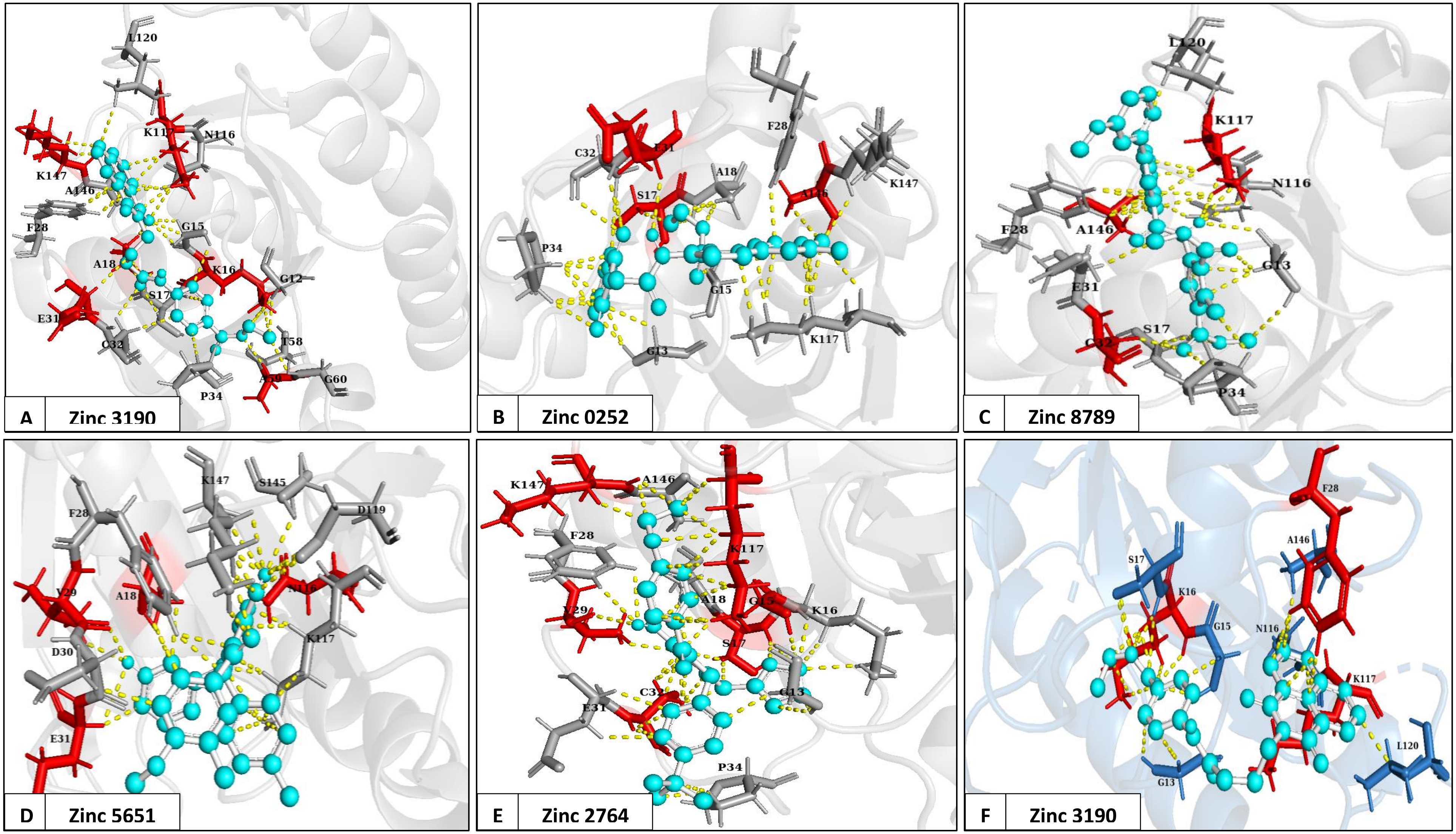

A
Zinc 3190
L120
K117
N116
K147
A146
F28
G15
A18
K16
G12
S17
E31
C32
T58
P34
G60
B
Zinc 0252
F28
C32
A18
K147
S17
P34
G15
K117
G13
C
Zinc 8789
L120
K117
N116
F28
A146
G13
E31
S17
C32
P34
D
Zinc 5651
K147
S145
D119
F28
A18
D30
K117
E31
E
Zinc 2764
K147
A146
F28
K117
A18
K16
S17
C32
E31
G13
P34
F
Zinc 3190
F28
A146
S17
K16
G15
N116
K117
L120
G13

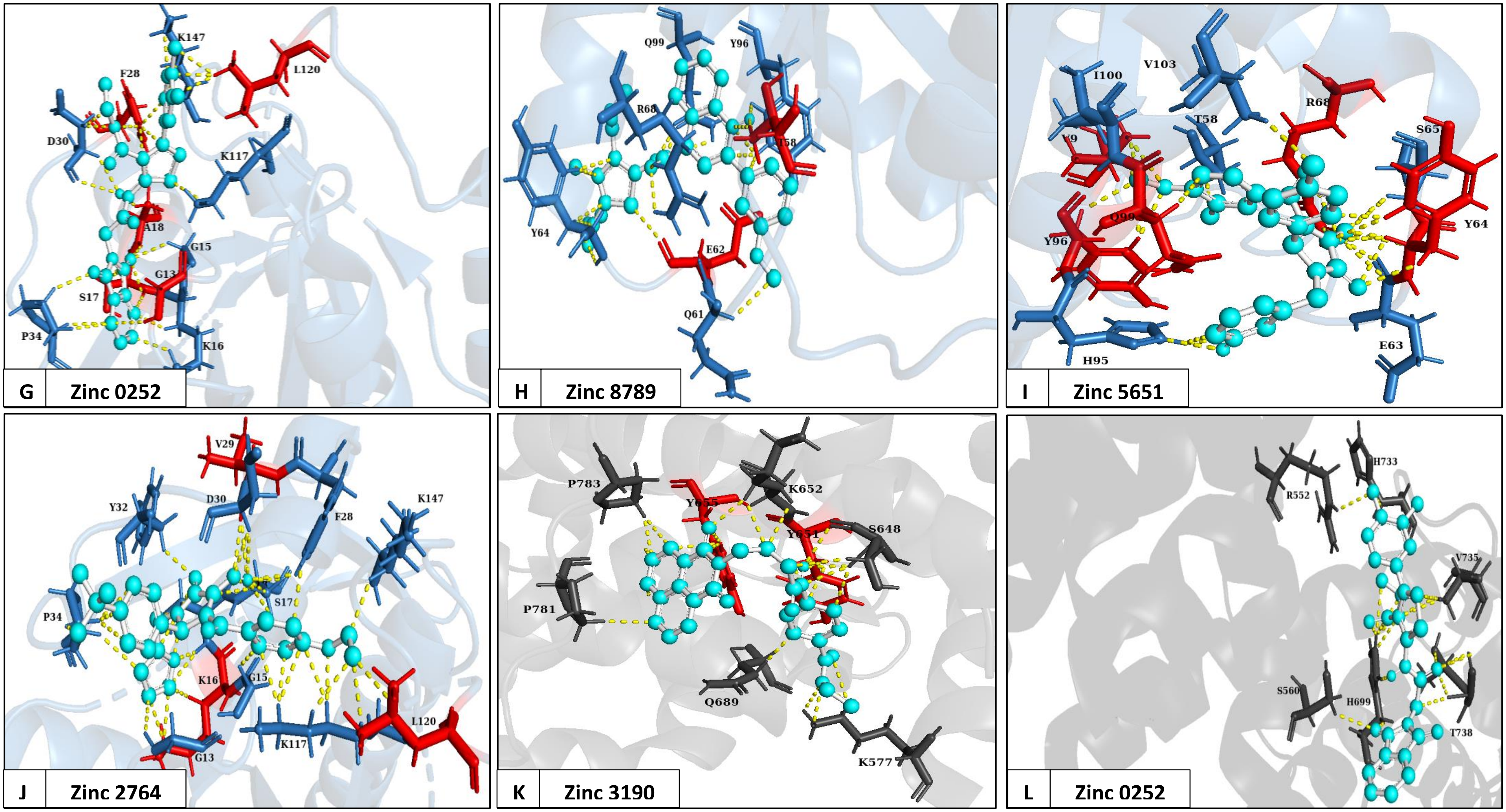

G
Zinc 0252
K147
F28
L120
D30
K117
A18
G15
G13
S17
P34
K16
H
Zinc 8789
Q99
Y96
R68
T58
Y64
E62
Q61
I
Zinc 5651
I100
V103
R68
T58
S65
V9
Q99
Y96
Y64
H95
E63
J
Zinc 2764
V29
D30
Y32
F28
K147
S17
P34
K16
G15
L120
K117
G13
K
Zinc 3190
P783
K652
S648
P781
Q689
K577
L
Zinc 0252
H733
R552
V735
S560
H699
T738

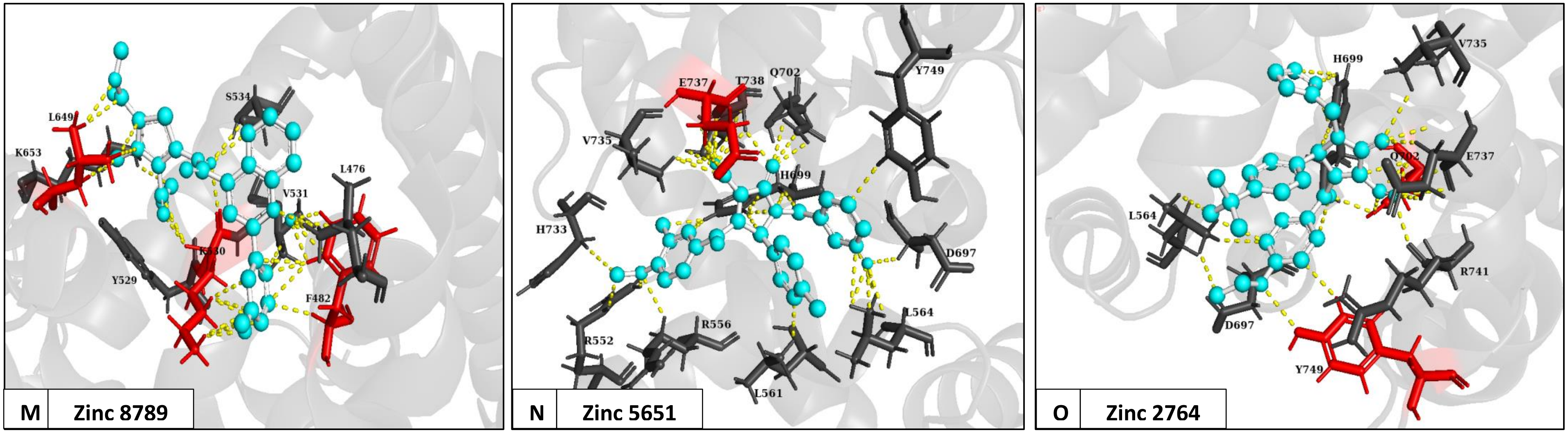

Figure S11: Prediction of docking 3D structure using Pymol software (any contacts between finalized ligands and proteins within 3Å). HRAS (A-E), KRAS (F-J), RB1 (K-O).Yellow color indicates interaction (Hydrogen & hydrophobic bonds), Cyan represents ligands. The red color used in some pictures for distinguishing the interaction bonds.

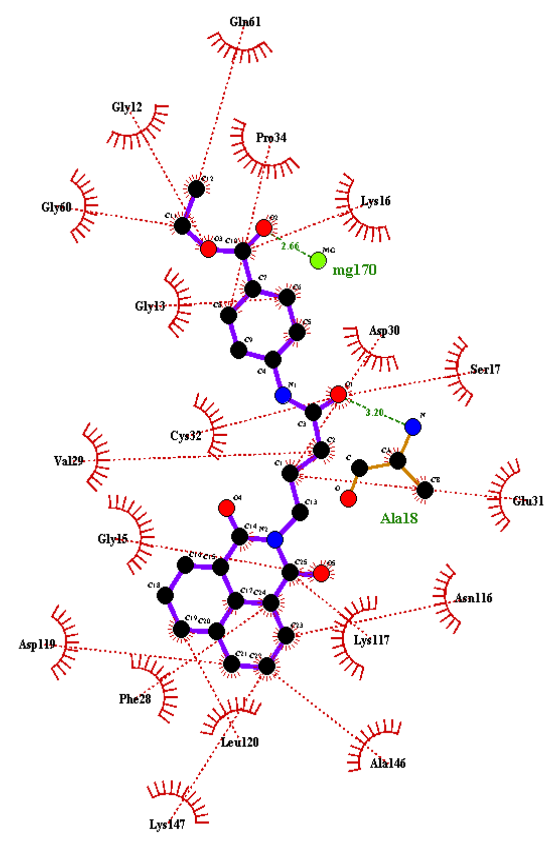

HRAS_ZINC08433190

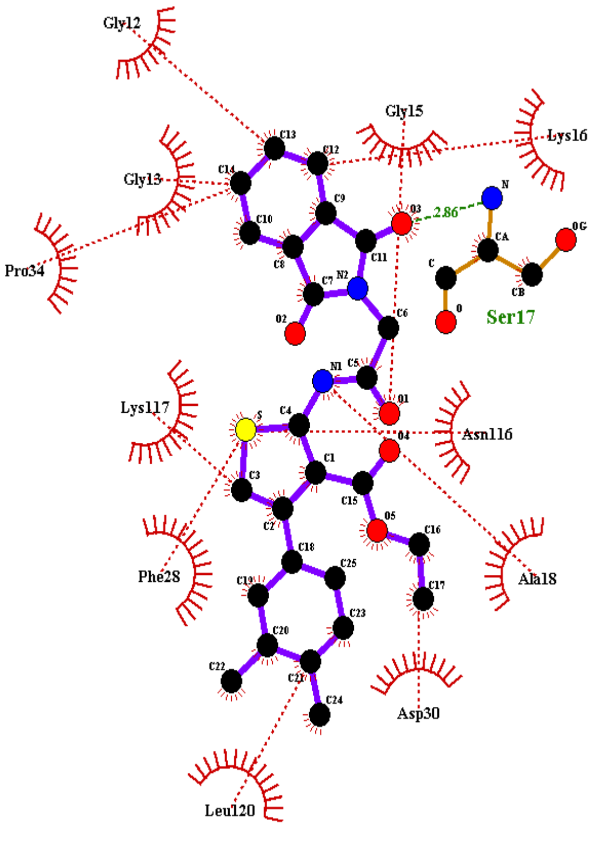

HRAS_ZINC00710252

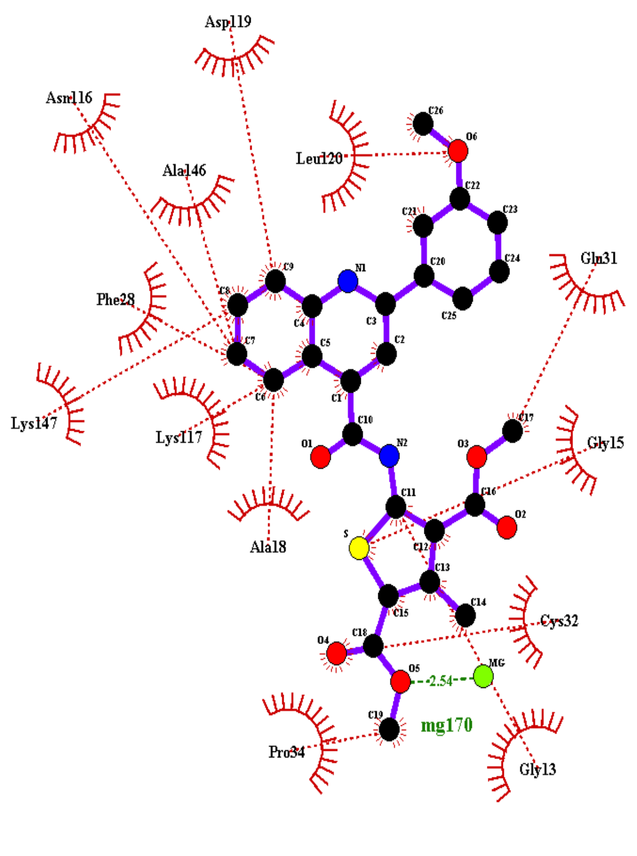

HRAS_ZINC00668789

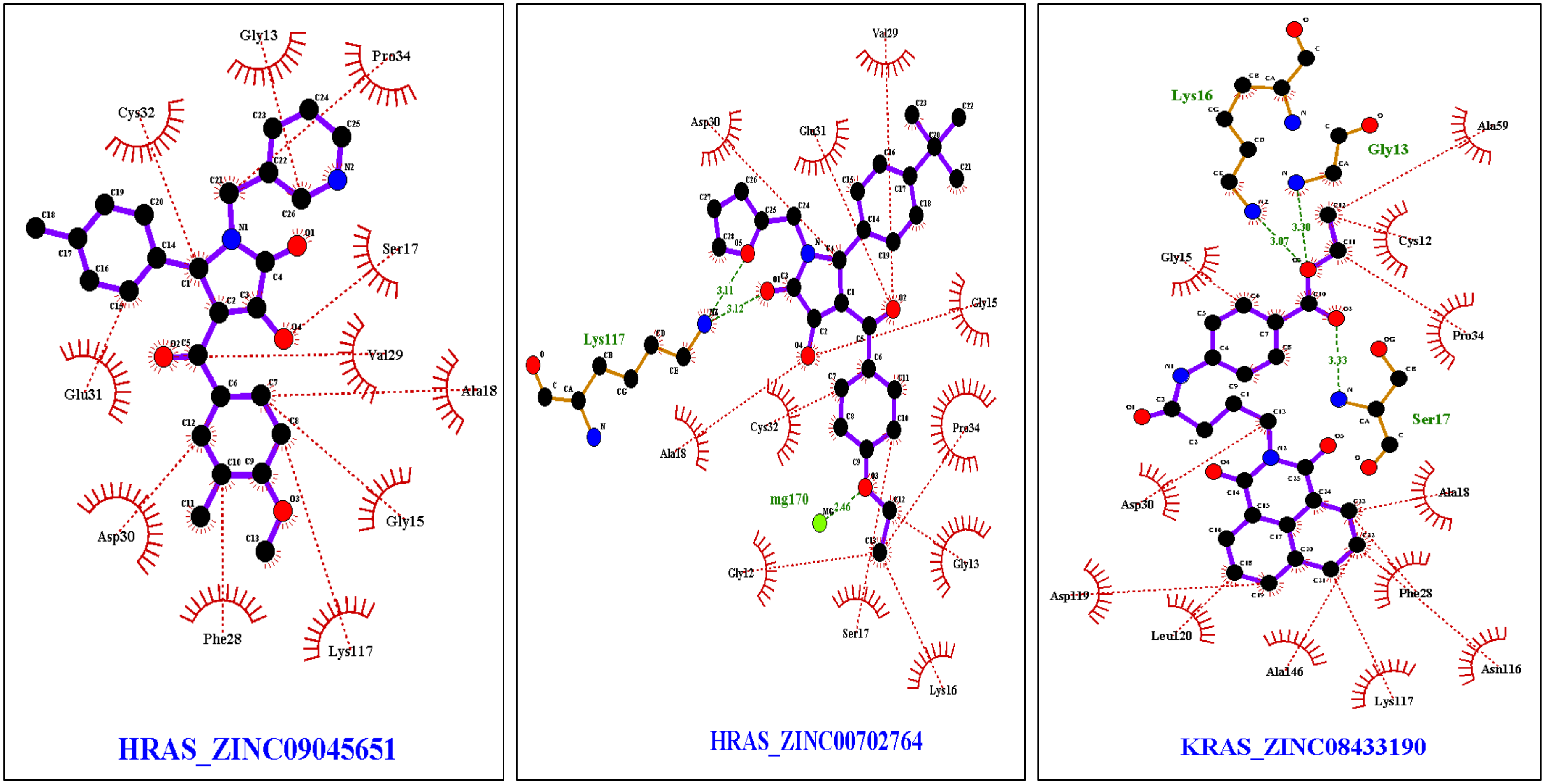
Gly13
Pro34
Cys32
Ser17
Val29
Ala18
Glu31
Gly15
Asp30
Phe28
Lys117
HRAS_ZINC09045651
Val29
Asp30
Glu31
Gly15
Lys117
3.11
3.12
Pro34
Cys32
Ala18
mg170
2.46
Gly13
Gly12
Ser17
Lys16
HRAS_ZINC00702764
Lys16
Gly13
Ala59
3.07
3.30
Cys12
Gly15
Pro34
3.33
Ser17
Asp30
Ala18
Asp119
Phe28
Leu120
Ala146
Asn116
Lys117
KRAS_ZINC08433190

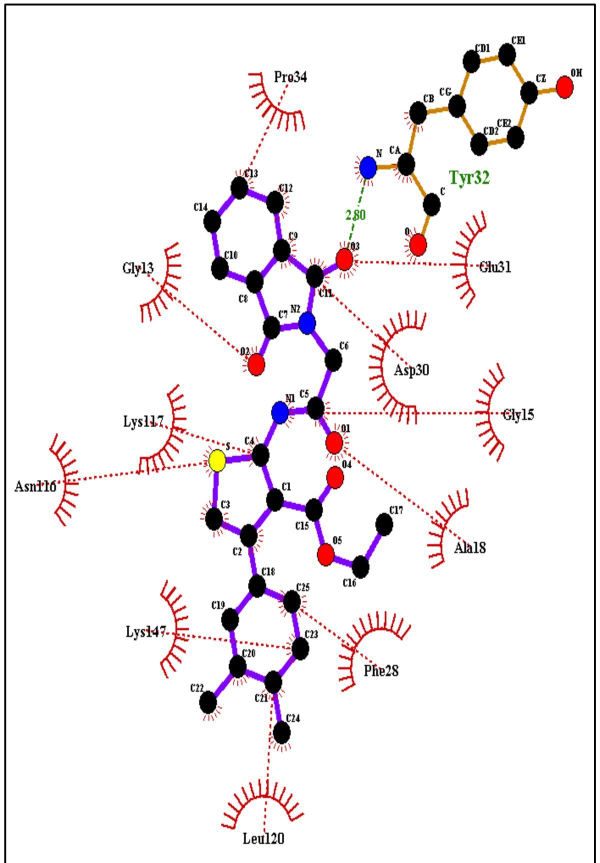

KRAS_ZINC00710252

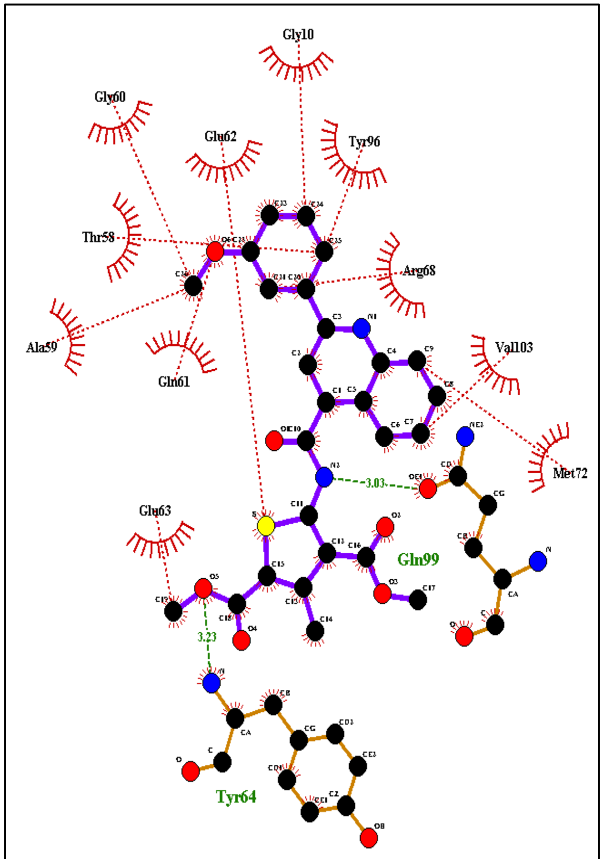

KRAS_ZINC00668789

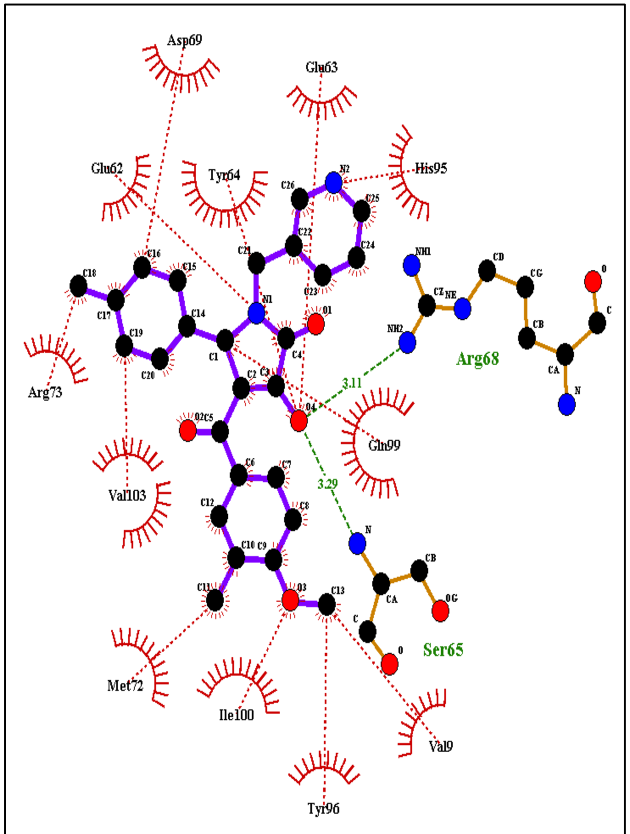

KRAS_ZINC09045651

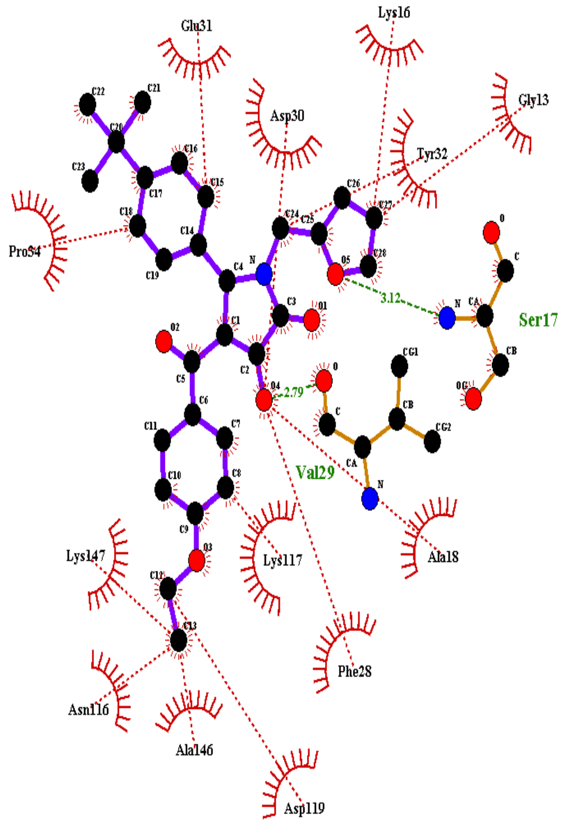

KRAS_ZINC00702764

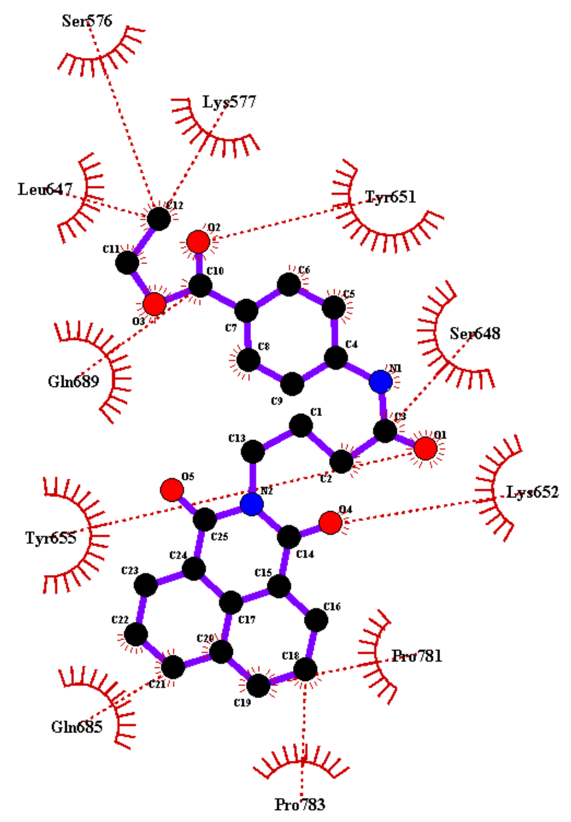

RB1_ZINC08433190

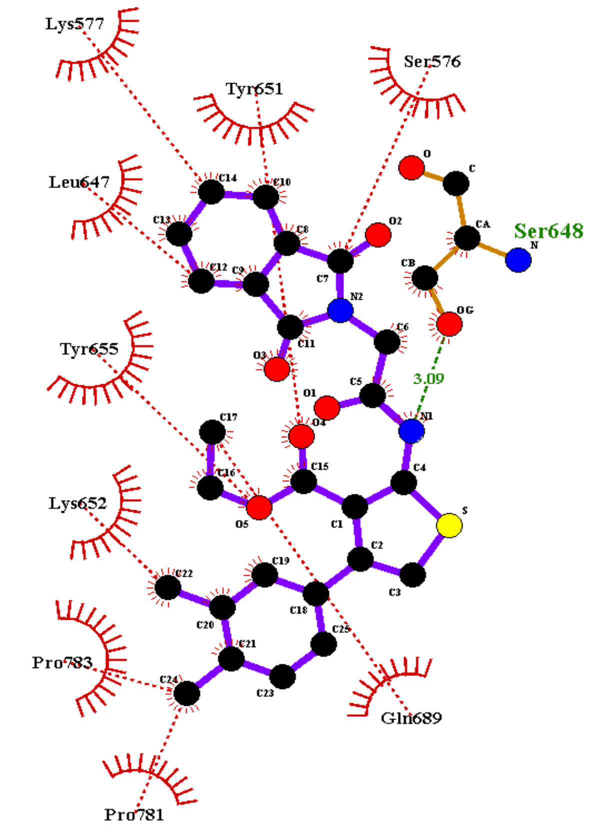

RB1_ZINC00710252

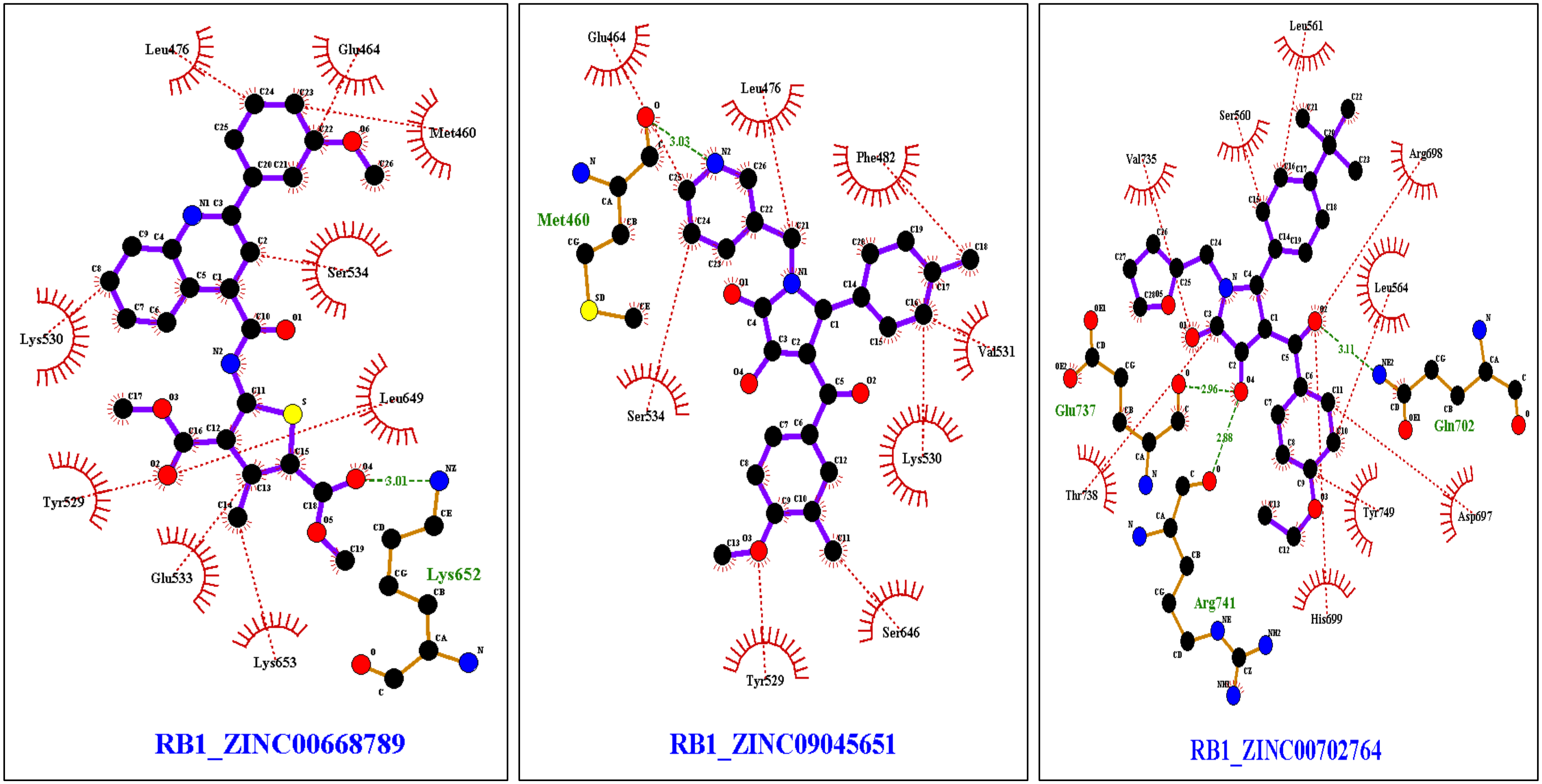

Figure S12: prediction of 2D docking structure with HRAS, KRAS, RB1using Ligplot software (H-bonds & hydrophobic contacts between finalized ligands and proteins by software defaults)
